# Detection and Molecular Characterization of Novel dsRNA Viruses Related to the *Totiviridae* Family in *Umbelopsis ramanniana*

**DOI:** 10.3389/fcimb.2019.00249

**Published:** 2019-07-11

**Authors:** Tünde Kartali, Ildikó Nyilasi, Boglárka Szabó, Sándor Kocsubé, Roland Patai, Tamás F. Polgár, Gábor Nagy, Csaba Vágvölgyi, Tamás Papp

**Affiliations:** ^1^Department of Microbiology, Faculty of Science and Informatics, University of Szeged, Szeged, Hungary; ^2^Biological Research Centre of the Hungarian Academy of Sciences, Institute of Biophysics, Szeged, Hungary; ^3^MTA-SZTE Fungal Pathogenicity Mechanisms Research Group, Department of Microbiology, University of Szeged, Hungarian Academy of Sciences, Szeged, Hungary

**Keywords:** mycovirus, dsRNA, virus particle, *Totivirus*, *Victorivirus*, *Chrysoviridae*, Mucoromycotina

## Abstract

*Umbelopsis ramanniana* is an oleaginous fungus belonging to the Mucoromycotina subphylum. Our group had previously detected four double-stranded RNA (dsRNA) bands in the *U. ramanniana* NRRL 1296 strain by gel electrophoresis. Here we describe the molecular characterization of its dsRNA elements as well as the discovery of four novel dsRNA viruses: Umbelopsis ramanniana virus 1 (UrV1), Umbelopsis ramanniana virus 2 (UrV2), Umbelopsis ramanniana virus 3 (UrV3), and Umbelopsis ramanniana virus 4 (UrV4). Full genomes of UrV1, UrV3, and UrV4 were determined using the full-length amplification of cDNAs (FLAC) technique; they contain two open reading frames (ORF), which putatively encode the coat protein (CP) and the RNA dependent RNA polymerase (RdRp), respectively. In case of UrV2, a partial ORF encoding a partial RdRp gene could be determined. Based on the phylogeny inferred from the RdRp sequences, UrV1 and UrV4 belong to the genus *Totivirus*, while UrV2 may belong to the genus *Victorivirus*. UrV3 nested to a novel, unclassified group of *Totiviridae*, which is related to the genus *Totivirus*. Hybridization analysis revealed that the dsRNA molecules of UrV1 and UrV4 correspond to the same 5.0-kbp electrophoretic band, whilst the probe for the UrV3 hybridized to the largest, 5.3-kbp and the 3.0-kbp bands of the dsRNA pattern of *U. ramanniana*. Interestingly, the probe for the UrV2 sequence did not hybridized to any dsRNA bands, but it could be amplified from the isolated 3.0-kbp fragment. By transmission electron microscopy, two different isometric virus particles with about 50 and 35 nm in diameter were detected in *U. ramanniana* NRRL 1296 indicating that this strain harbor multiple viruses. Beside *U. ramanniana*, dsRNA elements were also detected in other *Umbelopsis* isolates with different patterns consisting of 2 to 4 discrete and different sized (0.7–5.3-kbp) dsRNA molecules. Based on a hybridization analysis with UrV1 CP and RdRp probes, the bands with the size of around 5.0-kbp, which were present in all tested *Umbelopsis* strains, are presumed as possible full mycovirus genomes.

## Introduction

Mycovirus research has achieved a significant advance in the last decades and viruses have been described in all major fungal phyla (Ghabrial et al., 2015; Son et al., 2015). Vast majority of the known mycoviruses have linear dsRNA genome but linear positive- and negative-sense single-stranded RNA (ssRNA) and circular ssDNA genomes has also been described from fungi (Hillman and Cai, 2013; Ghabrial et al., 2015). Mycoviruses with dsRNA genomes are currently classified into the families *Chryso*-, *Endorna*-, *Megabirna*-, *Quadri*-, *Partiti*-, *Reo*-, and *Totiviridae* (Ghabrial et al., 2015). Among them, *Totiviridae* is the most characterized group (Ghabrial, 1998; King et al., 2011). Members of this family have isometric virions, which contain a non-segmented, linear, uncapped dsRNA molecule (King et al., 2011). The size of the genome is between 4.6 and 7.0-kbp and consists of two, usually overlapping ORFs encoding the coat protein (CP) and the RNA dependent RNA polymerase (RdRp) (King et al., 2011). Within the *Totiviridae*, the genera *Giardiavirus, Leishmaniavirus, Trichomonasvirus, Totivirus*, and *Victorivirus* are discerned. Fungal viruses are generally found in the latter two genera (Ghabrial et al., 2015). The family *Chrysoviridae* is related to the *Totiviridae* and contains viruses with segmented genomes. The *Chrysoviridae* genome typically consists of four linear dsRNA segments (with 2.4–3.6-kbp in size) encoding the CP, the RdRp, and two proteins with an unknown function (Ghabrial, 2008).

Although presence of most mycoviruses proved to be asymptomatic in their hosts, an increasing number of data indicates that it can causes smaller or greater changes in the phenotype of their fungal host (Ghabrial, 1998; Ghabrial and Suzuki, 2009; Ghabrial et al., 2015). In some cases, presence of the mycoviruses can reduce or enhance the virulence of the fungal host causing hypo- or hypervirulence, respectively (Nuss, 2005; Ghabrial et al., 2015).

Basal fungi, especially the different groups of the former Zygomycota have remained among the least explored organisms in respect of virus harboring. According to the recently accepted taxonomy, majority of the most common species generally known as zygomycetes fungi belong to the subphylum Mucoromycotina (Spatafora et al., 2016). There is only sporadic information about mycoviruses in this group. DsRNA elements and isometric, non-enveloped virus-like particles (VLPs) about 30–40 nm in diameter were earlier detected in five *Mucor* and three *Rhizopus* species without further molecular studies or sequence information (Vágvölgyi et al., 1998; Papp et al., 2001). Recently, Vainio et al. (2017) detected the presence of viral RNA sequences in various zygomycetes strains from a forest habitat identified only as *Mucor*/Mucorales, *Mortierella*/*Umbelopsis*, and “other zygomycetes” and reported a bunya-type virus in Mucorales spp. named as Mucorales RNA virus 1 (MucRV1). In addition, a geminivirus-like ssDNA virus was described and characterized in *Mucor racemosus* (Hafez et al., 2013).

*Umbelopsis* species are oleaginous fungi, which are widespread in soil and frequently isolated from the rhizosphere (Takeda et al., 2014; Spatafora et al., 2016). They constitute the monogeneric family *Umbelopsidaceae* in the order Umbelopsidales, which forms Mucoromycotina, together with Mucorales and Endogonales (Spatafora et al., 2016). Several members of the genus were previously described or referred as *Mucor, Micromucor*, or *Mortierella* species (Meyer and Gams, 2003). Although no mycoviruses have hitherto been observed in the representatives of this genus, Vágvölgyi et al. (1993, 1998) had earlier reported the detection of four or five dsRNA bands in the *Umbelopsis ramanniana* (*Mucor ramannianus*) strain NRRL 1296 by gel electrophoresis. In the present study, detection of virus particles in this strain as well as sequencing and characterization of its dsRNA elements were carried out. A screening to detect the UrV1 or related viral sequences in other *Umbelopsis* isolates representing various species was also performed.

## Materials and Methods

### Fungal Strains and Cultivation

Twenty-four strains representing 11 *Umbelopsis* species (see Supplementary Table S1) were tested for the presence of dsRNA molecules. The strains were maintained on malt extract agar slants (0.5% malt extract, 0.5% yeast extract, 1% glucose, 2% agar) at 4°C. Mycelia for virus particle and dsRNA purification were grown in yeast extract-glucose broth (1% glucose, 0.5% yeast extract) at 25°C for 3 days.

### Isolation of dsRNA Molecules

To screen for the presence of dsRNA elements, a variation of the lithium chloride-based total nucleic acid extraction method of Leach et al. (1986) was used. Frozen mycelia (300 mg) were powdered under liquid nitrogen in a mortar and incubated in 700 μl ice-cold LETS buffer (0.1 M LiCl, 10 mM EDTA, 10 mM Tris-HCl, 0.5% SDS, pH 8.0) and 70 μl 10% SDS for 2 min at room temperature with continuous vortexing. The samples were then centrifuged at 17,000 × *g* for 15 min at 4°C. The supernatant was purified with phenol:chloroform:isoamyl alcohol (25:24:1) extraction twice and one extraction was done by chloroform:isoamyl alcohol (24:1). Then, nucleic acids were precipitated from the upper phase with two volumes of 96% ethanol and 10% sodium-acetate overnight at -70°C. After centrifugation of the samples at 17,000 × *g* for 20 min at 4°C, nucleic acids were washed with 75% ethanol and dried under vacuum, then resuspended in 100 μl AccuGene molecular biology water (Lonza).

CF-11 cellulose chromatography was used to purify dsRNA elements from total nucleic acid extracts purified with LETS buffer extraction. The purification was done according to the method of Morris and Dodds (1979) with minor modifications. After supplementing 100 μl of total nucleic acid extracts with 3 ml 16% ethanolic STE buffer (0.1 M NaCl, 0.05 M TrisHCl, 0.001 M EDTA, pH 8.0), 0.2 g CF-11 cellulose (Sigma-Aldrich) was added to the samples. These mixtures were carried up to a home-made column, which contained 0.2 g CF-11 cellulose and was previously washed with 5 ml 16% ethanol and 2% β-mercaptoethanol containing STE buffer. The samples were then percolated on the cellulose columns. The columns were washed with 5 ml 16% ethanol containing STE buffer for four times, then dsRNA molecules were eluted from the cellulose column with 2 ml STE buffer. After ethanolic precipitation and centrifugation (17,000 × *g*, 20 min, 4°C), the samples were dried under vacuum and resuspended in 100 μl AccuGene molecular biology water (Lonza).

All dsRNA samples were separated by electrophoresis on 0.8% agarose/TAE (40 mM Tris/acetic acid, 1 mM EDTA, pH 7.6) horizontal gels. Nucleic acids were visualized by UV fluorescence after ethidium bromide (0.5 μg/ml) staining. The relative sizes of the dsRNA molecules were estimated using GeneRuler 1-kb DNA ladder (Thermo Scientific) as size standards. The nature of the detected dsRNA elements was confirmed by their resistance to DNase I (Thermo Scientific) and S1 nuclease (Thermo Scientific) digestions, which were carried out according to the recommendations of the manufacturers'.

### Purification and Examination of the Virus Particles

Virus particles were purified from frozen mycelium according to the method of Lot et al. (1972). After the disruption of the mycelium under liquid nitrogen in a mortar with pestle, it was extracted with 25 ml citrate buffer (0.5 M trisodium citrate, 1 mM disodium EDTA, pH 6.5), 40 μl chloroform and 120 μl sodium thioglycolate. This solution was centrifuged (9,000 rpm, 30 min, 4°C) and the aqueous phase was supplemented with 10% polyethylene glycol 6,000 (VWR) and 0.5 M sodium chloride and then incubated for overnight at 4°C. After a centrifugation (10,000 rpm, 30 min, 4°C), the pellets were resuspended in 10 ml borate buffer (5 mM boric acid, 1.475 mM sodium tetraborate, 0.5 mM EDTA) and 300 μl Triton X-100 (Sigma-Aldrich) and incubated for 30 min at room temperature. After a subsequent centrifugation (12,000 rpm, 15 min, 4°C), the virus particles were recovered by pelleting them from the supernatant with an ultracentrifugation at 78,000 × *g* for 10 h at 4°C. Finally, the pellet was resuspended in 120 μl borate buffer.

Purified virus particles were analyzed with transmission electron microscopy. Samples were evaluated under a JEM-1400 Flash transmission electron microscope (JEOL) to identify the morphological characteristics of the particles. To obtain the negatively stained samples, 10 μl of the virus particle extract was mounted on a formvar-coated 150-mesh copper grid (Electron Microscopy Sciences). After 5 min, the excessive fluid was blotted away with the edge of a filter paper and the samples were contrasted with 10 μl 2% uranyl acetate (Electron Microscopy Sciences) in 50% ethanol for 5 min (3 times). After the removal of the excessive staining solution, samples were dried under a Petri dish for 2 h before the electron microscopic evaluation. Negatively stained samples were systematically screened at 30,000 × magnification to localize the presence of the virus particles on the grid. Afterwards, the particles were recorded at 40,000–60,000 × magnification with a 16 MP Matataki Flash scientific complementary metal–oxide–semiconductor (sCMOS) camera (JEOL).

### cDNA Synthesis and Sequencing of the dsRNA Molecules

For the synthesis and amplification of cDNAs from the dsRNA templates the “Full-length amplification of cDNAs” (FLAC) technique (Maan et al., 2007) was used. Purification of the dsRNA fragments was performed with the RNaid kit (MP Biomedicals). Ligation of the PC3-T7 loop primer (5'-p-GGATCCCGGGAATTCGGTAATACGACTCACTATATTTTTATAGTGAGTCGTATTA-OH-3'; Potgieter et al., 2009) to the purified dsRNA fragments, the denaturation of the primer-ligated dsRNAs and cDNA synthesis reaction were performed as described by Darissa et al. (2010). Amplification of the cDNA was performed using 1.25 μM PC2 primer (5'-CCGAATTCCCGGGATCC-3'; Potgieter et al., 2009) and 2.5 units of the Phusion High-Fidelity DNA Polymerase (Thermo Scientific). The PCR was incubated in a MJ Mini 48-Well Personal Thermal Cycler (Bio-Rad) at 72°C for 2 min and 98°C for 1 min, followed by 35 cycles of 98°C for 10 s, 66°C for 30 s and 72°C for 4 min, and a final elongation at 72°C for 10 min. PCR products were purified from the agarose gel with the Zymoclean Large Fragment DNA Recovery Kit (Zymo Research). Purified products were then cloned into the pJET1.2/Blunt vector (CloneJET PCR Cloning Kit, Thermo Scientific). Sequences of the inserts were determined by the LGC Genomics (Germany). The sequences were then subjected to BLAST searches (http://blast.ncbi.nlm.nih.gov/Blast.cgi) in the nucleic acid and protein databases of the National Center for Biotechnology Information (NCBI). The identified sequences were deposited to European Nucleotide Archive (ENA; accession numbers: LR216267-LR216269 and LR595925-LR595928). All sequences are also presented in Supplementary Data S1.

### Hybridization Studies

For hybridization, dsRNAs and control plasmids were separated by electrophoresis on 1.0% agarose/TAE (40 mM Tris/acetic acid, 1 mM EDTA, pH 7.6) horizontal gels. The relative sizes of the dsRNA molecules were estimated using DIG-labeled DNA Molecular Weight Marker VII (Roche) as size standards. After gel electrophoresis, gel was divided, since dsRNA and DNA samples were denatured under different conditions. Accordingly, dsRNAs were denatured in 0.05 M sodium hydroxide and 0.15 M sodium chloride buffer for 30 min and neutralized in 1 M Tris-hydrochloride and 1.5 M sodium chloride buffer (pH 7.5) for 2 × 20 min as described by Hong et al. (1998). DNA samples (i.e., the control plasmids) were denatured in 0.5 M sodium hydroxide and 1.5 M sodium chloride buffer and neutralized in 0.5 M Tris and 1.5 M sodium chloride buffer (pH 7.5). Gel slides were blotted onto a positively-charged nylon membrane (Amersham Hybond-N+, GE Healthcare) with 2 × SSC buffer. Samples were allowed to dry at room temperature and immobilized with UV-crosslinking. Blots were hybridized with the UrV1 CP and RdRp, UrV2 RdRp, UrV3 RdRp, UrV4 CP, and UrV4 RdRp gene probes in hybridization buffer (0.9 M sodium chloride, 1% SDS, 10% dextran sulfate) containing 5 μg/ml salmon sperm DNA (Invitrogen). Probes were prepared by PCR from DNA templates in the presence of digoxigenin-UTP (DIG DNA Labeling Mix, Roche) using DreamTaq polymerase (Thermo Scientific). Primers used to amplify the probes are listed in the Supplementary Table S2. Hybridization was followed by immunological detection using alkaline phosphatase-conjugated anti-digoxigenin antibody (Roche). Reactions for detection were carried out according to the manufacturer's instructions (Roche).

### Sequence and Phylogenetic Analysis

Representative sequences of the families *Totiviridae, Chrysoviridae*, and *Partitiviridae* were obtained from the viruSite (http://www.virusite.org/index.php; Stano et al., 2016). The corresponding accession numbers are indicated on the tree. The dataset was supplemented by homologous hits of *U. ramanniana* RdRp sequences derived from BLAST (https://blast.ncbi.nlm.nih.gov/Blast.cgi) search. Multiple sequence alignment was carried out by MAFFT v7.312 using the E-INS-i option (Katoh and Standley, 2013) (for the alignment see Supplementary Data S2). Maximum Likelihood (ML) analysis was conducted by RAxML v8.121 (Stamatakis, 2014) using the WAG model with GAMMA rate heterogeneity and statistical support of the results was obtained by 500 thorough bootstrap replicates. Alternatively, phylogeny was also reconstructed with the Neighbor Joining (NJ) method using 500 bootstrap replicates. Sequences of the whole ITS region of *Umbelopsis* isolates were aligned using MAFFT v7.312 with the E-INS-i iterative refinement method. NCBI accession numbers for the involved sequences are available in the Supplementary Table S1. The ITS region of *Mortierella polycephala* (NCBI accession no.: HQ630335) was used as an outgroup to root the tree. ML tree was constructed by using RAxML v8.121 with 1,000 bootstrap replicates under the GTR model with gamma distributed rate heterogeneity. To determine the conserved motifs in the RdRp amino acid sequences, the alignment was performed using the Clustal Omega program at the website of the European Bioinformatics Institute (EMBL-EBI; https://www.ebi.ac.uk/Tools/msa/clustalo/). The alignment is presented in Supplementary Data S3. Putative proteins were predicted and analyzed using the tools of the Expasy Bioinformatics Resource Portal (https://www.expasy.org/). The HPknotter program (http://genome.cs.nthu.edu.tw/HPKNOTTER/; Huang et al., 2005) was used to predict possible RNA H-type pseudoknots. Molecular weights of the identified proteins were predicted with the Protein Molecular Weight program (https://www.bioinformatics.org/sms/prot_mw.html).

## Results

### Screening for the Presence of dsRNA Elements in *Umbelopsis* Strains

**Figure 1:**
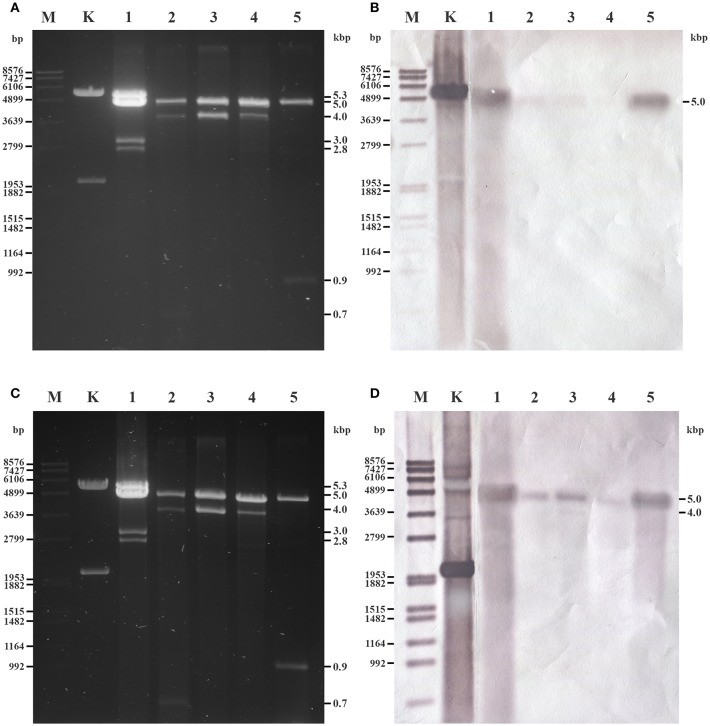
Gel electrophoretic pattern and Northern blotting of dsRNA fragments purified from the mycovirus-harboring *Umbelopsis* strains. **(A,C)** Agarose gel electrophoresis of *Umbelopsis* dsRNA fragments. Lane M, DIG-labeled DNA Molecular Weight Marker VII (Roche); Lane K, KpnI digested control plasmid containing the PCR amplicon of the UrV1 genome; Lane 1, *U. ramanniana* NRRL 1296; Lane 2, *U. gibberispora* CBS 109328; Lane 3, *U. angularis* CBS 603.68; Lane 4, *U. dimorpha* CBS 110039; Lane 5, *U. versiformis* CBS 473.74. Right numbers indicate the sizes (kbp) of the detected dsRNA fragments. **(B,D)** Northern blot analysis of *Umbelopsis* dsRNA fragments hybridized with UrV1 CP and UrV1 RdRp probes, respectively. UrV1 CP probes gave strong hybridization signal with the 5.0-kbp fragments of *U. ramanniana* (Lane 1) and *U. versiformis* (Lane 5) and weakly hybridized to the 5.0-kbp fragments of *U. gibberispora* (Lane 2) and *U. angularis* (Lane 3). UrV1 RdRp probes gave hybridization signal with the 5.0-kbp fragments of all strains and hybridized weakly to the 4.0-kbp dsRNA fragment of *U. angularis*. In case of the KpnI digested control plasmid, the 5.5-kbp bands gave strong hybridization signal with the UrV1 CP, and the 2.0-kbp bands gave strong hybridization signal with the UrV1 RdRp probe.

Twenty-four fungal isolates representing 11 species were screened and dsRNA molecules were detected in five strains belonging to the species *Umbelopsis angularis, Umbelopsis dimorpha, Umbelopsis gibberispora, U. ramanniana*, and *Umbelopsis versiformis* (Table 1 and Figures 1A,C). All strains contained different dsRNA patterns consisting of 2–4 discrete bands with estimated sizes ranging from 0.7 to 5.3-kbp (Table 1 and Figures 1A,C). Although all patterns proved to be different, each of them included a band with a similar size (~5.0-kbp).

**Table 1:** The dsRNA patterns of the mycovirus-harboring *Umbelopsis* isolates.

**Species name**	**Collection number**	**Substrate/Origin**	**dsRNA size (kb)**
*Umbelopsis ramanniana*	NRRL 1296	–/Wisconsin, USA	5.3; 5.0; 3.0; 2.8
*Umbelopsis gibberispora*	CBS 109328	*Fagus crenata*/Japan	5.0; 4.0; 0.7
*Umbelopsis angularis*	CBS 603.68	Soil/Baarn, Netherlands	5.0; 4.0
*Umbelopsis dimorpha*	CBS 110039	Soil/New Zealand	5.0; 4.0; 2.8
*Umbelopsis versiformis*	CBS 473.74	Soil/Victoria, Australia	5.0; 0.9

**Figure 2:**
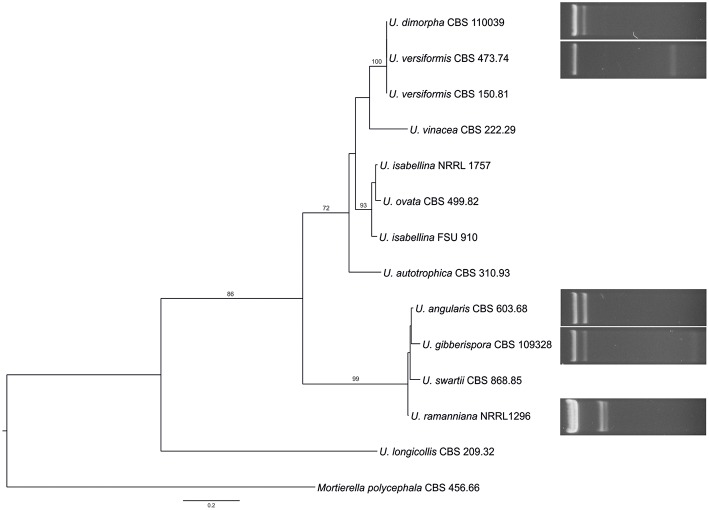
Comparison of the phylogeny of the tested *Umbelopsis* species inferred from the ITS region with the mycovirus-harboring feature. The presented tree was constructed by the Maximum Likelihood method using the RAxML program. Bootstrap values (%) indicated on branches were obtained with 1,000 replicates. *Mortierella polycephala* CBS 456.66 was used as the outgroup. The list of the investigated *Umbelopsis* strains and the accession number of the applied sequences can be seen in Supplementary Table S1.

The dsRNA harboring feature was compared with the phylogeny of the examined *Umbelopsis* strains inferred from their ITS sequence data (Figure 2). The location of the dsRNA harboring species in the phylogenetic tree and the similarity of the dsRNA patterns raise the question whether some of the dsRNA molecules would be related or of the same origin.

### Sequence Analysis of the dsRNA Elements in the *U. ramanniana* NRRL 1296 Strain

**Figure 3:**
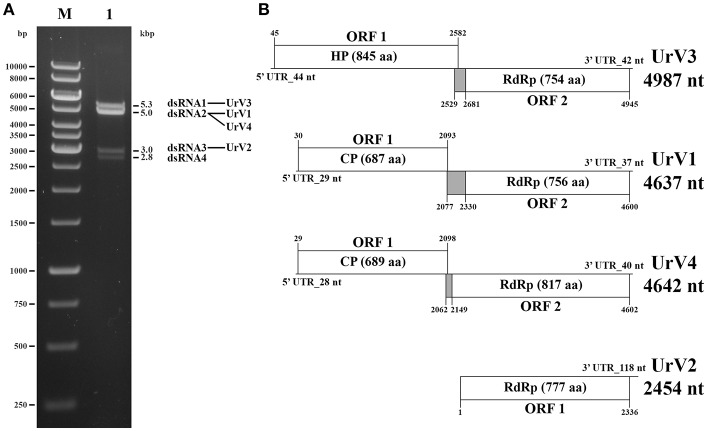
Gel electrophoretic pattern and genomic organization of *U. ramanniana* NRRL 1296 dsRNA fragments. **(A)** Agarose gel electrophoresis of dsRNA fragments purified from the mycovirus-harboring *U. ramanniana* NRRL 1296 strain. Lane M, GeneRuler 1-kb DNA Ladder (Thermo Scientific); Lane 1, *U. ramanniana* NRRL 1296. The sizes (kbp) of the detected dsRNA fragments and the corresponding virus genomes are indicated as well. **(B)** Genomic organization of the four detected dsRNA genomes showing putative open reading frames (ORFs). The first codons of the possible pseudoknot sequences are also marked. The gray boxes indicate the probable beginning of the fusion protein and the spacer region. HP, hypothetical protein; CP, coat protein; RdRp, RNA dependent RNA polymerase.

In the extracts of this fungus, four dsRNA fragments with the estimated sizes of 5.3, 5.0, 3.0, and 2.8-kbp could be detected by gel electrophoresis (Figure 3A). From the total dsRNA extract, cDNA was generated by using the FLAC technique and the resulted clones were sequenced in both directions. Four clones contained fragments corresponding to possible dsRNA/mycovirus genomes. One of them carried a fragment determined to be 4,637-nt in length. It contained two open reading frames (ORF1 and ORF2) in different frames, with a 236-nt spacer in between. Genome organization is presented in Figure 3B. ORF1 (from 30 to 2,093-nt; EMBL accession number: LR216267) encodes a putative, 687-aa coat protein (CP) while ORF2 (from 2,330 to 4,600-nt; EMBL accession number: LR216268) was predicted to encode a putative, 756-aa RNA-dependent RNA polymerase (RdRp). The predicted molecular weights of the proteins were found to be 77.64 and 87.13-kDa for the CP and the RdRp, respectively. BLASTp homology search with the corresponding fragment sequence in the NCBI GenBank revealed a high degree of identity with the CP and RdRp of viruses in the *Totiviridae* family; best matches are presented in Table 2. The highest similarity was found to the Wuhan insect virus 26 and 27 for both the CP and the RdRp. We suggest that this dsRNA segment corresponds to a genomic component of a novel mycovirus in the *Totiviridae* family, and we have tentatively named it as Umbelopsis ramanniana virus 1 (UrV1).

**Table 2:** Amino acid sequence identities of the UrV1 CP, UrV1 RdRp, UrV2 RdRp, URV3 ORF1, UrV3 RdRp, UrV4 CP, and UrV4 RdRp with similar databank sequences deduced by BLAST search.

**Virus**	**Virus name**	**Accession number**	**Identity (%)**	**E-Value**	**Query coverage (%)**
UrV1 CP	Wuhan insect virus 26 hypothetical protein 1	YP 009342427.1	44.26	5e-162	78
	Wuhan insect virus 27 hypothetical protein 1	YP 009342433.1	37.12	7e-151	95
	Red clover powdery mildew-associated totivirus 4 CP	BAT62483.1	33.18	4e-123	96
	Xanthophyllomyces dendrorhous virus L1B CP	YP 009507834.1	31.79	2e-111	100
	Hortaea werneckii totivirus 1 CP	AZT88646.1	30.67	5e-109	94
UrV1 RdRp	Wuhan insect virus 26 hypothetical protein 2	YP 009342428.1	57.14	0	99
	Wuhan insect virus 27 hypothetical protein 2	YP 009342434.1	48.93	0	99
	Delisea pulchra totivirus IndA RdRp	AMB17470.1	48.10	0	92
	Xanthophyllomyces dendrorhous virus L1B RdRp	YP 009507835.1	42.93	0	98
	Xanthophyllomyces dendrorhous virus L1A RdRp	YP 007697651.1	41.11	0	97
UrV2 RdRp	Thelebolus microsporus totivirus 1 RdRp	AZT88643.1	50.78	0	98
	Tolypocladium ophioglossoides totivirus 1 RdRp	AZT88645.1	46.89	0	99
	Ustilaginoidea virens RNA virus 5 RdRp	YP 009182167.1	46.73	0	99
	Helicobasidium mompa totivirus 1–17 RdRp	NP 898833.1	42.24	0	97
	Beauveria bassiana victorivirus NZL/1980 RdRp	YP 009032633.1	43.22	0	98
UrV3 ORF1	No significant similarity found				
UrV3 RdRp	Beihai barnacle virus 15 hypothetical protein 2	YP 009333150.1	31.05	1e-65	80
	Diatom colony associated dsRNA virus 17 genome type B RdRp	YP 009551502.1	30.12	3e-63	85
	Diatom colony associated dsRNA virus 17 genome type A RdRp	YP 009551504.1	29.62	8e-63	85
	Hubei toti-like virus 5 hypothetical protein	YP 009336942.1	27.80	1e-44	77
	Hubei toti-like virus 6 hypothetical protein 2	APG75978.1	33.26	1e-43	57
UrV4 CP	Wuhan insect virus 26 hypothetical protein 1	YP 009342427.1	43.17	3e-150	78
	Wuhan insect virus 27 hypothetical protein 1	YP 009342433.1	38.02	2e-148	94
	Red clover powdery mildew-associated totivirus 4 CP	BAT62483.1	31.89	9e-112	96
	Hortaea werneckii totivirus 1 CP	AZT88646.1	32.01	1e-107	91
	Xanthophyllomyces dendrorhous virus L1A CP	YP 007697650.1	31.44	6e-107	96
UrV4 RdRp	Wuhan insect virus 26 hypothetical protein 2	YP 009342428.1	56.62	0	96
	Wuhan insect virus 27 hypothetical protein 2	YP 009342434.1	47.76	0	95
	Delisea pulchra totivirus IndA RdRp	AMB17470.1	45.34	0	85
	Hortaea werneckii totivirus 1 RdRp	AZT88647.1	43.28	0	94
	Xanthophyllomyces dendrorhous virus L1A RdRp	YP 007697651.1	42.04	0	91

Another clone contained a 2,454-nt fragment with a partial ORF (from 1 to 2,336-nt; EMBL accession number: LR216269) encoding a partial putative RdRp protein (Figure 3B). Within the sequence the RdRp_4 (pfam: 02123) could be identified (nt 177–1,583) and the putative active site (nt 1,098–1,380) could also be predicted. BLASTp search also indicated high similarity to members of the *Totiviridae*, with the best match to the RdRp of the Thelebolus microsporus totivirus 1. Details of the five best matches are presented in Table 2. Accordingly, we can assume that this sequence corresponds to a partial genomic dsRNA for a novel mycovirus belonging to the family *Totiviridae*, which has been named as Umbelopsis ramanniana virus 2 (UrV2).

The third clone carried a 4,987-nt fragment containing two open reading frames (ORF1 and ORF2) in different frames and with a 98-nt spacer in between (Figure 3B). ORF1 (from 45 to 2,582-nt; EMBL accession number: LR595925) encodes an 845-aa hypothetical protein with a 92.04-kDa predicted molecular weight. Homology searches carried out with the nucleotide and amino acid sequences of this ORF using the BLASTx and tBLASTn tool of NCBI, respectively, found no similarity with any sequences in the GenBank. ORF2 (from 2,681 to 4,945-nt; EMBL accession number: LR595926) was predicted to encode a putative, 754-aa RdRp protein with an 85.47-kDa predicted molecular weight. This protein showed the highest degree of identity with the Beihai barnacle virus 15 hypothetical protein 2. Details of the five best matches given by the BLASTp query with the corresponding amino acid sequence are presented in Table 2. As this sequence may correspond to a genomic dsRNA for a novel mycovirus, it has been named as Umbelopsis ramanniana virus 3 (UrV3).

The fourth clone carried a fragment determined to be 4,642-nt in length. It contained two open reading frames (ORF1 and ORF2): ORF1 (from 29 to 2,098-nt; EMBL accession number: LR595927) encodes a putative, 689-aa CP, while ORF2 (from 2,149 to 4,602-nt; EMBL accession number: LR595928) was predicted to encode a putative, 817-aa RdRp (Figure 3B). The predicted molecular weights of the proteins were found to be 77.12 and 93.17-kDa for the CP and the RdRp, respectively. The two genes are in different frames and a 50-nt spacer can be found between them. BLASTp homology search with the corresponding fragment sequence in the NCBI GenBank revealed a high degree of identity with the CP and RdRp of viruses in the *Totiviridae* family; best matches are presented in Table 2. As in case of the sequence of UrV1, the highest similarity was found to the Wuhan insect virus 26 and 27 for both the CP and the RdRp. We suggest that this dsRNA segment corresponds to a genomic component of a novel mycovirus in the *Totiviridae* family, and we have tentatively named it as Umbelopsis ramanniana virus 4 (UrV4).

EMBOSS Needle pairwise alignment tool at the EMBL-EBI site (https://www.ebi.ac.uk/Tools/psa/emboss_needle/) found 48.7 and 60.2% sequence identity of CP and RdRp proteins of UrV1 and Urv4, respectively. In case of the other sequences, lower level of similarity/homology was detected (13.3–21.0%) as it shown in Supplementary Table S3.

UrV1, UrV3, and UrV4 contain a spacer region between their two ORFs, which are in the same frame as ORF2 (Figure 3B). Using the HPknotter program, a putative H-type pseudoknot, which may be involved in translation re-initiation (Li et al., 2011), could be predicted for each genome (from 2,077 to 2,099-nt for UrV1, from 2,529 to 2,560-nt for UrV3 and from 2,062 to 2,081-nt for UrV4) suggesting that an ORF1-ORF2 fusion protein may be produced by a −1 ribosomal frameshift in each case.

### Phylogenetic Analysis of the *U. ramanniana* Viruses

**Figure 4:**
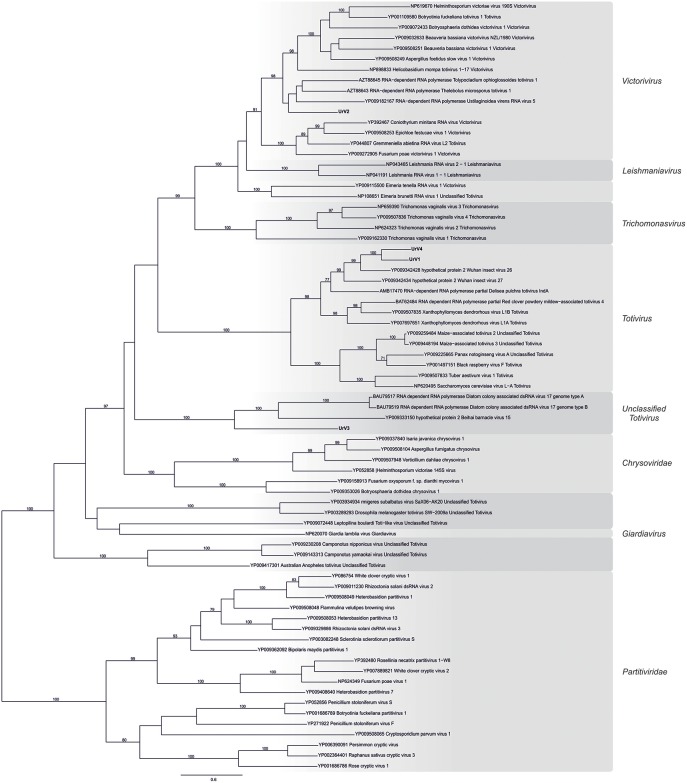
Phylogenetic analysis of UrV1, UrV2, UrV3, and UrV4 RdRps. The presented phylogenetic tree was constructed based on the RdRp amino acid sequences from representative members of the family *Totiviridae, Chrysoviridae*, and *Partitiviridae*. Results indicate that UrV1 and UrV4 are members of the genus *Totivirus*, UrV2 belongs to the genus *Victorivirus*, and UrV3 forms a clade with unclassified dsRNA viruses. The phylogenetic tree was constructed using Maximum Likelihood method using the RAxML program. Bootstrap values (%) indicated on branches were obtained with 500 replicates. The genetic distance was represented by the scale bar of 0.8 amino acid substitutions per nucleotide site. The novel *Umbelopsis* mycoviruses are indicated with bold letters.

Based on RdRp amino acid sequences from representative members of the family *Totiviridae, Chrysoviridae*, and *Partitiviridae*, a phylogenetic analysis was performed using the ML method (Figure 4). In this phylogeny, UrV1 and UrV4 is seated in the clade containing viruses from the genus *Totivirus*, while UrV2 is part of the clade representing the genus *Victorivirus*. UrV3 together with some unclassified dsRNA viruses forms well-defined and yet unclassified clade, which is related to the genus *Totivirus*. We gave the same result with alternatively performed NJ analysis (Supplementary Figure S1).

**Figure 5:**
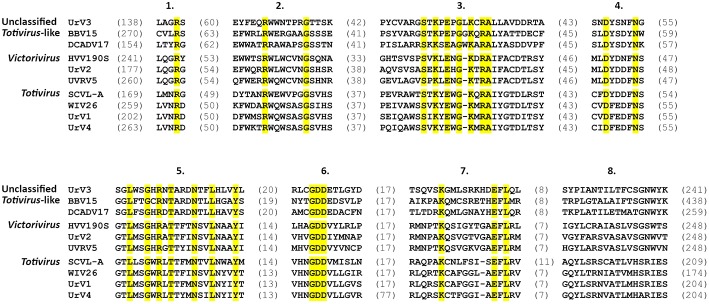
Multiple amino acid sequence alignment of the conserved RdRp motifs of the novel UrV1, UrV2, UrV3, UrV4, and other related mycoviruses. Numbers at the top indicate the eight conserved domains characteristic to the *Totiviridae* family (Bruenn, 1993). Residues found in all sequences are shaded in yellow. Sequence lengths between the motifs are indicated with the number of amino acids in parentheses. Abbreviations of the virus names and accession numbers of the aligned sequences are the following: BBV15, Beihai barnacle virus 15 (YP_009333150); DCADV17, Diatom colony associated dsRNA virus 17 genome type A (BAU79517); HVV190S, Helminthosporium victoriae virus 190S (AAB94791); UVRV5, Ustilaginoidea virens RNA virus 5 (YP_009182167); SCVL-A, Saccharomyces cerevisiae virus L-A (NP_620495); WIV26, Wuhan insect virus 26 (YP_009342428).

Besides the universally conserved GDD motif (Bruenn, 1993), multiple alignment of the viral RdRps isolated from *U. ramanniana* and those of other similar mycoviruses revealed the eight conserved domains specific to the *Totiviridae* family RdRps (Bruenn, 1993; Routhier and Bruenn, 1998; Campo et al., 2016) (Figure 5). Furthermore, group-specific sequence patterns observed within these conserved domains reinforce the result of the phylogenetic analysis that UrV1 and UrV4 belong to the genus *Totivirus*, while UrV2 and UrV3 are members of the genus *Victorivirus* and an unclassified *Totiviridae*-related group, respectively.

### Detection of Virus Particles in *U. ramanniana*

**Figure 6:**
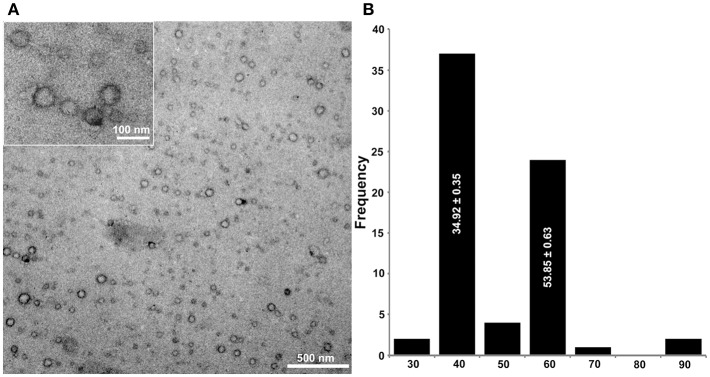
Morphology of virus particles detected in *U. ramanniana* NRRL 1296 strain **(A)** and the histogram of the particle size distribution measured by electron microscopy (*n* = 70) **(B)**. The virus particles were recovered by ultracentrifugation at 78,000 × *g* for 10 h at 4°C. Purified virus particles were negatively stained with 2% uranyl acetate in 50% ethanol for 5 min (3 times) and examined under a JEM-1400 Flash transmission electron microscope. Two different isometric virus particles could be discerned in the samples. The sizes of particles are estimated to be about 50 and 35 nm in diameter. In **(A)**, magnification in the large and small pictures are 40,000× and 60,000×, respectively.

Transmission electron microscopy revealed the presence of isometric virus particles in the purified extracts of *U. ramanniana* NRRL 1296. Figures 6A,B shows that particles with at least two different sizes (i.e., with about 50 or 35 nm in diameter) could be discerned in the samples.

### Hybridization Analysis of the dsRNA Patterns

**Figure 7:**
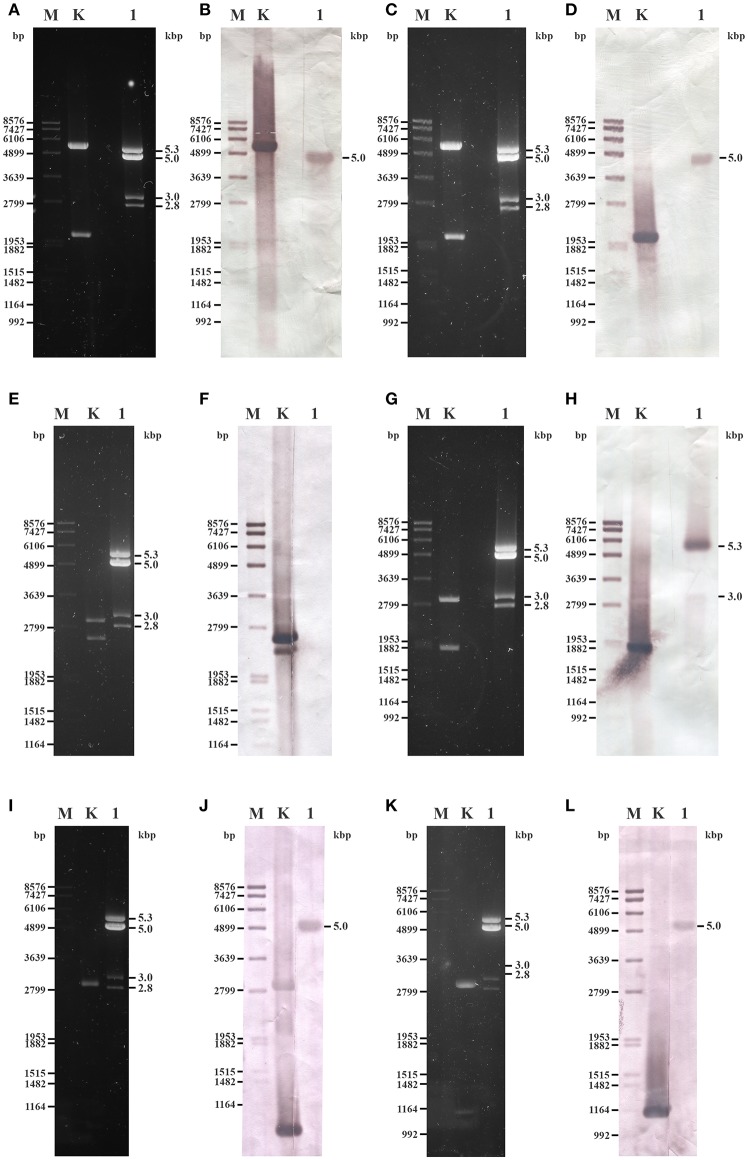
Northern blot analysis of dsRNA fragments purified from the mycovirus-harboring *Umbelopsis ramanniana* NRRL 1296 strain with probes designed by the UrV1, UrV2, UrV3, and UrV4 sequences. **(A,C,E,G,I,K)** Agarose gel electrophoresis of *U. ramanniana* NRRL 1296 dsRNA fragments. Lane M, DIG-labeled DNA Molecular Weight Marker VII (Roche); Lane K, control plasmid containing the corresponding PCR amplicon of virus genomes; Lane 1, *U. ramanniana* NRRL 1296. Right numbers indicate the sizes (kbp) of the detected dsRNA fragments. **(B,D,F,H,J,L)** Northern blot analysis of *U. ramanniana* NRRL 1296 dsRNA fragments hybridized with UrV1 CP, UrV1 RdRp, UrV2 RdRp, UrV3 RdRp, UrV4 CP, and UrV4 RdRp probes, respectively. In **(B,D)**, UrV1 CP and UrV1 RdRp probes both gave strong hybridization signal with the 5.0-kbp fragments of *U. ramanniana* NRRL 1296. In the case of the control plasmid, which contains the PCR amplicon of the UrV1 genome, the 5.5-kbp band gave strong hybridization signal with the UrV1 CP, and the 2.0-kbp band gave strong hybridization signal with the UrV1 RdRp probe. In panel **(F)**, UrV2 RdRp probe gave no hybridization signal to any dsRNA fragments, however the 2.4-kbp band of the BglII digested control plasmid, which contains the PCR amplicon of the UrV2 genome gave strong hybridization signal. In **(H)**, UrV3 RdRp probe hybridized to the largest, 5.3-kbp and the 3.0-kbp dsRNA bands of the pattern. In case of the ClaI-SpeI double digested control plasmid, which contains the PCR amplicon of the UrV3 genome, the 1.7-kbp band gave strong hybridization signal with the UrV3 RdRp probe. In **(J,L)**, UrV4 CP and UrV4 RdRp probes also gave strong hybridization signal with the 5.0-kbp fragments. In the case of the control plasmids, the ClaI-SpeI double digested control plasmid, which contains the PCR amplicon of the UrV4 CP genome, the 0.85-kbp band gave strong hybridization signal with the UrV4 CP probe. The 1.0-kbp band of the BglII digested control plasmid, which contains the PCR amplicon of the UrV4 RdRp genome gave hybridization signal with the UrV4 RdRp probe.

Probes designed for the CP and the RdRp genes of UrV1 and UrV4 hybridized to the second largest band (5.0-kbp) of the dsRNA electrophoretic pattern of *U. ramanniana* NRRL 1296 (Figures 7A–D,I–L) indicating that this band represents two molecules with highly similar sizes, which correspond to the UrV1 and UrV4 dsRNA genomes. The probe designed for the UrV2 sequence gave no signal to any dsRNA bands (Figures 7E,F), while the probe based on the sequence of the UrV3 fragment hybridized to the largest, 5.3-kbp and the 3.0-kbp dsRNA bands of the pattern (Figures 7G,H).

Although hybridization experiments to match the dsRNA electrophoretic bands with the UrV2 sequence, which was determined after amplification using the FLAC method from the total dsRNA extract, have remained unsuccessful, this fragment could be amplified from the isolated 3.0-kbp dsRNA band using both the FLAC adaptor and UrV2-specific primers (see Supplementary Table S2) as well. This result also suggests that this 3.0-kbp dsRNA band can be a mixture of dsRNA molecules.

As a band with a size similar to that of the band corresponding to the dsRNA of UrV1 and UrV4 was present in the dsRNA patterns of the other four dsRNA harboring *Umbelopsis* strains, the probes corresponding to the UrV1 CP and RdRp genes were also used to examine these dsRNA patterns. Both probes hybridized to the bands with the size of around 5.0-kbp in case of all tested *Umbelopsis* strains, except that UrV1 CP probe gave no hybridization signal with the fragments of *U. dimorpha*. It might be attributed to the presence of sequence variation in the 5.0-kbp segment of *U. dimorpha*, which will affect the binding specificity of the UrV1 CP and RdRp probes. Similar to that of UrV1, the RdRp probe of UrV4 also could hybridize to the dsRNA extracts of all four *Umbelopsis* strains (Supplementary Figure S2). At the same time, probes corresponding to the RdRp sequences of UrV2 and UrV3 did not give any signal with the dsRNAs of these strains (Supplementary Figure S2). Based on this result, we can suggest that the detected bands in *Umbelopsis* strains also can be possible full mycovirus genomes similar to those of UrV1 and UrV4 (Figures 1A–D). The probe corresponding to the UrV1 CP did not give any signal for other bands of these strains, while the probe of the UrV1 RdRp hybridized also to the smaller, 4.0-kbp dsRNA fragment of *U. angularis* suggesting that this fragment may also contain an RdRp sequence.

## Discussion

In this study, we screened for the presence of dsRNA molecules in a collection of 24 *Umbelopsis* isolates representing 11 species and found dsRNA elements in strains of five different species. Moreover, discovery of four independent viral genomes and genome fragments corresponding to four novel mycoviruses in *U. ramanniana* was also reported.

We found dsRNA elements in the 46 and 21% of the tested *Umbelopsis* species and strains, respectively. This prevalence is much higher frequencies than that observed previously testing 121 *Mucor* isolates, which represented 17 species (i.e., 29 and 4%, respectively) (Vágvölgyi et al., 1998). In another study, 19% of the tested *Rhizopus* isolates harbored dsRNAs and these isolates represented three of the four involved species (Papp et al., 2001).

The dsRNA pattern of the *U. ramanniana* NRRL 1296 was reported earlier (Vágvölgyi et al., 1993, 1998). Vágvölgyi et al. (1993) originally detected a pattern consisting of four dsRNA molecules but later, they also reported the presence of a fifth, 2.5-kbp dsRNA fragment (Vágvölgyi et al., 1998). In our study, we could reconstruct only the earlier reported pattern with the same four bands. Maybe that fifth fragment would represent a defective dsRNA element, which can frequently be present in dsRNA mycoviruses (Son et al., 2015).

Efforts to determine the dsRNA molecules of this fungus resulted in four sequences, among which three encode a CP (or potential CP) and an RdRp protein and the one corresponds to a partial RdRp coding gene. Considering that the four sequences represent different novel dsRNA viruses, they were named as UrV1, UrV2, UrV3, and UrV4. All four dsRNAs found to be related to viral sequences belonging to the *Totiviridae* family. Isometric virus particles with different sizes were also detected in *U. ramanniana* NRRL 1296 indicating that this strain harbor multiple viruses. Mixed infections have also been found in other fungi, such as in case of the entomopathogenic *Beauveria bassiana* (Herrero et al., 2012).

The phylogeny inferred from the RdRp sequences placed UrV1 and UrV4 into the genus *Totivirus* together with Wuhan insect virus 26 and 27 (Shi et al., 2016), which also proved to be the most similar to the UrV1 and UrV4 sequences by the BLAST search of the NCBI GenBank (Table 2 and Figure 4). Within the *Totivirus* clade, they form a sub-clade together with the Delisea pulchra totivirus IndA (KT455453; Lachnit et al., 2016). Hybridization analysis revealed that the 5.0-kbp band of the dsRNA pattern obtained from *U. ramanniana* NRRL 1296 corresponds to the genomes of UrV1 and UrV4. This size agrees with the typical genome sizes of the totiviruses (King et al., 2011; Ghabrial et al., 2015). A band with a similar size was found in all tested strains. Moreover, the probes, which correspond to the CP and RdRp sequences of UrV1 and UrV4, could hybridize to them indicating that they can represent similar mycovirus genomes possibly belonging to the genus *Totivirus*. In case of *U. versiformis*, both UrV1 probes gave a hybridization signal as strong as those given with the corresponding dsRNA molecule of *U. ramanniana* NRRL 1296 suggesting that this fungus may harbor a highly similar dsRNA/mycovirus (Figures 1A–D).

The sequence of UrV2 proved to be the most related to Thelebolus microsporus totivirus 1 and Tolypocladium ophioglossoides totivirus 1, which were recently identified by a survey of transcriptomic datasets of Pezizomycotina fungi and determined as members of the genus *Victorivirus* (Gilbert et al., 2019). Indeed, these viruses together with Ustilaginoidea virens RNA virus 5, of which RdRp sequence is also very similar to that of UrV2, form a sub-clade within the *Victorivirus* genus in the phylogenetic tree of RdRp sequences (Figure 4). Hybridization analysis could not match the UrV2 sequence to any dsRNA bands of *U. ramanniana* NRRL 1296 but its 2,454-nt fragment could be amplified from the isolated 3.0-kbp dsRNA band of the same pattern. These results suggest that UrV2 can represent a partial genome and/or a defective sequence, which is present in a very low amount in the fungal cells.

In case of UrV3, the RdRp probe hybridized to the largest fragment of the dsRNA pattern of *U. ramanniana*. It also gave a hybridization signal to one of the smaller bands, which may indicate that the latter could be a defective dsRNA originated from the genome of UrV3 (Figures 7G–H). The UrV3 sequence had the largest degree of identity with Beihai barnacle virus 15 identified by the analysis of invertebrate transcriptomes as a Toti-Chryso-like virus (Shi et al., 2016) and the RdRps of Diatom colony associated dsRNA virus 17 genome types A and B, which were discovered as not assigned dsRNA viruses by metagenomic methods in a diatom colony (Urayama et al., 2016). UrV3 together with the abovementioned three viruses formed a clade, which proved to be paraphyletic with the *Totivirus* and the *Chrysoviridae* clades (Figure 4). Viruses in the *Chrysoviridae* have segmented genomes typically consisting of four dsRNA segments, which are among 2.4 and 3.6-kbp in size (King et al., 2011), although unusual chrysoviruses with three or five segments have also been reported (Li et al., 2013; Ghabrial et al., 2015). *Totiviridae* contains viruses with non-segmented genomes with various sizes ranging from 4.6 to 7.0-kbp (Ghabrial et al., 2015). Despite this profound difference in their genome organizations, phylogenetic analyses have demonstrated their close relationship and similarity (King et al., 2011; Chen et al., 2016). Based on the hybridization analysis, it can be suggested that UrV3 has a non-segmented genome. The other members of its clade have also monosegmented genomes, with the sizes of 5.9-kbp for Diatom colony associated dsRNA virus 17 genome types A and B (Urayama et al., 2016) and 7.4-kbp for Beihai barnacle virus 15 (Shi et al., 2016). Considering their phylogenetic position and genome structure, it can be suggested that these viruses represent a novel, *Totivirus*-related group of dsRNA mycoviruses.

In conclusion, we detected dsRNA elements in various *Umbelopsis* species and identified four novel *Totiviridae* related viruses in *U. ramanniana*. Our results suggest that UrV1 and UrV4 are members of the genus *Totivirus*, UrV2 belongs to the genus *Victorivirus*, and UrV3 may belong to a novel group of mycoviruses in the *Totiviridae* family.

## Data Availability

The datasets generated for this study can be found in European Nucleotide Archive, LR216267-LR216269, LR595925-LR595928.

## Author Contributions

TP and IN contributed to the design and implementation of the research, analyzed the results, and drafted the manuscript. TK and BS performed the experimental work. SK performed the phylogenetic analysis. RP and TFP carried out the electron microscopic examinations. CV and GN contributed to analyze the results and helped in drafting the manuscript.

### Conflict of Interest Statement

The authors declare that the research was conducted in the absence of any commercial or financial relationships that could be construed as a potential conflict of interest.
