# Supplementary material for: Detection and Molecular Characterization of Novel dsRNA Viruses Related to the Totiviridae Family in Umbelopsis ramanniana
Source: Front Cell Infect Microbiol. 2019 Jul 11;9:249. doi: 10.3389/fcimb.2019.00249 (PMC6644447; doi:10.3389/fcimb.2019.00249)
Supplement: Supplementary file 1 [file Table_1.pdf]

Supplementary Table S1 *Umbelopsis* strains investigated in this study.

Species name	Collection number	SZMC code	Substrate/Origin	Presence of dsRNAs	Accession number of ITS sequence
Umbelopsis angularis	CBS 603.68	11252	Soil / Baarn, Netherlands	+	MH859191.1
Umbelopsis autotrophica	CBS 310.93	11276	Soil / UK	-	HQ630285
Umbelopsis dimorpha	CBS 110039	22797	Soil / New Zealand	+	AJ580917
Umbelopsis gibberispora	CBS 109328	23390	*Fagus crenata* / Japan	+	AJ495442
Umbelopsis longicollis	CBS 209.32	11208	Sandy loam / Victoria, Australia	-	HQ630287
Umbelopsis isabellina	FSU 909	11324	-	-	-
Umbelopsis isabellina	FSU 910	11323	-	-	HQ630335
Umbelopsis isabellina	FSU 922	11335	-	-	-
Umbelopsis isabellina	FSU 923	11319	-	-	-
Umbelopsis isabellina	FSU 924	11325	-	-	-
Umbelopsis isabellina	FSU 925	11290	-	-	-
Umbelopsis isabellina	FSU 926	11291	-	-	-
Umbelopsis isabellina	FSU 954	11322	-	-	-
Umbelopsis isabellina	FSU 1716	11292	-	-	-
Umbelopsis isabellina	FSU 2831	11293	-	-	-
Umbelopsis isabellina	FSU 2892	11321	-	-	-
Umbelopsis isabellina	NRRL 1757	11076	Soil / Wisconsin, USA	-	HQ630284
Umbelopsis ovata	CBS 499.82	22674	*Isopogon ceratophyllus* / Victoria, Australia	-	AJ495429
Umbelopsis ramanniana	NRRL 1296	11078	- / Wisconsin, USA	+	HQ630289
Umbelopsis swartii	CBS 868.85	23388	Soil under *Eucalyptus regnans* / Victoria, Australia	-	AJ495444, MH861920.1
Umbelopsis versiformis	CBS 150.81	23387	Root (*Quercus borealis*) / Virginia	-	AJ495434, MH861311.1
Umbelopsis versiformis	CBS 473.74	21866	Poor quality soil / Victoria, Australia	+	AJ495433
Umbelopsis vinacea	CBS 222.29	11234	- / USSR	-	HQ630286
Umbelopsis vinacea	FSU 2701	11316	-	-	-

Mortierella polycephala	CBS 456.66	11355	Dung of wood mouse / Ukraine	-	HQ630335

SZMC: Szeged Microbiology Collection, Hungary; CBS: Centraalbureau voor Schimmelcultures, Baarn, The Netherlands; NRRL: Agricultural Research Service Culture Collection, USA; FSU: Friedrich Schiller University, Jena, Germany.
