# Supplementary material for: Detection and Molecular Characterization of Novel dsRNA Viruses Related to the Totiviridae Family in Umbelopsis ramanniana
Source: Front Cell Infect Microbiol. 2019 Jul 11;9:249. doi: 10.3389/fcimb.2019.00249 (PMC6644447; doi:10.3389/fcimb.2019.00249)
Supplement: Supplementary file 2 [file Table_2.pdf]

Supplementary Table 2 List of primers applying for amplification of cDNAs and hybridization probes.

Primer*	Sequence (5' - 3')	Application	Reference
PC3-T7 loop	p- GGATCCCGGGAATTCGGTAATACGACTC ACTATATTTTTATAGTGAGTCGTATTA-OH	self-priming anchor primer for cDNA synthesis	Potgieter et al., 2009
PC2	p-CCGAATTCCCGGGATCC	Amplification of cDNA	Potgieter et al., 2009
UrV1CP F	CTTGACCAGGCTTATGATGACC	Amplification of probe for UrV1 CP	In this study
UrV1CP R	AAATCGGCTAGAACGACATTCAG		
UrV1RdRp F	GTTGCCGACTGAATTGTTTGAG	Amplification of probe for UrV1 RdRp	In this study
UrV1RdRp R	CCACCTTGACATCCAGAAATCC		
UrV2RdRp F	AGTGCTATACTATACGCTTCGGG	Amplification of probe for UrV2 RdRp	In this study
UrV2RdRp R	TCGACTTTAAGCTCCTCATCCA		
UrV3RdRp F	TTCAACGGGTTAAATTCACTCAGG	Amplification of probe for UrV3 RdRp	In this study
UrV3RdRp R	CATCATACCCAAGTGTTTCATCGT		
UrV4CP F	CAAAGACAGCATCAAGACTAACCT	Amplification of probe for UrV4 CP	In this study
UrV4CP R	CCAGTAGACAATTTCGCCTTGAG		
UrV4RdRp F	TCAAATATTCTCACCGTGTAGCAG	Amplification of probe for UrV4 RdRp	In this study
UrV4RdRp R	AATACGAATCCTGTTAGAATCGCC		
UrV2RdRp F	AGTGCTATACTATACGCTTCGGG	Specific amplification of partial UrV2 fragment	In this study
UrV2PCR R	TATAGGTAGTGTAAAGTACGCC		

*F and R indicating forward and reverse primers, respectively.
