# Supplementary material for: Detection and Molecular Characterization of Novel dsRNA Viruses Related to the Totiviridae Family in Umbelopsis ramanniana
Source: Front Cell Infect Microbiol. 2019 Jul 11;9:249. doi: 10.3389/fcimb.2019.00249 (PMC6644447; doi:10.3389/fcimb.2019.00249)
Supplement: Supplementary file 3 [file Table_3.pdf]

Supplementary Table S3 Sequence identities of proteins encoded by ORF1 and ORF2 of the virus genomes identified in *U. ramanniana* NRRL 1296.

**Virus genomes**	**sequence identity (%)**	
	**ORF1**	**ORF2**
UrV1 – UrV2	-	18.9
UrV1 – UrV3	13.3	19.1
UrV1 – UrV4	48.7	60.2
UrV2 – UrV3	-	21.0
UrV2 – UrV4	-	19.0
UrV3 – UrV4	14.8	17.5

UrV1: Umbelopsis ramanniana virus 1; UrV2: Umbelopsis ramanniana virus 2; UrV3: Umbelopsis ramanniana virus 3; UrV4: Umbelopsis ramanniana virus 4; ORF1: coat protein in UrV1 and UrV4, hypothetical protein in UrV3; ORF2: RNA dependent RNA polymerase.
