# Supplementary material for: Detection and Molecular Characterization of Novel dsRNA Viruses Related to the Totiviridae Family in Umbelopsis ramanniana
Source: Front Cell Infect Microbiol. 2019 Jul 11;9:249. doi: 10.3389/fcimb.2019.00249 (PMC6644447; doi:10.3389/fcimb.2019.00249)

Supplementary Figure S1 Neighbor Joining tree constructed using the RdRp amino acid sequences of the UrV1, UrV2, UrV3 and UrV4 and representative members of the family *Totiviridae*, *Chrysoviridae* and *Partitiviridae*. Bootstrap values (%) indicated on branches were obtained with 500 replicates.

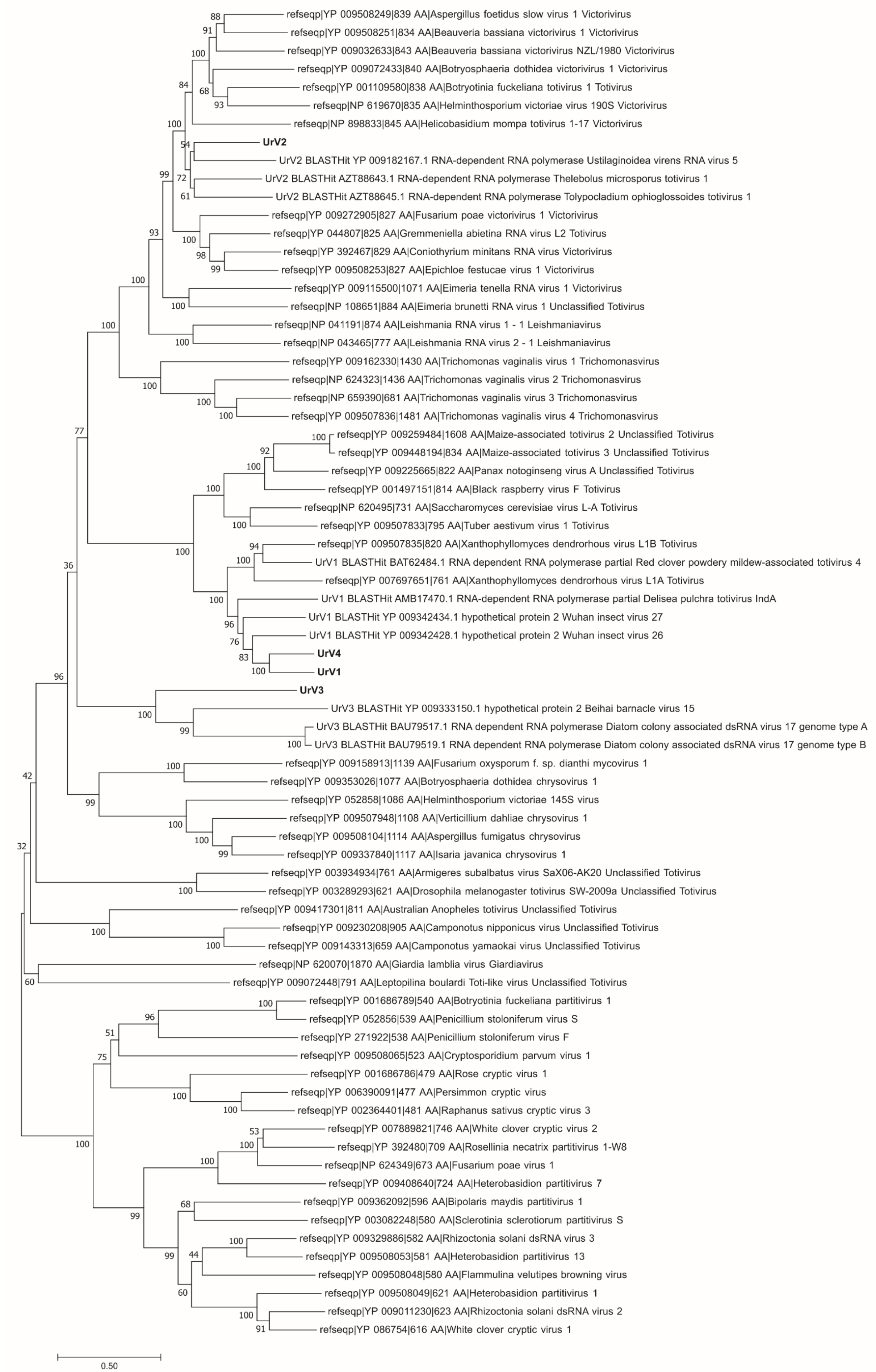

Supplement: Supplementary file 4 [file Presentation_1.pdf]
