# Supplementary material for: Detection and Molecular Characterization of Novel dsRNA Viruses Related to the Totiviridae Family in Umbelopsis ramanniana
Source: Front Cell Infect Microbiol. 2019 Jul 11;9:249. doi: 10.3389/fcimb.2019.00249 (PMC6644447; doi:10.3389/fcimb.2019.00249)
Supplement: Supplementary file 5 [file Presentation_2.pdf]

Supplementary Figure S2 Dot-blot hybridization of dsRNAs purified from the different mycovirus-harboring *Umbelopsis* strains with probes designed by the UrV2, UrV3 and UrV4 sequences.

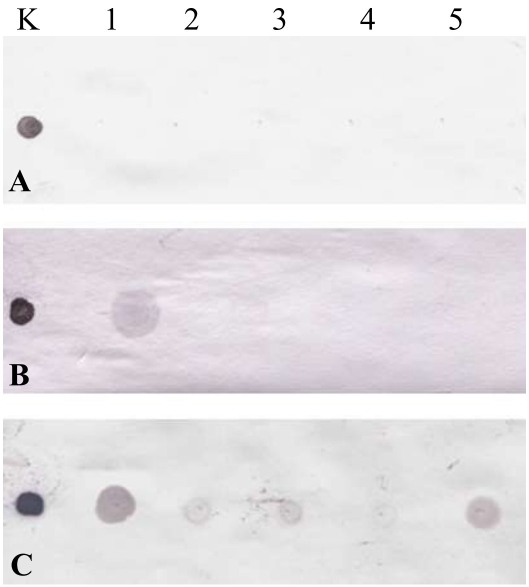

(A), **(B)**, and **(C),** Dot-blot analysis of dsRNA fragments hybridized with UrV2 RdRp, UrV3 RdRp and UrV4 RdRp probes, respectively. Lane K, control plasmids, which contains the PCR amplicon of the corresponding UrV RdRp probes, Lane 1, *U. ramanniana* NRRL 1296, Lane 2, *U. gibberispora* CBS 109328, Lane 3, *U. angularis* CBS 603.68, Lane 4, *U. dimorpha* CBS 110039, Lane 5, *U. versiformis* CBS 473.74.
