# Supplementary material for: Detection and Molecular Characterization of Novel dsRNA Viruses Related to the Totiviridae Family in Umbelopsis ramanniana
Source: Front Cell Infect Microbiol. 2019 Jul 11;9:249. doi: 10.3389/fcimb.2019.00249 (PMC6644447; doi:10.3389/fcimb.2019.00249)
Supplement: Supplementary file 6 [file Data_Sheet_1.docx]

**Supplementary Data S1** Nucleic and amino acid sequences determined in the study.

>UrV1_full nucleic acid sequence

TGAATAAAAGCAACACCCTATTAATCCCCATGGCGAAATTATTCATCAAAGACTCACTCGATGTTGGCTTCAGTGTAGGACGTGATCCAATCATTGAGAACGGCCGCTTCACAGTGGTCAACAAATCGACAGTGGATGTAAAGAAAGGAGAGAGATTCTACCACTCTGATATAGAGCTAACCGCTGATTTCATGAGTGCTGGAAGGCATAAAGGCTGTGACAGAGTCGGCGTAAACACTGACTTCTCAGGCTTCAATAAGAAGTTCATAACTGAACAGGGAGTTTATGACAGTTATGCTGCCTTGGAAGAATTTTCGAGAGTGAGCCCTGAAAAAGGGAATGTCAAACCTGACACTTATTCCGTTCTAACTAGGTTCACGACTTCTGATTCTCACGATTCGTTCTTATACAATATGCTGGTTTCATGGCTGAAAGCTAAAATGTACAAGGACTCCAACGGCGAGGGTCTGAAGCTGAAGGTGACGACCTCAGCTTATAATGACTCGCATGTGGTTGTTGCCCTTGACCAGGCTTATGATGACCATACTTACGAAATCGACATGGATGAACCGGTAACAATGGAATTACTTGAAACAGCGAACTGGCATCTAAGGACCAGAGACAACTATTGGAGTAGGCCTTTTGTCTTGGTTTACAATGGCTCTTCTGTTTCGCAAGAACTGTTCTACTTGATTCACACTTTAGGAAGGAATAAAGTGTCAGCCTTGAACTTCGACGTTGAGATCAAAGGCATACCAGGAGGTGACCTCCTCTGCGATCCAATAAACGGAAGAGATCATATGGACATGAACCTTGGGGCTGTTGACTGGACAGACCACCATAAGATGTGGTCGTGGATACTTGACTACGTTAAACTGAATCGAGTAGAACAGTCTTTCGCAGCCGCGTTTGAGACACTGGGTGCAATGGCATACCACCCGGCACCTAGTTCAATGGAAGCGTGCCTGTGGCAACATGCGAAGCTGAATGTCGTTCTAGCCGATTTTTCACCGACTAGGGGCAGGATCAGATTGAACCTAGACGGTGAGGCTTTCAAGCCACATTCGATTGCAGATGAATTCCTAATCAGCGAGATAGAATCGCCAGGCCAATTCTTAGCTGCATCAGCCCTGTGCAACTACTATATGTGGTACGGACTGTACGCCCTTCTCAGGAACGAGGCGAAGACCAGACCTGACTGGCGCACCGTGTTCTCGACTATGGCTGACGAACTGGCAATCTTGTTTACGCCTGATGCCAGAGCGGCGTGTATTAGCGTTGCCACCGGGCGAGAATTCTCTACGTGCATGACGGCGAACTGTGGCATGTATGTAGACACGAGCAGGTTAGAGGTAATACACAAACTAACCAACTTGCGTAGCTTAGACGGTACCGTTAATAGTGAAGTTGAAGTAAACTGCATCCCGTCGCCGGTCTCCGGTTCTATAGTGCTTGGCACTATGAGTGGCTACTTGGAAGTGACCGATAATCTGTCGTCAACGTACGAGTTGCCCATACTGAGCGGGCAATTCCAACCTATGACCGAACGACAAATTATGCGCCTTAGCACTATATACCGTCTGTTTGGATACGACACCACGTTGCTTAATTATTATACTAAACAGCAAGTAGAAACTTGGGCACCGGCACGTGAATGTATACCTGAGCGCATCTTCTTTGGGATGGATATCGAAACGCCTAACCTATGGACGGTAGTCGACGCCAATGTACGCGAAGGCAGGAAACACGTGATACCGACTGTGACGGATTTATGCTCGGGAGAGACGGCGAAAGTGATGATCAGAAAGCCCATGCTCAAATTCACTTCATGGAAGCGTAGAACGACGACTCTAAGACCGCAGATACGTTTCGTTAATAACAAGGAACCTGTAACCTTCAAAGTGAACTCTGCCTACAGATTTAACAAGGTTAAAATGGTTGCCAGACCTATAGAAAAACCGAAGCAGGATTTTATCAAGGGCAGCACGTCGGGACCCCCAGTACACCCAGAGATCCGCCGCGTCGAAGAAACCGATGTTACGGGTCAACTGAGTGGTGTACAGGAGGCGTCAGCTGCCGATTCTGCGGAATAAACCAGACCAAAGCGTCTTTAAATCTCTATAGCAGCGAAAGTGGTTATAGAGTGCCTGCGTATCCGCCGCAGCGCCGAAGCACTTTCTATCACTGGCAGCCGAGCTTGGACGGGGCCGGTGAGAAACGGAAATACCTAGCACTCGAAAGAGATGAAGCCCCAACATCCTTTCGAGTATCTGATTTGGGCAACTACATCGTCGTGCCTTTCAAACGCGCTCAGTATGTGCTAATCGACATGCTGGACGAGGCGTACCCGGAAGGTGATTGTATGTATAACTACTACGGATCTGTGTTTAACTGTCTGATGCTACCTAGCAAAAAACGAACATACGTTTATTACAAAGTAGATCAGTTGTTAAGACCAAGGAGTAGGAATATCCTAGCAATACTTTCGAGGCACTACATGGATAATTTCAATGGTTACTATAATGATTGGTGTTCGACTGAAAACGTATTCGCGGGTCTAAGTAAAGTCAGTGAAGTGAACTTCAGGCACAGCATCGATAAGTTGGAACAACTGCCGATAGCGAAAATATCAGCGGCGCATCACATACATTTTACTGCCGCGGAGATATGGCAATGTCTGGACAGCGAACAAAGAGACAAGGCAAAACACGCGCTACGTATAACAGCACAAGCAACAACCACTATGATGGGAGGAGTGATGCTTTGGCTAGCCATGTTGCCGACTGAATTGTTTGAGCACTTTGTTAAGACAGACATCCTAGACGCCGATAGTATGGTAGAATTCGCGAAGCGTGCAAAGAAGCTATCGGTACAAGCAAAGTCGTTTCAAAATATAGTAGAGCCAGATTTGAGAACCATGTTCGAGGTTGATGTGCTTGTCAACCGCGATGTAGGACAGGTAAATTGGGACGCGGAAAAAGCAAACCGCGTCACGCCTGATTTAGTAAATCTTAACAACAAGAGAATTTACGATGCAGCTATCAAGATGTTTTCGCGTGTGGATGCAACAAAGCAAAAACCACGGAGGATGTCATGGCGGGATTTCTGGATGTCAAGGTGGCAATGGAGTGCTTCTGGGTCAGTGCACAGCCAATACGCCGAAGATCTGAAGGATTTGCCTAAAGAAAGAGAGTTGCGGAACAAATTTATACAATTGTGTCAGGCAGGTAATTATGATGCAAACCACTTTTTACAGCGACGGTCTGAAATTCAAGCGTGGTCCTCAATCAAGTACGAATGGGGTAAAATGCGCGCTATTTACGGAACCGATATCACTAGCTACGTCCTTGCGCATTATGCCTTTTACAACTGTGAAGACGTACTGCCGAATGAGTTTCCTGTTGGGTTAAAAGCGCGCCCGTCTTACGTCAGTGCCAAGGTCCAATCAGTATTAGAGAGAAAGGTACCATTATGCGTGGATTTCGAAGATTTCAACAGTGGGCATTCTAACCAAGCAATGCAAACCGTGATTCAGGCATACTACGATGTCTACTCTGCCGGTATGGATGATGACCAAAAGCGAGCAATATTGTGGACACGGGACTCTGTGGCAAGAACGCAGATCAATGATAACATGGGCACTAAGACGTCGTTCAGTACAAATGGAACGCTAATGTCAGGTTGGCGGCTAACCACGTTCATGAATTCTGTGTTGAACTACATTTACACTCAGCAGCTACTTGAGAACTGTGGCGAGCATGTGAACTCAGTACACAACGGCGATGATGTGCTACTAGGAGTGTCGAACTTCGACATCGCTAGGAGAACCGTGTATAATGCCGAGAAGTACAATATCAGGCTACAGAGAAGTAAGTGTGCTTTCGGTGGCATAGCCGAGTTCCTAAGGGTGGATAGAGTAAGAGGGGATTTTGGTCAATATTTAAGTCGCAACGTGGCTACATTGATGCACTCAAGGATCGAATCTAAGGTTGCACTAAATGTTGTGGATATAGTGGAGGCAGATGAAGAACGTTTCCGCGAGTTCGTGCGACGAGGCGGTGATGAAGCTGTGGTGTCAAGGCTGAGGCATTTAAGTTATAAACGAACGGCAAAAATATATGACACCGAACTTAGCACATTATACATGATTAAGAGCAGCCACAGAGTCGTAGGGGGCATAAGCGACTTAGATAATGCACCTGTTGATTGCATTATCGAGAAAGACAAAACAGGCAAGATATTACCGCTGCCTGATCAACTACCGGGTGTTATGGATTACGCCATAATGCTTAAGAAATCATTAGAACTTACCGTGAGTACACGAGAAGTCTACAAACGCGTATACAACGCCACACTAAATGCTGTGCAGCTGGTGAGAACCAGCGTAAAGCATACATACAACGAAAATATTCGTCAGTATGAAGTGTTCAGAGCATTGTATAAAGCACATTCCGACACTACGGATACGCCACTGTTTGGCAAAGCAATGTTGACAGGGTTTGTCTTCGATGTTCTGAACAAATCGAAGAACATGACCACGTTAATAAGGATGTTACAACAGTCAGCGGATCCGATGCGACTCTTGCGAGTCGTAGCTTGAAGCGGTGCCGGAATAGAGTGCGGCATACCCGATGTCA

UrV1_ORF1

MAKLFIKDSLDVGFSVGRDPIIENGRFTVVNKSTVDVKKGERFYHSDIELTADFMSAGRHKGCDRVGVNTDFSGFNKKFITEQGVYDSYAALEEFSRVSPEKGNVKPDTYSVLTRFTTSDSHDSFLYNMLVSWLKAKMYKDSNGEGLKLKVTTSAYNDSHVVVALDQAYDDHTYEIDMDEPVTMELLETANWHLRTRDNYWSRPFVLVYNGSSVSQELFYLIHTLGRNKVSALNFDVEIKGIPGGDLLCDPINGRDHMDMNLGAVDWTDHHKMWSWILDYVKLNRVEQSFAAAFETLGAMAYHPAPSSMEACLWQHAKLNVVLADFSPTRGRIRLNLDGEAFKPHSIADEFLISEIESPGQFLAASALCNYYMWYGLYALLRNEAKTRPDWRTVFSTMADELAILFTPDARAACISVATGREFSTCMTANCGMYVDTSRLEVIHKLTNLRSLDGTVNSEVEVNCIPSPVSGSIVLGTMSGYLEVTDNLSSTYELPILSGQFQPMTERQIMRLSTIYRLFGYDTTLLNYYTKQQVETWAPARECIPERIFFGMDIETPNLWTVVDANVREGRKHVIPTVTDLCSGETAKVMIRKPMLKFTSWKRRTTTLRPQIRFVNNKEPVTFKVNSAYRFNKVKMVARPIEKPKQDFIKGSTSGPPVHPEIRRVEETDVTGQLSGVQEASAADSAE

UrV1_ORF2

MLDEAYPEGDCMYNYYGSVFNCLMLPSKKRTYVYYKVDQLLRPRSRNILAILSRHYMDNFNGYYNDWCSTENVFAGLSKVSEVNFRHSIDKLEQLPIAKISAAHHIHFTAAEIWQCLDSEQRDKAKHALRITAQATTTMMGGVMLWLAMLPTELFEHFVKTDILDADSMVEFAKRAKKLSVQAKSFQNIVEPDLRTMFEVDVLVNRDVGQVNWDAEKANRVTPDLVNLNNKRIYDAAIKMFSRVDATKQKPRRMSWRDFWMSRWQWSASGSVHSQYAEDLKDLPKERELRNKFIQLCQAGNYDANHFLQRRSEIQAWSSIKYEWGKMRAIYGTDITSYVLAHYAFYNCEDVLPNEFPVGLKARPSYVSAKVQSVLERKVPLCVDFEDFNSGHSNQAMQTVIQAYYDVYSAGMDDDQKRAILWTRDSVARTQINDNMGTKTSFSTNGTLMSGWRLTTFMNSVLNYIYTQQLLENCGEHVNSVHNGDDVLLGVSNFDIARRTVYNAEKYNIRLQRSKCAFGGIAEFLRVDRVRGDFGQYLSRNVATLMHSRIESKVALNVVDIVEADEERFREFVRRGGDEAVVSRLRHLSYKRTAKIYDTELSTLYMIKSSHRVVGGISDLDNAPVDCIIEKDKTGKILPLPDQLPGVMDYAIMLKKSLELTVSTREVYKRVYNATLNAVQLVRTSVKHTYNENIRQYEVFRALYKAHSDTTDTPLFGKAMLTGFVFDVLNKSKNMTTLIRMLQQSADPMRLLRVVA

UrV2_full nucleic acid sequence

CGCAAAAACATCCTCACTATGATGCTGCCGTCCGTGCTGCCATTACCCTGTTACTCACGTCTTTCCCTGTCCAAGCCCCTATGTCCTCCATCGACATTATCGGCCTGGCTAGACTTGCTTTTCCGGTTGGCACTTACTCACGCAGCCCTCACTCACCTCCCACGAACTTACCCCTCAGAGCTATCCTTAAGACACCATTCCTTATGCAATATTTTCCCTTTAAATACCACCCTGCCGCAACTATGAAAACCAACGTCAGGATGTCCGACCTCCTACGTTCATTGGCTTCGCAAGGAATCCTGGCCAGATTCGAAACTATGATCTACGCACTCGCCGGCCGCGTCAGCGACGACCAGGCGTGCAGTGCTATACTATACGCTTCGGGCCTGGCTCCACACCTAGGCCCATATGCTTATGAATTTGCCTCATGCTGCGTTCTATCGCCCAAGAACGCTAAAGGCCTATCTACGGCGCTCAAAGCCCTCGGAGCTAACTCCCATCCCACTGGGGCTTTGCTGATAGAAGCCGACACCCTACAAGGACGGGGGGTTGGCTCTGTTGATTTACTACAGGAGGCCAAATATCGTTGCGACCCCTCCCTTGTCGCGGCTAGCGTCATAGACCTAGACCCCGAACTTTTACGGCTCGCTATCGACGACATTTTGGATGAGGAGCTTAAAGTCGACAAGGTTGAAATTCCGACGCCCCAGGAATTCTGGCAACGCCGCTGGCTTTGGTGTGTCAACGGCTCCCACTCGCGGGTGCTTGACCGTAGAGGGGGCCTCGACACCCGTTCCATCTTCCCTGGGGTCGATAGAGTATACCGGAGAATGTACGCGGAAGCCCAATCCGAGGAACCGCTGACTTCTTGGGATGCCCAGGTCTCGGTCAGCGCTAGCGAGAAACTCGAACATGGCAAGACCAGAGCCATCTTCGCGTGTGATACACTTTCGTATTTCGCTTTCGAGCACCTACTCTCCCCAGTAGAGAAGGCTTGGAGGGGTGTGCGCGTCGTACTGGACCCTGGTACGATGGGGCATCTCGGGGTAGCGAACAGGATAGCACGTATCCAGATCGGTGGCGTCCACGTCATGCTGGATTACGATGACTTTAATTCGCAACACTCGACCTCGTCGATGAAGGTGCTCTTCCAGGCGCTGACAGAGAGGATTGGATACCCTCCCGACCTTGCTGGAAACATATTGTCGTCCTTTGATAACATGTGGTTGTATTGCAAAGGTACGCTGGTCGGACGGGCTGAAGGTACCCTCATGAGCGGCCACCGCGCCACCACGTTCATAAACTCCGTGCTGAACGCGGCATACATTCGCCTGTCACTAGGTAAAGAGGTGTACGAACGCTACAAAGCTGTGCATGTAGGTGACGATATTTACATGAATGCTCCCACCCACTCTGACGCTGCCCTCGTCCTAACCAGAGCTCAAGCGTTAGGTTGCCGCATGAACCCCGCCAAACAGAGTGTTGGTACCGTTGGTGCTGAATTCCTCCGCATGGGCATCAGGCCTGGGGGGGCAGTGGGTTACTTTGCCCGCAGCGTAGCTTCCGCAGTCTCAGGTAACTGGGTTACCGAGGCCCGTCAGTCACCAATAGACGCTCTCAAGACGGCCATAGCCAATGTTAGAACACTATGCAACCGCAGCCGTTCTGAAGCGTTCCCCACCCTGTTAGCAGGACCTCTGTCCCGGCTAACACGGGTCCCACGCCGTCTAGTGCACGCCCTCCTAGACGGCTCTAAAGCAATTGAGGGCGCGCCGATCTTCCACTCAGACGGTATGATCAGGACGATCACGGAGGTGCCACCTCCTCCTGAACAAATGGCAGAACGCCTACCTCCCGACTGGCCACGTCACGCAACTAACGATTACGTCGTTAATTGTGCGACCGCCGTAGAACAGTTCGCTCTGAAAGAAGCTGGCAGGAGTGTTGCCTCCGCTATGTTAATCGCATCTTATAACAAGGCCTTAACAAGTGTCGCAGCACCCCCAGCCTACCCTCGCTTCAAAGCTAACCGGCCTAGGGCTCCAGTCGGTTCCCAACCCGCGTTGGCGCTCTTATACACCCGGAAGACATCCGGGGTGCTCCAAGGTTACCCACTGCTAACCCTGCTTAAAGCCCACATAAGCAGGACACTACTTAGGACTTTAGTGATCCTAGCTGGAGGGGATCCTAATGTTTCAGACCTCGACGCCGAGGCCTGGGGCCCTGACAATCGGTCCACAATTGTTCAAGGCTCTCTCAGCTATGCTGACGCGGCCGCTTTGTCAAACAAAGCTACCACTGGCGTACTTTACACTACCTATAATGTTTATATGTAGGTAAGCGTGGTCACCCACCACGTAAGCCCCTGACCTGTAAAGGTCCAGACGGTTTCTCAGGAAGCTGTATGGACGGTCACGCTCAGATATACACAGCCCCGAAAGGGGTTGTTTAGCA

UrV2_ORF1

QKHPHYDAAVRAAITLLLTSFPVQAPMSSIDIIGLARLAFPVGTYSRSPHSPPTNLPLRAILKTPFLMQYFPFKYHPAATMKTNVRMSDLLRSLASQGILARFETMIYALAGRVSDDQACSAILYASGLAPHLGPYAYEFASCCVLSPKNAKGLSTALKALGANSHPTGALLIEADTLQGRGVGSVDLLQEAKYRCDPSLVAASVIDLDPELLRLAIDDILDEELKVDKVEIPTPQEFWQRRWLWCVNGSHSRVLDRRGGLDTRSIFPGVDRVYRRMYAEAQSEEPLTSWDAQVSVSASEKLEHGKTRAIFACDTLSYFAFEHLLSPVEKAWRGVRVVLDPGTMGHLGVANRIARIQIGGVHVMLDYDDFNSQHSTSSMKVLFQALTERIGYPPDLAGNILSSFDNMWLYCKGTLVGRAEGTLMSGHRATTFINSVLNAAYIRLSLGKEVYERYKAVHVGDDIYMNAPTHSDAALVLTRAQALGCRMNPAKQSVGTVGAEFLRMGIRPGGAVGYFARSVASAVSGNWVTEARQSPIDALKTAIANVRTLCNRSRSEAFPTLLAGPLSRLTRVPRRLVHALLDGSKAIEGAPIFHSDGMIRTITEVPPPPEQMAERLPPDWPRHATNDYVVNCATAVEQFALKEAGRSVASAMLIASYNKALTSVAAPPAYPRFKANRPRAPVGSQPALALLYTRKTSGVLQGYPLLTLLKAHISRTLLRTLVILAGGDPNVSDLDAEAWGPDNRSTIVQGSLSYADAAALSNKATTGVLYTTYNVYM

UrV3_full nucleic acid sequence

GAGCTTCGTTAAATTTTGTACATATCTACTCAACTGACCCAACAATGCTTTCTTCAAATACTTCAATCAACTACTCAACTCAGAACATCCCAGGAGGCCTAGGCGCGAATGAGTTCTGCCCTCCTGTAGGTGCTGAGCGGCAGTTGGCCATTTCGGAGGCGGTCCGAGCGGACCACGTTAGGATGCGGCACCAGATCAACTGGCGCTCCAGGGGAGATGACTTCTTCACGTTGGAACAGGAGGTGGGAGAAGACTATAGGTTTGATTACCCTGCAGCTGTCCGGTTCAAGACAGGATCTGCTTGGGATACGCGCACGCCCATCCTGCACCCAGCCGTGGCTGGCCTAGACTATACCGAGGCTGGATTTGACGAGGCCTACACGCGCGCCTTGGCCAATCTGGACATTCTAATGACAGGACGCAGGGAGGAGAACACCATGCTAACACGCAGTATGTTGCGCAGGTATGCAAAAGCCAGCTACAATCCATACCGTGTACTGTTCCGTGGGGCAGCATTGTGGGCAGAATTAGTCATCCGTGACTTGCAGGGGGTGAATGCCGTTGATTGGACAGTCTCAGGCGGAATTGAGCAGGCACGCCACATAGGCACCATTAGTGACTACGCTGCGGCCTGCAAGGCCATGAACGCCCGGCCAGGAGATGTATTGTATGTTAGGTGCGATAACAATGAGGAGATGTTCATGGTGGACGTCATGCAGGCATTGGCTAGCGACACATTCCCCGTGGCGTCGAGGAACACAGTCAAGCTGCTATGGCCAAGCCTCGTACGTCCCAGGGTCATGTACAATGCGCTGGTACCTGTCACCAAGCCTGGCTCGTCACTTTGCGCTGGCGACGTCTTCGACACCATGGTGAGGTACTCGGCTATCCATGACTGCCACGACTTGTGGCGGGATGCTTTGATGACTGTGCAGTCCCTCCTGTGCCGACCGAGAGGCGCGGGGGTTCTTGCCGGTGGGCGTGCCCTCGAAATAGCATTGCCCCAATCGGACCTGGCATGTTGCATCTTGGGGCCTCTGTATGCCGGGCTTACACCAGAGGGGATGAAGACTGAGGACTTCTTGGCTCCTGATCCCAAAACGTACTTGTATGGTAGCGCTGTGCGTGGCACCTTCGCCACCGCGGCATATTATGAGGAAGTCAAGAAGGTTACTGATGCCCATCCCGTTCTTCTTGAAACTGGGATTGTGTCCCATCGGCACTTCAGGATTCTTTCAGATCCCAACATGGCCAGGCAGTTCTGGGAAAAGAAGGTGGCAGTCACTGCGTCAGATGCAGGGTGGAAGTGTGTGACGCTTGGCATGACTGGCTTCTGTCTTGAAATGACGGCCGCCACGGTGAAGGAGGCCTTCAATGCCGCGCGTGTGCCTTGGTGGACCAATGTGCTGCCACACTTGGCTAACGGTGGTTATGATTTCCTGAAGAGTTGGGTGAGTCCCGCCACACTCACTAAACTGCCACATGCTGGAGTATGGTACCCCTTTCATTGCGTTGGAGTAGTCACGAGCGAGCAGTTGGCTGCTGCTGTGCGGTGGACTGGTGCAAAAGTGCAGTATATGGTGGACACAGCCCAGTTCCACCGCTCCAGAGTGTTCGTGAATACTGGCAGCCACAACCGGTTTCTGCCGCCGTTAGCACCGGACGTTGCAATTAAAGGAGGGTATGCCCGGGCAGCCGTCATGTTCGGAAGGGACATCGGCAGAGGGGCGGAGTTATTGAAGAAGCTCGGGCGCTGTGATGTCGAAGTAACACACATGTTCAATGCAGAAAGTGGGGAGTGGCAGTTCTTCGGACATGAGTTGCCAGCAGCCCCCAGTCCAGGGAGCCTCATAATAGATACACCGAGCGGGCGCCAGGCTGCCAGAGAGGCTGCTGGCCGTGATAGGGAATTGGGCGTGGCACGTGAGGACGCCCCGCTTGACGAGGAAGTCCTGCGGGCCGCGGCCATTGCGAGGCCCTTCGGGCTGTCTTGGGAGGCAGATGTCCTGGCTGAGCCGGCAACCCGGGCACTCACGGCTGATAAGCAGGCTGTAGCCAATGGTCTCATCTCAGAGCTTAGTGGTGTGCAGCTGGCCAACTGGCTCTCAGACTCTCAGCCGACCACCAAGAAGATGGAGGTTGCTGGGGCTCTGTACACACTAGCATCCCGGGCAGCATTGTTTGCCAAGCCGGACGATTTTTACCAGCAGAAGCTATACAAGTTGGTGGATGAGTGCCGGGGGCAGATCGCGCAGTTGCAGCGAGAACTTAATCACCAAGAGAGACGGGCGGCAAGCACCCCTGTGACCATAGCAGAGGCTGCAGAGACGATCACTGGACCACCCGCTCAGACATTTAGTGAGGCACTCAAAGCCGGGGTTGCCGTTGCGTCCACTGATACGGGGGACATTAGGGCAGCACCAGATGACGCGGAGGGTGAGACACAAACTCCGGAGGATTTTGGCAACGGTGCATCCGGCCCAAGCTCACTCCCCCAGGAGGCACCTGTAGGAAATGTGGAGTCAGCACCGGTGTCAGTGGAGAGTATTGGATTTCTTCCCCCGACCGATTCGCGCAACTAAGCGCGAAGACAGACACCCTCCCAGCCTGTTGTGGGGCTAGTGTGAGGGTGGAATGTGGGGCAGGGGTGGTGGCCATTGAGGATGCCATTCTGCTGGGGATGGCTCCAGACCCGCAGCCGGTGAGAGAAGATGTTGTGGAAGTACAGAATGTTGAGTGGACCAGGGAGGCGCTGAAGGGAGCATATGAGAGGCCCCCCCGACAGTACAGTGATATGATGAAAGTGTCGATGCTCACAGTCTATGACATTGGATTGTTCGATGAGCCACTTGTGCGTGAGGCTGCGGAGGCGCAGAGAGGACAGTTGGAGTATGCGGTAGCGGCCCTGTGCCTCTGGCTGACCACTGATGTTGCCAAATACTTGTGTGCAGAACTCCCAGTACACAGAGTCCCGTTGTCCAAGTGGCCTGGCAGTGTTAAGGCATTCGCAAATGACGCTCGTAGACTAGGACAGGTATTTGGGCGCGGGCCCCAGGAAGTGGCTATGGCCTTCAGACTGAGGAGGTTGGTCAGCCTGGCCGGCAGGTCTACGGCAGATGCGGACTGGGAAAAGGAAGTAGCAGAGAGGACCCAGCTGACAACGGCAAAACGGGCGTTTGCCGACGGAGAAGTGTCCTCTGCCGCATACCGCTTAATCCGGGACAAAGTGCTGCACCGCATAGCTGTGCAGGTTGTTAATAGCCTCAAGAAGTCTGGAGGGTCGTTTGACGAATACTTTGAGCAGCGTTGGTGGAACACCCCGCGTGGCACCACGTCGAAGGGAGGAGACGTCAAGCGCCAACTCAAGAACGCCGACAAGCACCTGGACCTGCAGATGCGTCCAATCAAACCCACAGTGATGGAATTGTATTCCAAACCAGGCCTCCTCCAAGACTTGCGCGGGCTGCCATACTGTGTGGCTAGAGGTTCAACGAAGCCGGAACCAGGACTCAAGTGCAGAGCACTGCTGGCCGTGGATGATAGGACAGCCATTGTGGCAGGATACGCCTCATCCGGCATAGAGACGACTACCAAGGAGGGGGGTATGGTGCTCCGTCAAGACCCAGCCGATGTGGCAGAATGGGTGTCATTCGACCTCGGGCCGGGTGTCTGGAGGGTCAGCAACGACTATAGTAATTTCAACGGGTTAAATTCACTCAGGTCCATGCAACTGGTGGACCTGCATCTTGCCCAGGAATGGCGTAGAGTACCGGAGAGATGGGCGGAAGAGAAAGCACTGGCAAGTGAGTGGGTGGCCGCGTCATACCTCAATCCATATATGAAGACACCACTTGGAGAGACCAGGGTTGTGTCTGGCCTCTGGTCAGGGCACAGGAACACTGCGCGTGACAACACCTTCTTGCACTTAGTGTACCTCGAGTGCATCAAGTCGGTTATGCGTGCCCTCTTTGGGCAGCATGCCAAGCATGGTAAAGTGCGACTGTGTGGAGACGATGAAACACTTGGGTATGATGAGTGGTGTGCTGCCGTGCTGCATACAGTTGTGGCTGACGAGTTGGGATTCACTTCACAGGTGAGCAAGGGAATGCTCAGCCGGAAACATGACGAATTCTTGCAACTGCTGCGTCAGCCTGGCAAGGTGCCATCATACCCGATAGCCAACACCATCCTAACCTTTTGCTCCGGCAATTGGTACAAAGACCCTGTCCGTGATCTGAACACTACGGTGGCTGATGTCAGCGATCACCTGTGGGATTTGGTGCTGGGCGGAGTTGACCCGGACGTGTGCCAGCGCCTGGGCGTCTATGTGCTTGATTACTTGATGCAGGTCAAACGCAGTGATGGGTCACTGTTCCCGCTGGAGTGGTGGGACTTCCGTGGCTCAGGCATCCCGGGAGGTCATCCTCTGTGGGGTGGATTTGAAACCCCGGCCCCTCCACAGATCAAAGTCAAATTGCCCACCATCAAGCTCCCTATGGCTGCCACCCAGGACAGTGTGAAACGGGAATGGCCTGTTTGGGAGCGGCTAGAGAAGCACAGGTTGGCAGAAACCATGAATGAGCGGGCTTGGTCCTCTTACCGCGTGGTCGCAAAGCACTGGTTACAGGAGGAGTACGACAAGGCGGCCCAGGAGGAGTGGCCTGCAAGACGAGACTGCGTTAAAGTGCACATTCCAGTGGTCCGCCGTGAGGTGCCAACCAACAGGTGGCGAGCCATTGGCGACCGCAACCGTGCTAGATCGGCCCGTGCCGTGGCAGTGAAGTGCGGATTCCCACCTGAGTTACTGGGAAGTGATGACATGTGGAAGGCCATGGCTTGGCTCTCGCCACGTGACCGCTCAAACATGTACGCTGGGTTGGCTGAGCGCCAGTCCACCACCAAAGGCTGGCGCTGGGAGATGCCACCGCTCTTGCGCACTGACTAACGGTCAGTGTGACTTACATTAAATGGGAAACCATTTTCCCCC

UrV3_ORF1

MLSSNTSINYSTQNIPGGLGANEFCPPVGAERQLAISEAVRADHVRMRHQINWRSRGDDFFTLEQEVGEDYRFDYPAAVRFKTGSAWDTRTPILHPAVAGLDYTEAGFDEAYTRALANLDILMTGRREENTMLTRSMLRRYAKASYNPYRVLFRGAALWAELVIRDLQGVNAVDWTVSGGIEQARHIGTISDYAAACKAMNARPGDVLYVRCDNNEEMFMVDVMQALASDTFPVASRNTVKLLWPSLVRPRVMYNALVPVTKPGSSLCAGDVFDTMVRYSAIHDCHDLWRDALMTVQSLLCRPRGAGVLAGGRALEIALPQSDLACCILGPLYAGLTPEGMKTEDFLAPDPKTYLYGSAVRGTFATAAYYEEVKKVTDAHPVLLETGIVSHRHFRILSDPNMARQFWEKKVAVTASDAGWKCVTLGMTGFCLEMTAATVKEAFNAARVPWWTNVLPHLANGGYDFLKSWVSPATLTKLPHAGVWYPFHCVGVVTSEQLAAAVRWTGAKVQYMVDTAQFHRSRVFVNTGSHNRFLPPLAPDVAIKGGYARAAVMFGRDIGRGAELLKKLGRCDVEVTHMFNAESGEWQFFGHELPAAPSPGSLIIDTPSGRQAAREAAGRDRELGVAREDAPLDEEVLRAAAIARPFGLSWEADVLAEPATRALTADKQAVANGLISELSGVQLANWLSDSQPTTKKMEVAGALYTLASRAALFAKPDDFYQQKLYKLVDECRGQIAQLQRELNHQERRAASTPVTIAEAAETITGPPAQTFSEALKAGVAVASTDTGDIRAAPDDAEGETQTPEDFGNGASGPSSLPQEAPVGNVESAPVSVESIGFLPPTDSRN

UrV3_ORF2

MAPDPQPVREDVVEVQNVEWTREALKGAYERPPRQYSDMMKVSMLTVYDIGLFDEPLVREAAEAQRGQLEYAVAALCLWLTTDVAKYLCAELPVHRVPLSKWPGSVKAFANDARRLGQVFGRGPQEVAMAFRLRRLVSLAGRSTADADWEKEVAERTQLTTAKRAFADGEVSSAAYRLIRDKVLHRIAVQVVNSLKKSGGSFDEYFEQRWWNTPRGTTSKGGDVKRQLKNADKHLDLQMRPIKPTVMELYSKPGLLQDLRGLPYCVARGSTKPEPGLKCRALLAVDDRTAIVAGYASSGIETTTKEGGMVLRQDPADVAEWVSFDLGPGVWRVSNDYSNFNGLNSLRSMQLVDLHLAQEWRRVPERWAEEKALASEWVAASYLNPYMKTPLGETRVVSGLWSGHRNTARDNTFLHLVYLECIKSVMRALFGQHAKHGKVRLCGDDETLGYDEWCAAVLHTVVADELGFTSQVSKGMLSRKHDEFLQLLRQPGKVPSYPIANTILTFCSGNWYKDPVRDLNTTVADVSDHLWDLVLGGVDPDVCQRLGVYVLDYLMQVKRSDGSLFPLEWWDFRGSGIPGGHPLWGGFETPAPPQIKVKLPTIKLPMAATQDSVKREWPVWERLEKHRLAETMNERAWSSYRVVAKHWLQEEYDKAAQEEWPARRDCVKVHIPVVRREVPTNRWRAIGDRNRARSARAVAVKCGFPPELLGSDDMWKAMAWLSPRDRSNMYAGLAERQSTTKGWRWEMPPLLRTD

UrV4_full nucleic acid sequence

GAATAAAACACATAGGCGTATAATCCCCATGGCTAAACTATTCATCAAAGACAGCATCAAGACTAACCTCTTCTCCACTAGGGATCCCATCTTCCCAGGGGCCAGGTTTACCGTAGCAAACAACTCAACAATCGTAGTAAGACACCAAGAAAGGCAGTTTCTATCTGGACTTTCTCTTACTGCTGATTTCCAGGCTGCTGGCAGGCTCAAAAAAGTCATAAATGCTGCCGTGCAGACTAATTATTCTGGCTTCAACAAGAAGTATATTAACGAGGCGGGTGTCTATGACGGGAGTCTAGCACTGGACGAGTTCGCGAAAAGCGGCGCTGACAGAGGACTAGTAAGGCCCGAGACTTACAGCATGCTGACCAAGTATCCTCAAGCTGACTCACATGAATCGTTTATATACAATATGTTGGTGTCATATCTCAAGGCGAAATTGTCTACTGGCAATCTTACAGAAGATGAGGACTTGAAAGTAGAAACGTCGCCTTATGTAGACTCTCATTGCGTAGTACCTCTTGACCAGGCCTATGAAGATTTCACCTATGAAATCGAGCTGGGATCGCCCGTCGACGGGCGTCTCGCTATGCAAGGTCAGTTTATGGTTCGAAACAAAGATAACTATTGGAGCAAGCCTTATGTGTTACACTACAACGGAACGTCTACTAAAGCTGAGTCATTTTACTTGCTTCATGCAATGGGTAGGAACGTGGTGTCAGAGCTCAACTTTGATTTTCCGATTAAGGGTGCTGACACAGCACACATGCTTATAGATCCAGTTAACGGCAGAGAATTTGCAACCATAGATTCTTCAGAAATTGACTGGACTGACCATGAATCTATGTGGTTGTGGATCCTGGACTACGTCCAACTTAACCGTCTCGAACAAGCATTCGCAGCAGCGTTTGAGACCCTAGGAGCCCTCGCGTTTCAACCTCTACCCCCAACGGCTGAAGCCTGTCAATGGCAACAAGCCCAGCTAACTCTAACTCTTGCTAGATTCTCGCCTACCAGAGCGCGCCTAAGAAACAACCTAGCTGGAGAACCCTATAAAGTAGATTCTCTAGCTGATGAATTTCTGATCTCTGAGACGGCCTCTGCAAGTCAGTTCCTAGGTGCATCAGCTATCTGTAACTACTATATGTGGTACGGCCTGTATACAATACTGCAGAACGAGGCAAGCGAAATCGAACAGTGGCAGAATGTATACACATCTATACACGGAGTATTGCAGAACCTCTACTCGCCTGCAATGAGGGCTATGTGTATAAGCGTCGCCACAGGCAAAGAATTTGCGACATGCATGACTGATAACTGTGCTATGTTCATAGACATGTCTCGATTGGAAGTGATGCCTAAGATCACTAATATAAAGACCCTAGACGCATCAGTTCCGGCAGAAATAGTCGTTGATCACATACCAGCACCCGTGTCGGGAGCTATAGTGTTGGGTACTTTCACTGACGAGTATGATACCACAGCACACTTGTCCGCCGTATTCAGCTTACCAACTAGTGACGACCCGTACGCTAGATTCTCAGAGACGGAGCTACTTAAGATAGCAACCGTCTATAGGCTTTTTGGTTATGACACGGAACTGGTCGACGTCATAACGGATATTCCAATGGCCTTGTGGGCGGCGAACAGAGAGTGCATACCAGATCCGAGTAAGCTCCTCGCCTACAGGAGATTGAGGAGAGACTGGATAATATCGGATATAAGCCCAAGAGACGGCAGGAAAGAAGTTATTGACAGCATACAAACTCTAACATCAGGCAAACCCGCTACAGTTACCATACAGCAGCCGACTATCAGTTTCACAAGCTGGCGGCAGAGAGTGAGGACTCTTAAACCACAGGTCATTGTGGCAAAAAAGAACAAGAAGAAAGAGATACGCTTTAAAGTTAACGCGTCAGTAAGAATGTTAGATACCACGTTGATGGCTAGGCCTATAGCAGCTGTATCTCGTCAGGATTTTCCCAGGGAAAGTCAACCGATTCCCCCGGTGATGCCCGAGGAAACACGGATCGAGGCAGCTCATGCTATTTCAGCACCCGCTGGTGTCGAGGCCAGCTCGGATGTCACTTCTGCGTGATAAACACGCCAGAAGGACAGAAATTCCTGAAGGGAGACAGTAGTTTCAGGATGCCCATGAAGAAACCAAAGGGCCGCGTAACTGTCACTTTATCATCTATATATGACAACAAAGATGGTAACTTTAAGAATGTTGTCCCTAGAAATTCAGATCCCATGCCGCTGACTTTCAGGAAATCAAATAGCGGCGGGCATATTCTAGTTCCTTTTAGGAGAGCTGAATATGTGTTGATAGACGTGATTGAAGGCGATTATGGGTGCGAAACTATCTCATATAGCTACTATGGCAGCGTAGTCAAGGCCGATACTATATGTAGGGGGGGCATGACATATGTGTATTATCACGTCGATCAACTTTTGTCTCCTATGTCAAGAAATATACTTGGAATTCTATCTAGACACTTTATGGATGACTTCACCGGGTACTACAACGATATGTGTTCACTTGACAACGTGTTTTTGGGGTCAGGGGTGGCATCTCCACAGCAACGTCATACTCTACACTCAATTAAGAACCTTTCAAAGGCTAAAATTTCAGCGGAGCACCATATTCACTACACTGCAGAAGAGGTCTGGTCAACACTTGACAGTGCACAGCGTAGTAAGGCCGAGCACGCGCTACGAATAACAAACGAGGCCACAACCACTATGATGGGCGGCGTCATGTTATGGCTCGCCATGCTGCCAGATGAACTACACAAAAGGTTTGTTAATACTGACATTTTAGATGCGGACACTATGGTCGAATTTGCTAGGCGTGCTAAAAAACTGTCTGTAACAGCTAAATCTTATCAGAATATAGTCGAGGTAGACCTCAGGACAGTATTTGAAGTTGATGTGTTAGTGAACCGCGACGTAGGTAAGGTTGACTGGGAAGGGGAGAAGCAGAACAGAGTGAAACCTGATACTGTTAACATATCGAAGAAGACAGTCTATGACGAGGCTAGAAAATTATTTTCAAGAACCGACAATACACGTCTCAAACCAAGAAAGTTAAAATGGGAAGACTTTTGGAAAACGCGATGGCAGTGGAGTGCTTCTGGGTCAGTGCACAGCCAATATGCCATAGACATCCAAAATCTACCTAAGGAGAGAGAATTAAGAAATAAATTCATATTGCTAACTCAGACACCCTACAGAGAGTTTGATTTTTATGCAACTCGTAAGCCTCAAATTCAGGCGTGGTCATCGGTTAAGTACGAGTGGGGCAAGATGCGGGCAATTTACGGTACGGACTTAACGAGCTACATCCTAGCTCACTACGCTTTCTATAACTGTGAAGACACTCTACCAAATGAGTTCCCAGTCGGCAATAAGGCTCGACCTTCGTACGTCAGTGCTAAAGTCGGCGCTATACTCAAAGGTAGAATACCATTGTGTATAGACTTTGAAGACTTTAACAGTGGACACAGAAATGATTCGATGGAAGCGGTATTGCAAGCCTACATCGACGAGTTTCATGAGGATCTGGATCCTATGCAGTTGAGTGCGGCAGAGTGGACGAAACAGTCAATATCCGCCACCATCGTAAACGACAATATGGGGACGAAGACGCAATACAAAACAAACGGCACCCTAATGTCTGGCTGGCGTTTAACAACCTACATGAACTCAATTCTCAATTACATATACACGAAACTATTGACCAAGGATACAGAAAGCACCTACCAATCGGTACACAACGGTGATGATGTACTGCTCGGGGTCAGAAACTTCGATATAGCGAGGAGGGCTGTATTTAATGCGGATAAGTACAATGTCCGTTTGCAACGCAGCAAATGCACGTTCGGGGGTATAGCGGAATTTCTGCGTGTTGACCGCGTTCGAGGTGATTTCGGTCAGTATCTGTCAAGGAACGTAGCAACGTTAATGCATGCCAGAATAGAGTCCAAGCTAGCCCTAAGCGTAGTGGACTTGGTCGAGGCATCTGAAGAACGTTTGCGTGAATTCATACAGCGAGGTGGCTCACCTAAGACTGCGGCCAGGTTGAGGAGTATCGCGTATGACAGATATTCCAAGATTTATGAAACGGACACAGCCACTCTCTACCGTATCAAATATTCTCACCGTGTAGCAGGGGGAATATCTGATGGCCTCGGAGCACCTATTGATCAGGTGATAAATAAGGATCAAGTCGGGCGGATAGCGGAGCTACCGGACTATCTGCCAGGCATAGCAGACTATTCAAACGTGCTCAAGAAGAGTTTAAATTTGAATATGGAAGTAAGTAAAATAGCAAAACGGATATACAGCGCCACACTAAATGCTGTTAAGTTAGAAAGGACTAAGGTTCACACCGAGGTACCAGAGAACATAGAACAACTGAAAGTGTACAGAGCTCTGTATAAAGCTCACAGCGATGCAACTGATAATGCTGCATTTGGAAAGGCGATTCTAACAGGATTCGTATTTGACGTGCTCAGTCGTAATGACAAGGCGAACACGTTGATGGGCATCTTGTACCAATCGAAGGACCCGATGCAATTGTTAAAAGTAATAGCATGAGTGGGCTGCGCCACAAAAATAGCGCACCTGTCACACGTCA

UrV4_ORF1

MAKLFIKDSIKTNLFSTRDPIFPGARFTVANNSTIVVRHQERQFLSGLSLTADFQAAGRLKKVINAAVQTNYSGFNKKYINEAGVYDGSLALDEFAKSGADRGLVRPETYSMLTKYPQADSHESFIYNMLVSYLKAKLSTGNLTEDEDLKVETSPYVDSHCVVPLDQAYEDFTYEIELGSPVDGRLAMQGQFMVRNKDNYWSKPYVLHYNGTSTKAESFYLLHAMGRNVVSELNFDFPIKGADTAHMLIDPVNGREFATIDSSEIDWTDHESMWLWILDYVQLNRLEQAFAAAFETLGALAFQPLPPTAEACQWQQAQLTLTLARFSPTRARLRNNLAGEPYKVDSLADEFLISETASASQFLGASAICNYYMWYGLYTILQNEASEIEQWQNVYTSIHGVLQNLYSPAMRAMCISVATGKEFATCMTDNCAMFIDMSRLEVMPKITNIKTLDASVPAEIVVDHIPAPVSGAIVLGTFTDEYDTTAHLSAVFSLPTSDDPYARFSETELLKIATVYRLFGYDTELVDVITDIPMALWAANRECIPDPSKLLAYRRLRRDWIISDISPRDGRKEVIDSIQTLTSGKPATVTIQQPTISFTSWRQRVRTLKPQVIVAKKNKKKEIRFKVNASVRMLDTTLMARPIAAVSRQDFPRESQPIPPVMPEETRIEAAHAISAPAGVEASSDVTSA

UrV4_ORF2

MPMKKPKGRVTVTLSSIYDNKDGNFKNVVPRNSDPMPLTFRKSNSGGHILVPFRRAEYVLIDVIEGDYGCETISYSYYGSVVKADTICRGGMTYVYYHVDQLLSPMSRNILGILSRHFMDDFTGYYNDMCSLDNVFLGSGVASPQQRHTLHSIKNLSKAKISAEHHIHYTAEEVWSTLDSAQRSKAEHALRITNEATTTMMGGVMLWLAMLPDELHKRFVNTDILDADTMVEFARRAKKLSVTAKSYQNIVEVDLRTVFEVDVLVNRDVGKVDWEGEKQNRVKPDTVNISKKTVYDEARKLFSRTDNTRLKPRKLKWEDFWKTRWQWSASGSVHSQYAIDIQNLPKERELRNKFILLTQTPYREFDFYATRKPQIQAWSSVKYEWGKMRAIYGTDLTSYILAHYAFYNCEDTLPNEFPVGNKARPSYVSAKVGAILKGRIPLCIDFEDFNSGHRNDSMEAVLQAYIDEFHEDLDPMQLSAAEWTKQSISATIVNDNMGTKTQYKTNGTLMSGWRLTTYMNSILNYIYTKLLTKDTESTYQSVHNGDDVLLGVRNFDIARRAVFNADKYNVRLQRSKCTFGGIAEFLRVDRVRGDFGQYLSRNVATLMHARIESKLALSVVDLVEASEERLREFIQRGGSPKTAARLRSIAYDRYSKIYETDTATLYRIKYSHRVAGGISDGLGAPIDQVINKDQVGRIAELPDYLPGIADYSNVLKKSLNLNMEVSKIAKRIYSATLNAVKLERTKVHTEVPENIEQLKVYRALYKAHSDATDNAAFGKAILTGFVFDVLSRNDKANTLMGILYQSKDPMQLLKVIA
