# Supplementary material for: Detection and Molecular Characterization of Novel dsRNA Viruses Related to the Totiviridae Family in Umbelopsis ramanniana
Source: Front Cell Infect Microbiol. 2019 Jul 11;9:249. doi: 10.3389/fcimb.2019.00249 (PMC6644447; doi:10.3389/fcimb.2019.00249)
Supplement: Supplementary file 7 [file Data_Sheet_2.docx]

**Supplementary Data S2** Multiple sequence alignment of RDRP amino acid sequences made by MAFFT v7.312 using the E-INS-i option.

>UrV3_longer

MAPD--------------------------------------------------------------------------------------------------------------------------------------------------------------------------------------------PQ-----------------------------------------------PVREDV-VEVQNVEWTREA-------------------LKG-----------------------------------------------------------------------------------------------------------------------------------------------------------AYERP---------------------PRQYSDM-----------MKVSML------------TVYDI---------------------------------------------------------------------------------------------------------------------------GLFDEP--------------------------------------------------------------------------------------------------------------------------------------------------LVREAAEAQRGQLEYAV-------------------------------------------AALCLWLTT--------DVAK------YLC-------AELPVH----------------RVPLSKWPGSVKAFANDARRLGQVFGRGPQEVAMAFR----LRRLVSLA---GRSTADADWEKE-----VAERTQLTTAKRAFA---------------DGEVSSAAYRLIRDKVLHRIAVQVVNSLKKSGGSFDEYFEQRWWNTPRGTTSKGGDVKRQLK--------------------NADKHLDLQMRPIKPTVMELYSKPGLLQDLRGLPYC-------VARGSTKP-EPGL-KCRALLAVDDRTAIVAGYASSGIETTTKE--GGMVLRQDPAD--------------VAEWV-SFDL-----------GPGVWRVSNDYSNFNGLNSL-RSMQL-VDLH--LAQEWRRVP------------------------ERWAEEK---------------------ALASE-WVAASYLNPYMKTP--LGET--RVVSGLWSGHRNTARDNTFLHLVYLECIKSVMRALFGQHAKHGKVRLCGDDETLGY--------DEWCAAVLHTVVADELGFTSQVSKGMLSRKHDEFLQLLRQ----P-GKVPSYPIANTILTFCSGN-WYKDPVRDLNT---------------------------------------------TVADVSDHLWDLVLGGVD---------PDVCQR---LGVYVLDYLMQVKRSDGSLFPLEWWDFRGSGIPGGHPLWG---------------GFE---TPAPPQIKVK-----------LP----------TIKLPM----------------AATQDSVKREWP--VWERL------------EKHRLAETM----------------NERAWSSYRVVAKHWLQEEY--------------------------------------------------------------------------------------------------DKA-----------AQEEWPARR-DCVK-------------V--------HIPVVRREVP--TNRWRAIGDRNRARSARAVAVKCGFPPELLGS-----------------DDMWKAMAW------------LSP---------------RDRSNMYAGLA-----------------ERQSTTKGWRWEMP------------------------------------------------------PLLRTD--------------------------------------------

>UrV3_BLASTHit_BAU79517.1_RNA_dependent_RNA_polymerase_Diatom_colony_associated_dsRNA_virus_17_genome_type_A

M-----------------------------------------------------------------------------------------------------------------------------------------------------------------------------------------------PQ------------------------C-----------------------------ALVEDYTTLRHE----------------------CT----------------ITSYVAMTPP------------------------------------------------------------------------------------------------------------------------------LPQHWPTLEDCKNN-------------YVAFPAGLG-------GPIKFTITTIS-----------------------------------------------------------------------------------------------------------------------------------------ELFPNP-----------------------------------------------------------------------------------------------------------------------------------------------IQPTTALILHFNVGAVEAQV-------------------------------------------AAMLYWVHTAPR-----KAVD------VLT-------SHGWFH-----------------KPLKQWADVFKDSLDTMRRQATVPGSLN-DHETFSA----LRKIMSLT---YRGTEEPDWDAE-----MSRMRENMPIRSFN------KLPRALHLNYNRELWNAIKEVTATTVH-------NVVRNTRQRSMDEWWATRRAWAPSGSSTNRRLLDDYK---------------------KREPRIKSADRASKKTVVEALPDDYMETSLARPPIS-------LARRSKKS-EAGW-KARALYASDDVPFFIASYASLDMEKNMNH--DGMNPRQTPDD--------------VAAWVL-ADA---------RTTMSDVWLSLDYSDYNKEHRN-TELAL-LNLG--FVLAWAVAP----------------------VDPSVRVDK---------------------MRCAL-WTAKAHLCAFVS----DGEHTHRIFSGLFSGHRDTARDNTLLHAVYSKLARKVVSRITGARCEPRFIAMCGDDEDACF--------NSDIMAMMYLHVHSWANWTLTDRKQMLGNAYHEYLQRSAFN---R-QLPTKP-LATILETMATGN-WYKRSATWYDS---------------------------------------------VISAVSDNCFEMVRRGVR---------LENAQA---LARKLISAMMKIKRSDGTIKKLEWWTYR--DPSASHPLWMSQEP-----------------TATPPITSAK----------PRP----------SYSAPT----------------HATDAYIKHAYS--ILTHLEPK--------------KLVL--------------YKNEMLCESYGSIHTSWRMDLN----------------------------------------------------------------------------RQAAEQLWPERHSEP---------------------LLLEAP------------------------------QPPDAREKQDWVIQGMLCRAKTNTPTSEREAIARMGLDPSLVKYL-------GGVSKAWRLTDPHKLARY------------QEP---------------VEPCALNPILY-----------------YIDSALVSWMTATP------------------------------------------------------HIPRDYR-------A-----------------------------------

>UrV3_BLASTHit_BAU79519.1_RNA_dependent_RNA_polymerase_Diatom_colony_associated_dsRNA_virus_17_genome_type_B

M-----------------------------------------------------------------------------------------------------------------------------------------------------------------------------------------------PQ------------------------C-----------------------------ALVEDYKLLRHE----------------------CI----------------ITSYTAMTPL------------------------------------------------------------------------------------------------------------------------------LPQSWPTLDECKTN-------------YAAFPTGLG-------GPIKFTITTMA-----------------------------------------------------------------------------------------------------------------------------------------ELFPNP-----------------------------------------------------------------------------------------------------------------------------------------------IQPTTALVLHFNKGAVEAQV-------------------------------------------AAMLYWVHTAPF-----KAVD------VLT-------THGWFH-----------------KPLKQWAEVFKDSLDTMRRQATVPGALG-DHETFSA----LRKIMSLT---YRGTEEPDWDAE-----MSRMRENMPIRSFS------RLPKELHLNYNRELWSAIREITATTVH-------NVVRNTRQRSMDEWWATRRAWAPSGSSTNRRLLDEYK---------------------KREPRVKSADRASKKTVVEALPDDYMESSLAQAPIS-------LARRSKKS-EAGW-KARALYASDDVPFFIASYASLDMEKNMNH--DGMNPRQTPDD--------------VAAWVL-ADA---------RTLVHDVWLSLDYSDYNKEHRN-TELAL-LNLG--FVLAWATAP----------------------VDPTVRVDK---------------------MRCAL-WTARAHLCAFVS----DGEQTYRIFSGLFSGHRDTARDNTLLHAVYSKLARKVVTRITGAPCAPKFIAMCGDDEDACF--------SSDIMAMMYLHVHSWAKWTLTDRKQMLGNAYHEYLQRSAFN---R-QLPTKP-LATILETMATGN-WYKRSATWYDS---------------------------------------------VISAVSDNCFEMVRRGVR---------IENAQA---LARKLISAMMKIKREDGSVKKLEWWTYR--DPTASHPLWMTLEP-----------------TAAPPVTSAK----------PRP----------SHAAPT----------------HATDAYIRHAYS--ILTHLEPK--------------KLVL--------------YKNEMLCESYGSIHTSWRMDLN----------------------------------------------------------------------------RLAAEQLWPERHSEP---------------------MLTEQP------------------------------QLPDARDKQHWVLHGMLCRAKTNTPTSEREAIARLGLDPSLVKYL-------GGISKAWRLTDPHKLARY------------QEP---------------VEPCALNPALY-----------------YIDSALVSWLTATP------------------------------------------------------HIPRDYR-------K-----------------------------------

>UrV3_BLASTHit_YP_009333150.1_hypothetical_protein_2_Beihai_barnacle_virus_15

M---------------------------P----------------------------------------RGDGSGTAPRCPLLPPPYG----AGADG-------GPESGLSAGLRRASEDP-----------------------------------------------------------------------PKVGDL--------------------CA---------------VGFGVTPVRGDA-----------------------------GPTL----PPGVGPHRL-------ANNLLREAER--MVKTGDR----------------------------------------------------------------------------------DSSGAGVTRWGGLLRALYFG--------------------------------------GAAGAEDYEAA------------------------------------------------GLA----------------------VWPPPQT--------------------------------------VAPRVAADASLPD--------------------MSI---EALKER---------------------------------------------------------------------------------------------------------HPQGYGGSGAVIQLTLTT-TFKALGRGP-----------RRWECELMNANVGHPEWSV-------------------------------------------GAVILFFSALEP-----DVVD------WLV-------EEARVH----------------LIPLATWPECLRDWFTALRRCPRLRGM---PHVSPSA----VLELRKMLNCVLRSNDEADWKHE-----YTRKCAEQAIHTGVGAN-----GMLSQAVWYNDLAVSIKEYVDGTIN-------ATVEAREPETASEFWRLRWERGAAGSSSERKRLADLC---------------------AADERLGGQARANKKAVLEAMTDADFETMWEGLPSY-------VARGSTKP-EPGG-KQRALYATTDECFILSAYGSADLEKYMNI--EGIRAKQTPADVVEW----------VKQGM--------------DMAPQARWVSLDYSDYNWEHTT-AALML-MELC--FAAAWCRKG----------------------GDREWGADK---------------------TAAAL-WSMWAHAAKFTVVP--KVDASWRFFGGLFTGCRNTARDNTLLHGAYSKTIEKYLGMVDPG-KYLMHKNYTGDDEDSVL--------PDWVAAANYLVLHSLAGFAIKPAKQMCSRETHEFLMRLAMP---A-VLPTRP-LGTALAIFTSGN-WYKDVHVYYDS---------------------------------------------IISGVSSNVWELVARGLP---------LVVGRR---LAAATLNAQMRLKNADGTRTLLEWWRYRHGTGAAEHPLWAGTPGDWEP-VPEVVSPLE----------------------------------V-HPDAPT----------------HATNAWVKEKQR--SLGLPATK---------EWDVVRKTA-------------------VAASFGKMYSRHAAHMHEA-EVRRVWPRRESHPHGLDVPGPPKPDQDELKNLMYVYPIDRRPTTEPEVFGRIGLTPAIVAAFGGIKRVLLYLPVNRLAKYVEPAPPQGLRLELYWEDSAIQHWHRTAGLSGEKAP-RLADAVYAR--------------------RWPRLQRSEA------------TRDTTTRVLILAPNGAGK---------------------TTYCRSHQWVFDSDEMLGKVVEKSSFRALRYRPDVERPSHVASAVEHILLRRDR-------------YGIATQLDVDLVSLMPSQRGWEWQIYIVQPPRAELETRLRARGWDDTKIARRLDHWEASVRHGVRKSKHLTSKEREGAHWCSEWPEKI------------------------------

>UrV4

M----------------------------------------------------------------------PMKKPKGRVTVTLSSIY----DNKDGNFKNVVPRNSDPMPLTFRKSNSGGH-----------------------------ILVPFRRAEYVLIDVIEGD-------------------------------------------------------YGCETISYS---YYGSVVKADT-ICRGGMTYVYYH----------------------------------------VDQLLSPMSR---------------------------------------------------------------------------------------------------------------------------------------------------------------------------------------------NILGILSRHFMDDF---------------TGYYNDMCSLDN---VFLGSG---------------------------------------VASPQQRHTLHSI--------------------------KNLSKA-------------------------------------------------------------------------------------------------------------KISAEHHIHYTAEE-VWSTLDSAQ-----------RSKAEHALRITNEATTTMM-------------------------------------------GGVMLWLAMLPD-----ELHK------RFV-------NTDILD----------------ADTMVEFARRAKKLSVTAKSYQNI------VEVDLRT----VFEVDVLV---NRDVGKVDWEGE-----KQNRVKPDTVN----------------IS-KKTVYDEARKLFSRTDN--------TRLKPRKLKWEDFWKTRWQWSASGSVHSQYAIDIQN------------------------LPK-ERELRNKFILLTQTPYREFDFYATRKPQI-------QAWSSVKY-EWG--KMRAIYGTDLTSYILAHYAFYNCEDTLPN--EFPVGNKARPSY-------------VSAKV--GAI-----------LKGRIPLCIDFEDFNSGHRN-DSMEA-VLQA--YIDEFHEDL---------------------------DPMQ---------------------LSAAE-WTKQSISATIVNDN-MGTKTQYKTNGTLMSGWRLTTYMNSILNYIYTKLLTKDTE------STYQSVHN-GDDVLLGV--------RNFDIARRAVFNADKYNVRLQRSKCTFGGI-AEFLRVDRV----R-GDFGQY-LSRNVATLMHAR-IESKLALSVVD---------------------------------------------LVEASEERLREFIQRGGS---------PKTAAR---LRSIAYDRYSKIYETD---TATLYRIKYSHRVAGGISDGLG--------APIDQV------INKDQVGR-------------------------IAELPDYL--------------PGIADYSNVLKK--SLNLN-----------MEVSKIAKRI-------------------YSATLNAVKLERTKVHT------------------------------------------------------------------------------------------------------------EVPENIEQLK------------------------------VYRALYKAH----------------------------------------------------SDATDNAAF------------GKA---------------ILTGFVFDVLSR----------------NDKANTLMGILYQS----------------------------------------------KDPMQLLKVIA-----------------------------------------------

>UrV1_RDRP

NGCQTYRKTEAGFYQGQHVGTPSTPRDPPRRRNRCYGSTEWCTGGVSCRFCGINQTKASLNLYSSESGYRVPAYPPQRRSTFYHWQPS----LDGAGEKRKYLALERDEAPTSFRVSDLGNY-----------------------------IVVPFKRAQYVLIDMLDEA-------------------------------------------------------YPEGDCMYN---YYGSVFNCLM-LPSKKRTYVYYK----------------------------------------VDQLLRPRSR---------------------------------------------------------------------------------------------------------------------------------------------------------------------------------------------NILAILSRHYMDNF---------------NGYYNDWCSTEN---VFAGLSK--------------------------------------VSEVNFRHSIDKL--------------------------EQLPIA-------------------------------------------------------------------------------------------------------------KISAAHHIHFTAAE-IWQCLDSEQ-----------RDKAKHALRITAQATTTMM-------------------------------------------GGVMLWLAMLPT-----ELFE------HFV-------KTDILD----------------ADSMVEFAKRAKKLSVQAKSFQNI------VEPDLRT----MFEVDVLV---NRDVGQVNWDAE-----KANRVTPDLVN----------------LN-NKRIYDAAIKMFSRVDA--------TKQKPRRMSWRDFWMSRWQWSASGSVHSQYAEDLKD------------------------LPK-ERELRNKFIQLCQAGNYDANHFLQRRSEI-------QAWSSIKY-EWG--KMRAIYGTDITSYVLAHYAFYNCEDVLPN--EFPVGLKARPSY-------------VSAKV--QSV-----------LERKVPLCVDFEDFNSGHSN-QAMQT-VIQA--YYDVYSAGM---------------------------DDDQ---------------------KRAIL-WTRDSVARTQINDN-MGTKTSFSTNGTLMSGWRLTTFMNSVLNYIYTQQLLENCG------EHVNSVHN-GDDVLLGV--------SNFDIARRTVYNAEKYNIRLQRSKCAFGGI-AEFLRVDRV----R-GDFGQY-LSRNVATLMHSR-IESKVALNVVD---------------------------------------------IVEADEERFREFVRRGGD---------EAVVSR---LRHLSYKRTAKIYDTE---LSTLYMIKSSHRVVGGISDLDN--------APVDCI------IEKDKTGK-------------------------ILPLPDQL--------------PGVMDYAIMLKK--SLELT-----------VSTREVYKRV-------------------YNATLNAVQLVRTSVKH------------------------------------------------------------------------------------------------------------TYNENIRQYE------------------------------VFRALYKAH----------------------------------------------------SDTTDTPLF------------GKA---------------MLTGFVFDVLNK----------------SKNMTTLIRMLQQS----------------------------------------------ADPMRLLRVVA-----------------------------------------------

>UrV1_BLASTHit_YP_009342428.1_hypothetical_protein_2_Wuhan_insect_virus_26

M---------------------------------------------------------------------------QERDSFTALDIC----DGMREGREHTFAVRSGHVPTGLRITCNGTY-----------------------------LGVPFKQAELLLIDRLPAK-------------------------------------------------------LHECNVAYD---YYGTVVSARL-VPGRDATYVYYY----------------------------------------VDQDIQPVTQ---------------------------------------------------------------------------------------------------------------------------------------------------------------------------------------------NLLGMLSRHFLGDF---------------SGYYNDWCSLDN---VFYSLSK--------------------------------------CDNPVKRHTIKTI--------------------------RDLPKP-------------------------------------------------------------------------------------------------------------KISGAHHIHYTASE-VWSVLDDRG-----------KDAARHALRLPADATTSFV-------------------------------------------GGVMLWLASLPT-----ELLN------PII-------ESDLLD----------------AEDTIEFGKKAKKLSVTAKSFQNI------VEADLRP----IFEADVLV---NRDVGEVDWAGE-----KNNRCKPNLAS----------------VS-PGEVYARTLALFTKEDD--------EKALPRRLEWEKFWDARWQWSASGSIHSQYQEDLDS------------------------LPK-ERELKNKFIALSMQGDMPFEHFLNRKPEI-------VAWSSVKY-EWG--KMRAIYGTDLTSYVLAHYAFFNCEDTLPT--DFPVGEKARPSF-------------VSARV--AAI-----------LEGTVPLCVDFEDFNSQHSN-DAMEA-VVQA--YIDAYSQYM---------------------------TPEQ---------------------VQAAM-WTRESISNTRVIDN-MGTKTSYKTKGTLMSGWRLTTFINSVLNYVYTQALISGSH------AMSRSVHN-GDDVLLGI--------RNFKIVRDIVARADKYNVRLQRTKCAFGGL-AEFLRVDHM----R-GDYGQY-LTRNIATVMHSR-IESKIAVSAVD---------------------------------------------VVQAMEDRLREYLQRGGV---------WDYAVQ---LRELYYDRMAPIYSLR---PQDLYDIRTAHRVVGGIAEDVS--------ARVDKF------IRTSSESI-------------------------ETTLPDTL--------------PGVDAYARLLKK--VLELE-----------VPVSVVSTRI-------------------RNATLNAVQLTRKTVTV------------------------------------------------------------------------------------------------------------EKTLNVQRYM------------------------------VYRALYRAY----------------------------------------------------ADVTNNPMF------------GKA---------------MLTGFIFDVLTQ----------------NAQLSAVAQLVSSA----------------------------------------------QNPMEFLRVIS-----------------------------------------------

>UrV1_BLASTHit_YP_009342434.1_hypothetical_protein_2_Wuhan_insect_virus_27

M----------------------------------------------------------------------TVEPTEERSVFR------------------DYRFRDGAVPTGIRISGGGSL-----------------------------EAAPFSDADFVLVDRIRGK-------------------------------------------------------FNQGPVVID---VLNTSAAGFA-VPGPGYTFLYMR----------------------------------------VNQELVPVNP---------------------------------------------------------------------------------------------------------------------------------------------------------------------------------------------QVMALAARHFMGDF---------------NVYYNDLSDLKN---VRLVLGE--------------------------------------SDNKIRNN--ATL--------------------------QALPKP-------------------------------------------------------------------------------------------------------------KISGSHHIHFTAAE-VWGVLTDQE-----------KERAMQYRRLGCDLTTSFV-------------------------------------------AGVMIWLVSLNP-----ELYE------LVA-------ASDLLD----------------SMTVKEFAKKAKRLSVQAKSLQNV------VTQDLRV----LFEADVLV---NRVTGEVDWAGE-----KEHRTKCNLAQ----------------IS-PQRVKEVATRLFNENNA--------LGERPRRFEWKKFWNNRWQWSAAGSIHSQYSEDMQF------------------------VSK-QRELKNKFIALITMPDMPIEYFLERKPEI-------RAWSSIKY-EWG--KLRAIYGTDLTSYVLSHFAFYNCEDVLPA--HFPVGKKARPSY-------------VRSRI--RSI-----------LSGSIPFCIDFEDFNSQHSN-ESMIA-VIEA--YLEVYQESF---------------------------SKEQ---------------------IMALQ-WTIQSIKETHVIDN-MGTKTEYQTKGTLMSGWRLTTFVNSVLNYVYTRELIGESK------TVTRSVHN-GDDVLVGI--------TNLAVARDVVRNARKYNVRLQTVKSNLGSI-AEFLRVDHA----R-GEYGQY-LTRNIATLVHSR-IESQKAVSLID---------------------------------------------LIEAMEDRFGEFFTRGGS---------MDLITR---LRDKYYQHVAPIYNAT---VEIAYKIKQAHKVVGGASRLAD--------ASINYI------IKQAKTVS-------------------------EVELPKKL--------------PGLDSYADELIA--SLGLS-----------RHRKKVRERV-------------------QQATLNAVQLVRKAISV------------------------------------------------------------------------------------------------------------VSNVDMKRSR------------------------------VLRALYKSY----------------------------------------------------ADVADSPMF------------GKA---------------KMTGFVIDVLAN----------------STKLRTLSMKLRGS----------------------------------------------QDPIALLSVIT-----------------------------------------------

>UrV1_BLASTHit_AMB17470.1_RNA-dependent_RNA_polymerase_partial_Delisea_pulchra_totivirus_IndA

-----------------------------------------------------------------------------------------------------------------------------------------------------------------------------------------------------------------------------------------------GSSVEGII-IPNSGCSHFYMK----------------------------------------LNQYVRPRSN---------------------------------------------------------------------------------------------------------------------------------------------------------------------------------------------DLMAMLGAHFSGF----------------PVYLSQPASTNN---ILSYVEEKTNKKY-------------------------------KNKKPKRRHEIKNL--------------------------HKLPKS-------------------------------------------------------------------------------------------------------------KISGDHHIHFTAEE-IFKTLKPDE-----------KRRALLALNLPADATTTFM-------------------------------------------AGVMLWLATLNS-----RELD------VII-------KAGLLS----------------TDTQKEFGIVGKAISVRAKSLQNL------AEYDLRK----LFEIDVLI---NRYLGDPIWEEE-----KEKRTNPDLVK----------------LE-PNLVYNAAKRLFQQSDR--------NRERPRKMSWEKYWNTRWQWAATGSIHSQYDVDKEV------------------------VAK-ERELKNKFISLCKMDKKTMSYFTDRKEEI-------RAWTSIKY-EWA--KLRAIYGTDLTSYILTNFVFFNCEDTLPA--QFPVGKKARPSY-------------VSAKV--ASV-----------LESGEAFCLDFEDFNSQHSL-ENMQA-VMEA--WLSVYRDDL---------------------------DEEQ---------------------VRAAR-WVINSVADTTIEDL-EGTGTSYKVKGTLMSGWRLTTFVNSVLNYIYTQMIVAGSD------EFPQSIHN-GDDVLLGV--------KKPGTIAKMMKNARELNIRVQHSKCAYGGI-AEFLRVDHQ----R-GDFGQY-TTRSIATMMHAR-IESKVAVTTTD---------------------------------------------YVEAMESRLFDFAVRTGN---------MNLTSR---LRLQYYRRLEELYDLS---VKDLYRVKRAHRVVGGASDKKD--------ADLEWR------ITHNEPEK-------------------------IVELDSFL--------------PGVRDYTKKLIS--ELDVD-----------LDYEKVYDTI-------------------YKSTLNAVQLVRKETKI------------------------------------------------------------------------------------------------------------TFTNRDARYK------------------------------VYRGIYGAY----------------------------------------------------KGINDNGVI------------G-------------------------------------------------------------------------------------------------------------------------------------------------------------------

>refseqp|YP_009507835|820_AA|Xanthophyllomyces_dendrorhous_virus_L1B_Totivirus

M----------------------------------------------------------------------TSESVQHCEELNGIKIIKSVRNKA-----MKMEMRDGYVPMCIKARPGIVYK---------------------------WEATAFCDSEYVLVGSDMSE-------------------------------------------------------PSENESNVN---IMGVLMSGLR-LAGDNCTYLYAK----------------------------------------VDQYLVTRKK---------------------------------------------------------------------------------------------------------------------------------------------------------------------------------------------AVIAIMTRHFNGLYG--------------SVFLNDPLNMEA---MFRDRAP--------------------------------------DDELMPKQNYEKI--------------------------CKKENT-------------------------------------------------------------------------------------------------------------KITAQHHIHFTSEE-VVRVLGKRT-----------AEES-EATRLPADATMSMA-------------------------------------------AGVLLWYNELSP-----VLKE------MIL-------KCGLFK----------------SKTIQAFKKIAKDISVEAKSLQNI------VQTDLRS----VFEIDTLI---NRIDGEVDWAEE-----RDHRVNPNVTN----------------LS-YSDVYDAAKDIFLQAAA--------VGRKPVSMDWDKYWASRWQWSAAGSIHSQYVEDDKY------------------------VIRTDRNLKNKFIAIANMPKYGYDFFMSRKPQM-------HAWASIKY-EWG--KLRAIYGTDLTSYVLSNFAFYNCENVLPK--RFPVGKDANDAN-------------VVNRV--AGV-----------LKDRLPYCLDFEDFNSQHSV-SSMKA-VIYA--YGDVYRQVF---------------------------TEQQ---------------------LEALA-WTAESLDDVSVNDN-VGLKQTYKSNATLLSGWRLTTFVNSVLNAVYTDKICGEAK------MPGSSLHN-GDDVLIGA--------TSMKVARESLRRSELYGIRVQASKCAFGGI-AEFLRIDHA----R-GSKGQY-LTRAIATLMHSR-IESKLSTDARD---------------------------------------------LIEAMENRFSDCLNRGMT---------LDTVTK---LRHVYYNRQSVICNMP---VEDFYRIKTTHRVAGGVSEAID--------SDVSMS------VIRGIDKSF------------------------NIKIPK-L--------------QGVHDYARAVAM--ELEMM-----------KRLDYITDRM-------------------YKATYEAVVPKTRGMTV------------------------------------------------------------------------------------------------------------VPNKEEKWCY------------------------------NVKAIYKAF----------------------------------------------------KGKIQTAGY------------GKA---------------ALVGMALDVIQT----------------SDKDTTLKMALARS----------------------------------------------PDPIKLLRYLV-----------------------------------------------

>UrV1_BLASTHit_BAT62484.1_RNA_dependent_RNA_polymerase_partial_Red_clover_powdery_mildew-associated_totivirus_4

----------------------------------------------------------------------------------MGIDIMVISKSDARGLREMDIRLRDGYVPACIAKSKGGIL------------------------------ATEFADAEYVLMDVSEGP-------------------------------------------------------SSDKEVVVS---IFGVAMTGIM-LQGVTKSYFYAR----------------------------------------IDQYIITRSK---------------------------------------------------------------------------------------------------------------------------------------------------------------------------------------------AMLALFTRHFSGLYG--------------SVFWDTPLSTDA---LYRDRLP--------------------------------------DEKPAKSMTPMEI--------------------------ESLGYP-------------------------------------------------------------------------------------------------------------KITSGHHIHYTYSE-VLKSTPRDK-----------LNRVAELARLPSNASATMA-------------------------------------------TTVLLWGITASK-----EAYE------GVL-------MSGLFK----------------TKTTKEFTRIAKSISVEAKSLQNI------VEADLRE----VFELDVLL---NRVDGEVDWIQE-----KENRENPKLAK----------------LD-GGYVREQAQIIFRQAVQ--------CGKKPVSMKWDAYWASRWQWSASGSVHSQYVEDDKY------------------------VIRTDRNLKNKFIMISNMPHFSSEHFINRQPEV-------HAWSSTKY-EWG--KLRAIYGTDTTSYILANFAMYNCENVLPN--RFPVGKAANDRN-------------VVSRV--RGV-----------LKDRLPYCLDFEDFNSQHSP-EAMRA-VVLA--YGDVFNKTL---------------------------TEDQ---------------------MRALQ-WTADSISNQIIHDN-TGTKTVYKANGTLLSGWRLTTFVNSVLNAIYSDAVLKGIK------RQGSSLHN-GDDVIIGA--------TSMEVARRSVQVGRKMGIRIQPSKCAFAAI-AEFLRVDHA----R-GSKGQY-LSRACATLVHSR-IESKPSTDARD---------------------------------------------LVESMENRFADCLARGMS---------LQIITN---LRLRYYARQAVVCKTP---IEAFYVIKNTHRVAGGISEAHD--------ADVEQL------IKPGPAGKL------------------------EVEVPK-L--------------IGVHEYAVEVAK--TLDMM-----------KDVELLVSRV-------------------RMATLSAVTPKSRKMHI------------------------------------------------------------------------------------------------------------IPNPNVQASK------------------------------HLKAIYKAF----------------------------------------------------KGKVDVAGY------------GKA---------------A-------------------------------------------------------------------------------------------------------------------------------------------------

>refseqp|YP_007697651|761_AA|Xanthophyllomyces_dendrorhous_virus_L1A_Totivirus

M----------------------------------------------------------------------------DRESSNKFGIF---------------------------------------------------------------------------------------------------------------------------------------------EASKS---VYGVTMSGLV-IANGKAKFFFAS----------------------------------------IKQYLLTQSK---------------------------------------------------------------------------------------------------------------------------------------------------------------------------------------------AMLIAMTRHFSSLYE--------------SYAYDDPTSPVN---LFRDRFP--------------------------------------GRSENEAMTFKQM--------------------------EELPKS-------------------------------------------------------------------------------------------------------------KISGAHHFHFTPMQ-VLKSIGRGR-----------LERAIQASRLPKDAEMTFA-------------------------------------------TGMLLWYTSISD-----QMAE------LIR-------GAGIFK----------------CDSVREYVKEMKQLSVEAKSLQNL------VTDDLRT----VFELEVLV---NRIDGVVDWEKE-----KENRQSVNVTN----------------IK-DSDVFRSACKIFEDAKG--------IGRRPKSFSWESYWANRWQWSAAGSIHSQYPRDMEY------------------------VIRDQQSLKNKFITISNMPKCTVDYFSDREPQV-------QGWSSTKY-EWG--KQRAIYGTDLTSYVLSNFAFYNCENVLPN--QFPVGRDANDEN-------------VVNRV--SGV-----------LNNRMPFCLDFEDFNSQHSS-GNMKA-VIYA--YIETFIDCL---------------------------TPEQ---------------------EQAAM-WTAASLDKQIINDN-VGTKTTYESKGTLLSGWRLTTFMNSVLNYIYTTKLAAEEK------RPGDSLHN-GDDVLIGV--------RSMALPQRCMQNAIKYNVRMQSSKCAVGAI-AEFLRIDHK----Q-GGNGQY-LSRAVATMVHSR-IESRVSTDIRD---------------------------------------------LVQSMENRFADAKGRGMA---------NDIISG---LREQYYMRQSVLCDTD---VEDIYLIKNAHRVVGGISEEKD--------SKMGVL------ITSQLRAQK------------------------NVSIPY-L--------------PGVNEYANEIHK--ALKIN-----------VSIKTICDRL-------------------YDATYEAVSIKDRKMKI------------------------------------------------------------------------------------------------------------LRENRDQWFV------------------------------NVRRIYKAH----------------------------------------------------KGSQLSQNY------------GKA---------------ALVGFALEVLGR----------------EMPDATITTILNTS----------------------------------------------QRPLDLIKHIL-----------------------------------------------

>refseqp|YP_001497151|814_AA|Black_raspberry_virus_F_Totivirus

M-----------------------------------------------------------------------------------------------------------NIPVWVTIRGECL------------------------------TAVDREKAKYVLAHVSYSNTQDSPGFGMFR--------------------------------------------------------VGKECVKGFM-ARCEDIWVYYVKTST-------------------------------------PAISLPPVVK------------------------------------------------------------------------------------------------------------------------------------------------------------------------------------------------RTMSAMYSGVE---------------DYDFSDFRGHR----FLRRKFG--------------------------------------VVREEIIHAKNIKPPQK----------------------GEFEKS-------------------------------------------------------------------------------------------------------------KITGEHHTHYTPDE-VWEIAVQTN------TMRRALLLVVDKLREISGVTEAIV-------------------------------------------STFMAYIMFAKP-----QVAY------LLAT------SANIWR----------------AKDVNTLAEKLKEISTPLKSMHRH------DVLDMTQ----LFELQVLV---NRGIGKVDWVNE-----RKHRQKPDTVK----------------VG-FTDVYCEARRLFELGVQ--------RGFKYPTMDFDKYLKSRWEWVPTGSVHSQYEGDQEY------------------------IAK-DYRHRSKFVTLNRLSCDQIREMFNRKPEI-------QAWASVKY-EWA--KQRAIYGVDLTSSVITNYAMFRCEDVLRH--HFPIGSEADAQR-------------VHKRL--TYM-----------LKDTESFCYDFDDFNAQHSI-ESMEA-VLLA--YYDQFKDQM---------------------------TAAQ---------------------ASAME-WVCQSVKEMIVNNNEVQPPEKYKLKGTLLSGWRLTTFMNTVLNYIYFRISGALKTP-----DVVDSVHN-GDDVLLSI--------NNLKAAVEVDSRMAKIGARAQAAKCNVFSI-GEFLRVDHKISREK-GVGAQY-LSRAAATLVHSR-IESQAPLRLIE---------------------------------------------AVKAAYTRASEVAMRSDQS--------SEISAL---FFHKAAKRLSSIFGVD---YEKVRIAANQHVVTGGIRQDFM--------ASVEYL------IHEEVEREKLGTD-----SLHNLG---------QVDVSDLG--------------EGIKDYGSVLMS--QYGKY-----------TTEERIYKSI-------------------VSATGRQLSITRSTRLS-----------------------------------------------------------------------------------------------------------VVDVRHDTKYG------------------------------YGRALYGYL----------------------------------------------------RNLVNLPYV------------EKA---------------RFVGISPLAMLD----------------AKGMGTLRKYIMGA----------------------------------------------SDVDYTLRVLL-----------------------------------------------

>refseqp|YP_009259484|1608_AA|Maize-associated_totivirus_2_Unclassified_Totivirus

MLNAT-----------------------P-----------------------------------------GGDGPYIKRKEPLTL----------------------NNIPVWVNIRHNHL------------------------------TAVDRHDAKYVLGFILPGQSFEVGGFGRYL--------------------------------------------------------VGKTCVAAVA-IPCDGMWLLYVSVTT-------------------------------------PANILPPIVK------------------------------------------------------------------------------------------------------------------------------------------------------------------------------------------------RTLSAMFSSVD---------------GYYFHDADTTK----YLRKTFD--------------------------------------VERSAIAHAAKPIPPRE----------------------GEFERA-------------------------------------------------------------------------------------------------------------VITAEHHTHYRPEE-IWAIAEQHT------TTLRAMGLVLANLRKIQGITEATV-------------------------------------------ATFLAYIMTVRP-----QVAY------LVAT------SKRIWR----------------SKNITELTEILKEIATPIKSMHQH------EICDLTE----LFELQCLV---NRGVGQIDWRKE-----RSHRTRPDVVK----------------VS-IEDVVKYATEIFLLGKS--------HGYHYRKMDKQKYISARWEWSPTGSIHSQYPEDEPY------------------------IPK-NYRQKTKFVALNMMSRQHVDSMFLRKPEI-------HAWTSVKY-EWA--KQRAIYGVDLTSTVITNFAMYRCEEVFKH--RFPVGEEAAADR-------------VHRRL--KIM-----------LEDNESFCYDFDDFNAQHST-QAMQA-VLVA--YFNVFQADM---------------------------SDDQ---------------------REAML-WVIDSLSDVTIHNSNVQPPEQYELKGTLLSGWRLTTFMNTALNYVYFKAAGCFDIG-----GVRDSVDN-GDDVLVSI--------KHIGAAVRIHHRMALINARAQPTKCNVFSV-GEFLRVEHKVDKDT-GLGAQY-LTRACATLVHSR-CESQEPEHLTG---------------------------------------------AVKAIVTRAREVFERAKIS--------QALLAD---LVRSAIRRAATIFHRP---AKEAFIIAELHAVVGGASTDDF--------APIDFK------INERCEYDKERC------ANDKND---------MVVTQDLY--------------PGIFDYARTLAQ--AYEGV-----------LDEQQAKRRV-------------------ISATTRQISVTRKTWLD-----------------------------------------------------------------------------------------------------------IVPLAADTFYR------------------------------YGRALYKMY----------------------------------------------------HGLVSMPHL------------EKA---------------RFVGIPPIALLD----------------SRSRNMIRNIIVEA----------------------------------------------SDVEYALRVLL-----------------------------------------------

>refseqp|YP_009448194|834_AA|Maize-associated_totivirus_3_Unclassified_Totivirus

MLNAT-----------------------P-----------------------------------------GGDGPYIRRKEPLTL----------------------NNVPVWVNIRRNHL------------------------------TAVDRHDAKHVLGFILPGESFDVGGFGRYL--------------------------------------------------------VGKACVAAIA-IPCDGMWLLYVSVTT-------------------------------------PANILPPIVK------------------------------------------------------------------------------------------------------------------------------------------------------------------------------------------------RTLSAMFSTVD---------------GYYFHDADTTK----YLRKAFD--------------------------------------VDRSVIAHAAKPIPPHE----------------------GEFERA-------------------------------------------------------------------------------------------------------------VITAEHHTHYRPEE-IWAIAKQHK------TTLKAMGIVLANLRKIQGVTEATV-------------------------------------------ATFLAYIMTVRP-----QVAY------LMAT------SKRIWR----------------SKNIAELTEVLKNIATPIKSMHQH------EICDLTE----LFELQCLV---NRGVGQIDWRKE-----RSHRTSPDVVK----------------VS-LADVVKYATEIFLLGKA--------HGYHYRKMDKQKYISARWEWSPTGSIHSQYPEDDPY------------------------IPG-NYRQKTKFVALNMMSRQHVDSMFLRKPEI-------HAWTSVKY-EWA--KQRAIYGVDLTSTVITNFAMYRCEEVFKH--RFPVGEEAAADR-------------VHRRL--KIM-----------LEDNESFCYDFDDFNAQHST-QAMQA-VLVA--YLNVFQADM---------------------------SDDQ---------------------RDAMS-WVIDSLSDVTIHNSNVQPPEQYELKGTLLSGWRLTTFMNTALNYVYFKAAGCFDIG-----GVRDSVHN-GDDVLVSI--------KHIGAAVKIHHRMALINARAQPTKCNVFSV-GEFLRVEHKVDKDT-GLGAQY-LTRACATLVHSR-CESQEPEHLTG---------------------------------------------AVKAIVTRAREVFERAKIS--------QALLAD---LVRSAIRRVAAVFHRP---AKEAFVIAELHAVVGGASTEDF--------APIDFK------INERCEYSQERS------ASDKDD---------MVVTQDLY--------------PGIFDYARTLAQ--AYEGV-----------LDEQQAKRRV-------------------ISATTRQISVTRKTWLD-----------------------------------------------------------------------------------------------------------IVPLPADTFYR------------------------------FGRALYKMY----------------------------------------------------HGLVSMPHL------------EKA---------------RFVGIPPIALLD----------------SRSRNIIRNIIVEA----------------------------------------------SDVEYALRVLL-----------------------------------------------

>refseqp|YP_009225665|822_AA|Panax_notoginseng_virus_A_Unclassified_Totivirus

M-----------------------------------------------------------------------------SKVPLTR----------------------NNIPAWCNIRHNNL------------------------------SACSREKAGYVLGHITYDIKSSVGGFGAHR--------------------------------------------------------VGNNSVRSVA-VKCEDFWLLYVKTTH-------------------------------------DATLLPPMIR------------------------------------------------------------------------------------------------------------------------------------------------------------------------------------------------RALSVAYSQVD---------------GYDYSDWQLFR----YLRREFK--------------------------------------VDRKMITHARVGTGPRP----------------------EEFPRA-------------------------------------------------------------------------------------------------------------TVTGEHHTHFRPEE-VWEVSKKYR------AKLRAMSVVYNNLKKIEGVTEAVV-------------------------------------------STMFLYILFARP-----QTAY------LFAC------SAKIWS----------------VRDINGLSDILKTISTPLKSVQNN------DLCDVTQ----LFELQCLV---NRGIGKVDWRKE-----RANREKPNVVI----------------VD-EKKVYHEAMNLFRMGIS--------HGYKYARMDLNKYVKSRWEWVPSGSVHSQYEEDEPY------------------------IKK-SNMHRTKFVTVNMLSRAHIRRIFARKKEI-------HAWASVKY-EWA--KQRAIYGVDLTSSIITNFAMFRCEEVFKH--RFPVGEEAAASR-------------VHKRL--KMM-----------LDGNESFCYDFDDFNAQHST-GSMYA-VLQA--YMDVFRGSM---------------------------SRDQ---------------------VSAME-WVRDSILSVKVHNHEEGREEFYKTDGTLLSGWRLTTFMNTALNYIYFKLAGAFDVT-----GVRDSVHN-GDDVLVAI--------RDIRSACSIHGAMARINARAQATKCNVFSV-GEFLRVEHKVTREE-GLGAQY-LTRAAATLAHSR-IESQEPTRLVD---------------------------------------------SIKGMVTRCEEITARSFGM--------ETVAYE---LLTHSVRRLCGIFGVQ---AEDGYKVLKGHTLVGGAMETFD--------GSIKYK------IREEVLIGKTVIT-----ADNRHE---------YATLKDMM--------------PGILDYAEILNN--QYGDY-----------IPIDNIKKKI-------------------IYATHRQLEVTRGTRLI-----------------------------------------------------------------------------------------------------------VTDVQHDDKYK------------------------------YGRALFRMY----------------------------------------------------KGLITIPYV------------EKA---------------RFLGIPPIAMLS----------------HSSMKFVTKLISSV----------------------------------------------KDVDYTLRTLL-----------------------------------------------

>refseqp|NP_620495|731_AA|Saccharomyces_cerevisiae_virus_L-A_Totivirus

M-------------------------------------------------------------------------------------------------------------------------------------------------------------------------------------------------------------------------------------------------------------------------------------------------------------TILSPAAR------------------------------------------------------------------------------------------------------------------------------------------------------------------------------------------------RQASATYSQVA---------------GFCYNTPTVMD----SLANILD--------------------------------------VDRNIRPKHFKGL--------------------------RLYTRS-------------------------------------------------------------------------------------------------------------KVTAQHHTHLRPDE-LVEAAAKVSP-----RRKYYLMCVVELLANLQVDLEAAV-------------------------------------------ATILAYVLTLSE-----KFVP------IFLD------SRAIWV---------------GEPGPDALTARLKASSGQIKSIHTA------DYEPLTE----LFELAVLM---NRGVGHVSWQAE-----KDHRLNPDVAV----------------VD-QARLYSCVRDMFEGSKQ---------TYKYPFMTWDDYTANRWEWVPGGSVHSQYEEDNDY------------------------IYP-GQYTRNKFITVNKMPKHKISRMIASPPEV-------RAWTSTKY-EWG--KQRAIYGTDLRSTLITNFAMFRCEDVLTH--KFPVGDQAEAAK-------------VHKRV--NMM-----------LDGASSFCFDYDDFNSQHSI-ASMYT-VLCA--FRDTFSRNM---------------------------SDEQ---------------------AEAMN-WVCESVRHMWVLDP--DTKEWYRLQGTLLSGWRLTTFMNTVLNWAYMKLAGVFDLD-----DVQDSVHN-GDDVMISL--------NRVSTAVRIMDAMHRINARAQPAKCNLFSI-SEFLRVEHGMSGGD-GLGAQY-LSRSCATLVHSR-IESNEPLSVVR---------------------------------------------VMEADQARLRDLANRTRV---------QSAVTA---IKEQLDKRVTKIFGVG---DDVVRDIHTAHRVCGGISTDTW--------APVETK------IITDNEAYEIPY---------------------EIDDPSFW--------------PGVNDYAYKVWK--NFGER-----------LEFNKIKDAV-------------------ARGSRSTIALKRKARIT-----------------------------------------------------------------------------------------------------------SKKNEFANKSE------------------------------WERTMYKAY----------------------------------------------------KGLAVSYYA------------NLS---------------KFMSIPPMANIE----------------FGQARYAMQAALDS----------------------------------------------SDPLRALQVIL-----------------------------------------------

>refseqp|YP_009507833|795_AA|Tuber_aestivum_virus_1_Totivirus

M------------------------------------------------------------------------------------------------------------------------------------------------------CAVSRHSSTHMLVDVIPCSQLGLGAV------------------------------------------------YYHTEVAVR---VYGEARYCRL-FRLDDVYGCYMRTDA-------------------------------------YTPCLGPVTK------------------------------------------------------------------------------------------------------------------------------------------------------------------------------------------------RLMSQVYSLVN---------------SVCFNSCDISDYLSLTLGSTLQ--------------------------------------MPKIEWP-------VKH----------------------GIYERS-------------------------------------------------------------------------------------------------------------KITATHHTHLRPQE-LEAVAEGMGG-----RTKFVYAVCLQRLLSIGVVTEAVY-------------------------------------------SSFLAYIIGVEY-----NFAL------LLLR------STTVWS---------------GTRGEEQLLSKLKSFASAIKASHSA------KYQPLTQ----LFELTVLL---NRGIGSVDWAGE-----KENRVNPNTVD----------------LD-PADVYKSSMTIFRKARK--------SGYKYPKMHYDKFIESRWEWIPGGSVHSQYVDDDEY------------------------IYP-GLYTRNKFITVNKMPSHHLQLFLSSTPQI-------KAWTSTKY-EWG--KQRAIYGTDLRSTIITNFAMYRCEEVLLH--LFPVGDQADVEK-------------VHRRI--SMM-----------LDGYDSFCFDYDDFNSQHSL-SSMQA-VLLA--YMDAFSDVM---------------------------SPEQ---------------------LQAME-WVILSVADMQALSP--TDNEWYRLTGTLLSGWRLTTFMNTVLNWVYMDVAGVFNID-----GVHDSVHN-GDDVMISI--------SSIKAANLVMNKMHKVNARAQPTKCNVLSI-SEFLRIEHGMSGYD-GLGAQY-LSRSCATLVHSR-VESKEPITATR---------------------------------------------LVEADLSRLKDYRSRCAN---------VDAVDL---IQQQLFCRTAKLFNIE---LGNIYKISELHRVVGGCSDRRW--------APIEQQ------VTTTSGKYEIPH---------------------EIDDPSFW--------------PGVNDYSAMLYR--KIGEQ-----------VKMSVIVDAV-------------------SQGSRLTIAKSRSVTLA-----------------------------------------------------------------------------------------------------------VERTANIKAKE------------------------------WERALYRTY----------------------------------------------------KGSTVHYYA------------NLA---------------KFLNVPPLFGIE----------------TRGGASAISAALAS----------------------------------------------SDTMRALKVLL-----------------------------------------------

>refseqp|YP_009508104|1114_AA|Aspergillus_fumigatus_chrysovirus

M----------------------------------------------------------------------TFGHTNNRSRPNNG-------------------ERISMMATQFDIVRGGRISKQLSKLRSMRAYSQSGERFFGQNKWSREQY----EHSMARYNELAALRHNLYA-------------VIMPA------------------------------GCGKSYLAKK---YGMVDVDELI-ARQEHDSYVDAR---------------------------------------------------GAIIVGKGDW------------------------------------------------------------------------------------KDHNNIWFSRLNETLD--------------LLDYSMPVIIFVHT---------------EETALE-----------IGAKPI------------AFLRLTETAH----------------------EMNIKHRDPQFRQWSRESL--------------------------------------KRCRVSGDVPNQMVFSSNRDLEAFFLSILNASGIPVGGPLQFSEAIWNDSYAHDVPGWILRGERLGDPTVSINQLRLLFNEGKVPKECVDFYVR-HSYVPTQFDFGVSM----------YEWSQALGQLPPCYNDH--VDFDTEGDMMKVFPPSSPKEITRANVRVRQ-LIQTFDIFS-----------HWDCYQIGAWHVGERQTFV-------------------------------------------SNLLCCWKGITQ-----FTGV----------------AALVF--------PWFRVCQKDWANKLKTLHSLVRCSRFLMNTEI-------SEKERQA----LMYMDLLV---GRSEYTIDEMSE-----VRLRASDTYETKHLSYDPDRKMFTNRKYK-EDFIVAVEEAYSRLRIK---------PKPVNVDSFMDFYQRRSTWLTKGSLVYNTLSPFMKKYYVQILDAVANTVLE------------IQGRHNKKSLFEVWEIGEVFQGVNET-------NFNITKAQIKY-EVGN-KDRTLLPGTLVHFIVFTYVLYLAEKQEQV--GSVRLNAANEVDIRY----------VDRKM----------------SEGIFHVLYDWANFNEQHSA-WEMGL-VIEK--LNSVIVA-----------------------------PRDY---------------------SFFVE-AIVAGMYNMGLHDR--EGKIH-KIWQGLYSGWRGTTWVNTVLNFCYVHVALVNMERLYGV-SVAIMLDHGGDDIVLGL--------SDPTYMPQFLETMDSMLFKANKWKQMFGVR-SEFFRNTIT----D-GSMYAS-PTRALASFVAGD-WEGAGRATVRE---------------------------------------------RVVSLLDQIAKLRRRGCS---------EELCQG---LTISTISHWCKVRDGEE-WLALPPVVIHGRVEDGGLGVPD---------RDNNVWVLKDK-VPEVNEEWY------------------------KVVVPD---------------YKASRDYVEVLAR--DLEKFCLV-------IERREELARKL-------------------AEDSYDIEKAVDHEQWR----------------------------------------------------TLLDFHTE----------------------------------------------------VVDKYE------------------------------ITPDVTDDVI-------------------------------FEGFVVYE------------VDEETERKF------------DAA---------------ARYQEFVSYL------------------TFNEKAISKEELVNIMSDGQ----------------VSLEALEFQGDIYYARLV------PEFISYRATLFC------------RDMINQGVLDITTAGHVFRVICSMAKYVFGHLA-

>refseqp|YP_009337840|1117_AA|Isaria_javanica_chrysovirus_1

M----------------------------------------------------------------------AFTKTQGYREPETT-------------------VSEDVLKNTMRGAVKAARRRNKPGLVSLAKYAPGQRTFLGQSK-SREEYEVKLANSMARYEQIRSKRQNLFA-------------FVMPA------------------------------GHGKSTLARK---YGFVDVDELI-TEREHDYYIEMR---------------------------------------------------TGIMCGRDTW------------------------------------------------------------------------------------TDHNSKWYPRLNQTLD--------------LLDYTMPVIIMLHT---------------EELALE-----------LGAIPI------------GYFKLEKNVF----------------------MSNIEKRDALSKHFSILSY--------------------------------------NESIASSKMRNQVMCSSNEMLERSLLEILNLSSMPVACPHKFSSAIWNNCYARTVPGWILRGERAGEKRVDINMLRQLFEVGDIPKECVDYYVK-HSYVPTQFDFGMTM----------FDWSKELAHLPPTFRDR--TEFDTEGDMIDIFPPQCAKELTRANVTIRQ-LRQTFDIFS-----------HPDALEIAEYHVGEPHVFV-------------------------------------------SSILSAWKGIIQ-----KFSV----------------AEIVK--------PWFKVSFRNWSDRLKSLHSLVRCSRFLMNTEI-------TEEERQG----LMYMDLLV---GRGEYKIDEMNE-----VRARYGDGYGTKHLSYDPVTKAYTNKQYK-KDFELALDAAHLRLKVT---------PRKVNVLSFLDFYERRRSWLTKGGLVNNTLPKGMKEFSSQIFDGVANTVEE------------IRGRHNKKSLFEMHELYDILAGVNGD-------NFNTTKTMIKY-EVGR-KDRTLLPGSLAHFIVFSYVLVLAEKQDQI--GSVRLNALADVDIRY----------FDRKM----------------STGTFHVLYDWADFNEQHSA-DEMAG-VIRK--LSETMPA-----------------------------GPDY---------------------PMFCE-ALVRGMYTMGLEDR--EGVVH-KIWTGLYSGWRGTTWINSTLNFCYVFIALENLRRITGS-EVVLMVDHGGDDLDLML--------SQPDVMPLFLEIMDNMLFKANKWKQMFGLR-SEFFRNTIS----G-ARVFAS-PTRALASFIAGD-WEGAGRSTVRE---------------------------------------------RVGSLLDQIGKLRRRGVA---------EAMCQG---FAMSTISHWCKVKEGEE-WLDLPDVVLHGCEEQNGLGIPD---------RNNEVWILEKP-VPQLEEGWY------------------------KVVVPD---------------CKASRDYVRVLSR--DVEQFSLV-------LERQEELARKL-------------------AEDSYDIEKTLDRVQWR----------------------------------------------------ELLSFDCK----------------------------------------------------VLGKRP------------------------------VIEVMEDQAV-------------------------------FESFLEFE------------PDEDMATKF------------NKA---------------ARFQEYCGNL------------------SFNGMPITKEELVDLMSDGE----------------VSVEAIEFTGDIYYQRLV------PEFIANRAVTFC------------RAAINGNYLTVKEADRCYKIVCWMASRVYRHMV-

>refseqp|YP_009507948|1108_AA|Verticillium_dahliae_chrysovirus_1

M----------------------------------------------------------------------DLRQRQERARLN--------------------------QGTTVRSATRAARHGSNHGVVPLIRQT---ENAFASSK-AGAEYQQRLESSMSRYRVLREQRSNLFA-------------IIMPA------------------------------GHGKTHYAKR---YGFVDVDDLV-APRLHNELVDMR---------------------------------------------------YAAMHGRSTW------------------------------------------------------------------------------------TKHNDRWVGAINKTLD--------------LFDYSEPVVVLLHH---------------EETALE-----------IGAIIL------------GGFRLKQTTF----------------------KLNISSRTADDRFFSETSY--------------------------------------RSFDDLVATPNKIDNLDNQNLERLLIKTMCVNNLAVACPHKYERN-QSQYYKQSCPEWVLQGKPPIDRDIDMAELVRLYDSGIIPRQAVDYYVN-LGYTTTSLDFGVTL----------NDWGPVMAQVADGIGEP--QDFDVNGDMVEIFPPREPKELSRANVTMRR-LDETFAIWE-----------HSDVYDMCSFHVGEPHVFV-------------------------------------------SGLVTAWKGLMV-----KLKY----------------AHLVA--------PWFCVSYDHWTKAMKAVHTLVRTSRFLMSTEI-------TEDERQS----LMYLDLLI---GRTTYVINEMAE-----VDKRGGDHYSSDHLSYDPRVQLFTKAQYR-EDFNTAIKTAYTRMRYT--------KQPRLRVQSFRDFYLRRKEWLTKGSLVYNHIPSSQKRTVVQALDSINNVIVE------------LEARHNKQSLFEEMDLKDFLKLVGDAK------DFNVTKTMVKY-ETGK-KDRTLLPGSLIHFFTMTYVLELAERLEQV--GSARLKALPDDDFIW----------FDRKM----------------VNGLYHVLYDWADFNEQHSA-DEMAA-VIAE--LEHTVEG-----------------------------PSDY---------------------AYFVQ-AVSSSMYDMQLQDR--EGKRH-KLWKGLFSGWRGTTWINTVLNFCYSNIALMNYQRLYGE-DPVVYVDQGGDDIDSAL--------DNAPAMGKFMAIMDNMLFNANAWKQMFSTR-TEFFRNTIT----S-GRAYAS-PTRALASFCAGD-WEGSGNMTMGE---------------------------------------------RVINILDQVGKLTRRGLD---------QEFGNG---LVLCALTHWCKLRKEDS-WVNMPAEVIHGQESQGGLGVPD---------WNGCVWELETD-VPCVKDTWM------------------------AVLKPS---------------KLSSNDYVEELAK--DLDALSLE-------LVRREELATRL-------------------AEDAYDVELKLDKLNWQ----------------------------------------------------TIIGFRTK----------------------------------------------------VLSKRP------------------------------VVEYRREDKL-------------------------------FDDFMHFR------------VSDEMIRKY------------TDA---------------ARFSELAGHV------------------CVNGRELSKTELVDIMGDGR----------------VRLEALDFKGNPHYRRLV------PDFMGRRATFFC------------KEAINSGAADEEVGQYVFETICYMAREVFGHSM-

>refseqp|YP_052858|1086_AA|Helminthosporium_victoriae_145S_virus

M----------------------------------------------------------------------QKRQQQERES-----------------------QGERRLARHIESSMEVI------------------RGDFSSSK-TDHDYTMRLENSMSRFSKRRI--DNLFA-------------IILPA------------------------------GTGKTYLAKK---YGFIDVDKCV-TYNEHVLLYHER---------------------------------------------------KKILEGERRW------------------------------------------------------------------------------------ADHNESWNSKVRGTLE--------------MLDYSRPVVILCHS---------------EEMAFE-----------IGATPM------------IAVLLREDAW----------------------RENIKDRTKLGKQFSELNR--------------------------------------MTVEKHTRKVQVCRAKSNDIVEKLVIRACNAYGLPVACPNKYTTE-SNPHYGLSCPEWVMTGDV---SKMDVNVLLSLVDDDEIPKECADYFFR-SQNMPASFGYGQSI----------GDWAEWMAKVRYASNDA--RDLDLTKDWMELFPYANDREKNRMNVGLKR-IMENTNLIN-----------DEEILDILRHHVGENHQFV-------------------------------------------TMLVCYWAGIGR-----FLPE----------------ADLLR--------PMMKVNFTWWKVVMKEFHSLVRINDYVMNTKIE------SEEHRQS----MMYLDCLL---GRRIFIADEDAE-----IKDGTGSSGGMTHMSYDPVVRRWSVEQYR-KDFMFALENLHIGMIEK---------PKDPGIESFSQFYARREEWMTKGSLVSNTIPREYLEYTVKIVDDVNNVVQE------------VTKRHNKRSLFECYDAINLMTDKFEL--------FNVTKAVEKLNENGH-KDRVLLPGGLLHYIVFAYVLRCAEAQEQL--GSLRLNAPPDDEMRY----------IDVKM----------------HAGLSKLLYDWANFNVQHSS-EELAT-VISF--LGKVVQS-----------------------------GNDY---------------------KEFCD-LIADAMFNMVLKKR--DGTLV-KLDKGLYSGWRGTTWDNTVLNGCYMGVAKLCFVRLYKY-DCALFADQGGDDVDQEF--------AQPEDAYRMLAVLDRMGFEATKSKQMIGRN-SEFFRVTIT----R-TGAYAS-PVRGLATFVAGN-WEGTGNVSVKE---------------------------------------------RVVSLVDMAWKLIRRGVD---------AAFMIT---LTEVAITHWAKIRKDLD-WMKTPQEVIHGAEECGGMGIPD---------KDGMVWIVEPP-IPDPENTM-------------------------EVSVPG---------------KLAAMDYVAVLDR--ELRSNNIS-------IEKWEMVAEKM-------------------AEGAFDVYK-------------------------------------------------------DGAFEVLLEYKGN----------------------------------------------------VVEKKP------------------------------VVVPKWDEMA-------------------------------FRVLMEFEG-----------SKKTVAKQM------------SKL---------------ERYESVLPYL------------------SVEGREVGKKAILEAMGITG-----------------NMDVLDFKGDVYYRRLV------GEPFGRLVTNFC------------KTGLFLDELGKEQAEDLFRTLCYMGSKLFDHHI-

>refseqp|YP_006390091|477_AA|Persimmon_cryptic_virus

M---------------------------------------------------------------------------------------------------------------ALRS-ITGY-------------------------------EFHDFQSSLELLNQTHIH------------------------------------------------------------------------------IVRRESGVTYHDEFA-------------------------------------LCELLVDNTR-----------------------------------------------------------------------------------------LYEQELEGWSRSYYTGEQHMKA--------------ILQYSLPNTPIQDID-------------VGCYQQA-----------MTNVQE--------------------------------------------------------RLSSLPI------------------------V-------------RAFDVLTQLDQ----------------------------VSFESS-----------------------------------------------------SAAGYDYTGAKGPKNE----G----------------------------------NHERAIRRAKAVLWS--------------------------------------------------------------------------------------------------------------------------------------------AIAQDGEGIEHVLRS------------------------------------------------------------------------------------------SVPDVGYTRTQLTDLS------------------------------------------------------------------------------------------------------------------EKT--KVRGVWGRAFHYILPEGTSADPLLQAFKE--GGTFYHIGQDPTVS-----------VPYIL--SDT----------AGKCAWLYALDWSSFDATVSR-FEIHA-AFDL--LKQRIEF-----------------------------PNFE---------------------TEQCYEICRQLFIHKKIAAP--DGKVY-WAHKGIPSGSYYTSIIGSIINRLRIEYIWIKLRG-----HGPTICYTQGDDSLCGD--------DERIEPERIADIANPIGWLINPAKTATTRY------------PEY-ITFLGR----------------------------------------------------------------------------------------------------------------------------------------TCYGGLNHRD---------LIRCLR---------------------------------------LLIYPE----------------YPVPSGAISAYR------------------------ANSI-------------------AEDCGGTSSILNDIARR--------------------------------------------------LTRKYGRV------------------------------------------------------SHEEVP------------------------------KELRVYRH----------------------------------------------------------------------------------------------------------------------------------------------------------------------------------------------------------------------------------------------

>refseqp|YP_002364401|481_AA|Raphanus_sativus_cryptic_virus_3

M--------------------------------------------------------------------------------------------------------------RRLRNPLAGY-------------------------------VYTGFISDLEETNQRHTN------------------------------------------------------------------------------VIRRETAVTYRDEFA-------------------------------------LQSILDLDYR-----------------------------------------------------------------------------------------QYEQHLQGWSRSYYTPEKHQEA--------------ILQYSYPDIPVQAIK-------------MDVYNDC-----------IAQVQN--------------------------------------------------------EFRSLPN------------------------V-------------RAYSVLTQLDL----------------------------VKYKSS-----------------------------------------------------SAAGYGYQGTKGNPGE----L----------------------------------NHARAISRAKAVLWS--------------------------------------------------------------------------------------------------------------------------------------------AIAADGEGIEHVIKT------------------------------------------------------------------------------------------STPDIGYTRTQLTDLT------------------------------------------------------------------------------------------------------------------EKT--KVRQVWGRAFHYILLEGLVADPFIQAVME--NNTFIHAGRDPTLS-----------VPQLL--SHI----------AETSDWIYSLDWKQFDATVSR-FEIES-AFTI--IKSKTIF-----------------------------PDRE---------------------TEDAFEITKQLFIHKKVAAP--DGKIY-FAHKGIPSGSYYTSLVGSIVNRLRIEYLYRLILG-----RGPKICHTLGDDSLVGD--------DELIVPSSFGLIANRIGWYFNIEKTEYSRI------------PEM-VSFLGR----------------------------------------------------------------------------------------------------------------------------------------SYKGGLNVRD---------LKRCLR---------------------------------------LLVFPE----------------YPVPSGRISAYR------------------------AKSI-------------------AEDCGGISDLLNRVAAR--------------------------------------------------LKRQYGLA------------------------------------------------------SEEEVP------------------------------IYFKRYLPFM--------------------------------------------------------------------------------------------------------------------------------------------------------------------------------------------------------------------------------------------

>refseqp|YP_001686786|479_AA|Rose_cryptic_virus_1

MEHRF------------------------------------------------------------------------------------------------------RGIPRGLIELEE------------------------------------------------------------------------IPT------------------------------------------------------RRLREECVIHIDAWS----------------------------------SQAIDAIVPLSLR--------------------------------------------------------------------------------------------NELDGWARSYYTLQAHVDS--------------LMQYDRPKLQPPT---------------NTAWNIT-----------TQYIRT--------------------------------------------------------EFARMKK--------------------------------------VTALSYLQLDQ----------------------------VKWVRS-----------------------------------------------------SAAGYGYTGRKSDG-----------------------------------------DNYIRARKTAFTLAE----------------------------------------------------------------------------------------------------KLNH------------------------------------------------------------------------------------------NRDYGPLALEDS----------------------------------------TPDVAFTRTQLCQIK------------------------------------------------------------------------------------------------------------------VKR--KIRNVWGEAFHYVLLEGLFADPLIQQFMR--IKSFYFIGEDPLLA-----------VPRLI--EEI----------LSEQDYIYMFDWSGFDASVQE-WELRF-AFGL--LESILIF-----------------------------PSSV---------------------EHQVWQFIIELFIYRKIAAP--NGKIY-LKTLGIPSGSCFTNIIGSIVNYVRIQYMFFRLT------REFVTAFTHGDDSLVGVPT------TQYVQMENFKPICDENLWTINIAKSAISRE----------------AEGVSF-LSRKVREMCHAR-------------------------------------------D--------------------------------------------------------------------------------------------------ELICLR---------------------------------------MLKFPE----------------YIVETGAMSTLR------------------------AFSI-------------------HKDAGIHSRYLYQIYKF--------------------------------------------------LLHRYG--------------------------------------------------------KADSLP------------------------------LNQQNWDPIE-------------------------------------YENL----------RVSYATQNY-------------------------------------------------------------------------------------------------------------------------------------------------------------------------------E

>refseqp|YP_009158913|1139_AA|Fusarium_oxysporum_f._sp._dianthi_mycovirus_1

M----------------------------------------------------------------------------------------------------------SAFNASYSHTTVHRQ-----------------------------ATLDYTKGDVEQLEMLPCWKDCA--------------AVVAPV------------------------------CHGKSTLAMQ---FGGYDADELV-ADE----GPHRE------------------------------------DDPEWAEYVSCMPT----LGGAF------------------------------------------------------------------------------------DEQKQIRANQIRFTRLLR--------------FFSVQERDYNLPVVY-----------IHTAEYAHL-----------LGLNII------------AIAELDLEAIAATSRFD--GMSSL---EQHHYLESIRKQQSANREYAQRHGFEAPQYYPTYTEMLYSMK------------------HKIETRFASELRPSFYDK-----------------------HVSPEP-------------------AGSQKMD-TILLR-----------------CARIMETNLYTRAEKAIAAR----T-----------------MFSQFGETAIDITHTIHNHTQWASVIHRVATPAYFANIRKYLATHDRYICPNSEDAVRDAFPLAAGTPKFAVCRIYDWLRFD---TTEYDGEAWSVQALVTQGVGDGTVWSYERLLSTLFYDKIISD-----QYPH----------------TGAVMG-----GLGLGFLAGVEFAKRGSECHNLVRITGTI------FGHEF-DESLIGL----VTYWNSLA---GRSPLEVDIEAE-----AEARAAMSAPKRYFDT----KLNRWSSELFDLKLHEAIVDGYSEATNTAY--EKIAQMAEWATDFDEFLKHRKQWVKSGSATGGPKTDLYLRVPAEYRDMVADITEEVAVGVNMALHKVARLRLNKAATFEFPEFVAIVKEALRD---Y--KPNSFTRYFTKK-EVGRANPRSLYPATLMHYVVCCFILTLAEKGSPV--HGSRQQATEDQQ------------RTDHWLW---V---------ETCDHVTALMLDYVSFNEQHER-AHLKG-LIGS--LKVWYSRYG-------------------------LLTPDI---------------------AWAIE-WIQESFDQIVLQV----GDKHYHFINGLLSGWRMTSFGNSLVNKAYLAVIREQVLEITKK-VVLNHEQSGGDDVMSLE--------LALANVHLVLRFGEAMGFSFKAIKQLVSKRYREFFRLFAT----R-EGVYGS-VCRILGSAASGQ-WSNSVIGTLVE-----------------PSV-------------------------KIASVMDVLAKVMRRADMP--------LSFAET---FSYCMYEKWARIGD-----KRMMLTMLHGTTATGGRGIPM---------ADGSMYELEGVEIMRPRESIVE-----------------------MVGVPY----------------DASIVAVREMVE--QAKKYVTA-----DGILPEKEVALTM-------------------ARKVFHGALAQSQGLGV----------------------------------------------------AQLAVPDDVEYY---------TQAPKVRKKLTQTAIRDA---------------------TYEFWP------------TFNRMN-VQLEAAKKASAKLAAIKQALN----------DKGYATALETIAVEAGVEP----------------------IKVRLREEW------------------------------SLYGFARMALTEDY--------------YNDVVWLATLCADT---------------------------------------------EQQMNYIASALTTDLW--------VMGMLHY----------------------------

>refseqp|YP_009353026|1077_AA|Botryosphaeria_dothidea_chrysovirus_1

M---------------------------------------------------------------------------------------------------------SATLGASYSGTPHGP------------------------------NVTRLTGRGTAGLRPLPAWRDMV--------------SIVLPS------------------------------CCGKSTLCSR---YSGLDLDDIV-ADSSDY-----------------------------------------DMDAELDEMLAMREA--GLIHGDK--------------------------------------------------------------------------------------HQLIKQNELMLTRARR--------------FFATVSPDSNLKVVY-----------CHTAEMAEA-----------LGLRVL------------AVYSLPDEVVAASQRMR--EADVV---VRGATMRLMREQREANWHYARRHDLEHVEV-KAYSTLVHSVGEVL---------------SRARVLQYTDQARDY----------------------VA--MAFNQP------------------------KEAELLNR-----------------SLAILRSRSQLPWLKAVAAR----Q-----------------LQLSLGASAPCEAHHEHNHPMWARIVHAVASA-VVEPALTYVPEWD-------EDTWRDQYPLGPGNAQFAMCNISDWVRMGGAAMADPDSWVWFKQICAHRGS------RYERVLCHLVFGDVVAY---------------VVR-----PEHKELLN-----RLPLGSLSDVDFAVMAKEIHNNVRVG--LNYLGVA-----LAPSDLAY----FTYFDCLA---GRLMGEEDIEAE-----IEDRTRLQKAKIFFED------GRWSQSAFDTRLGEAIQTAYDEVSANIG--KVLVEMSDQVETFEAFLKNRRRWVKPGSATGSPKADVYLRVPGDRIEQLEELTTEIS---GMTVLVLKRVRLNKSAVFEFPEFVDMVKQALED---Y--VPNSFTKYFFKK-EPSKVKSRALFPGHLIHYIMVSHILYLIEKGGPIRNSRIMADSTAQREDHWFW--------------------------RESQDWCVHLMLDYANFNETHAI-KHMQQ-TILG--TKAIFAKHN-------------------------RLSADL---------------------RKSIE-WVSESFEKIAIEHE---GDLI-IFTHGLLSGWRNTSFTNSILNRAYLSVLGQQVEMLVGF-HPFLNYQSGGDDVAAEG--------RTLYETAVTLRVGKVMGFEFKDIKQLIGYRYREFFRLFVS----E-DGVYGS-LCRMLGSALSGQ-WSNSILPKFVD-----------------PVA-------------------------KLSSVIEVARKAGRRARS---------ASFMEK---MAMCAFEKWATDGE-----LRFADHIVHGTRETGGLGIPN---------VYGDVYELDG--TFTPTENYE------------------------PVGMPT----------------DASGELAASMIE--DAQKFLPP-----EATLPHEVLATQM-------------------AAGAFTSSVAQNRGPGV----------------------------------------------------LRVGLARQ-------------------DRKLVRI-----------------------KRIRAEEFPATT-SALFQ--ERLIRGR-DWLRAHRQAGARYSALSQAVK---------HPYRRVLASEICKRVPGADPAVIYF-------------------------W------------KEM--------------LELYGCATYLLTED-------------------------------------------------------------------------------------------------------------------------------------

>refseqp|YP_009508065|523_AA|Cryptosporidium_parvum_virus_1

MKFVNIYEI-------------------------------------------------------------QRFDGQPTRHGIAPKKIF-----------------RSKYIPTGLVPRLKYW-------------------------------------------------------------------------------------------------------------------------------RDVPSRAEMSKR----------------------------------------IGKYFEDEFKFY-----------------------------------------------------------------------------------------PN------------------------------------------------------------EEKLNEA-----------VNIVQEK--------------------------------------------------------------WITH---------------------------------YGTSLNVTSVSESF--------------------------RTLPKS-----------------------------------------------------TSAGLPFKSG------------------------------------------------------------------------------------------------------------------------------------------------------------------------------------------------CTKYEARNKMMRLARSQWDRVSK----------------------------GSQLQVLP---------------------------------------------------------CRSGARCQLR---------------------------------------------------------------------------------------------------------------------KRGENKPRLIWAYPGYLSIIENQYLTAIKKV-----------PPPNFIGWSINWLDGG-KSLNRLLF-GD-----------KWTWQSIAQIDFSSFDATVRT-ELIFH-AFKI--LRSLFDLTR------------------------T---------------------------ENIMLDQLRHYFINTPILFY----DKIIVKNRGIPSGSAFTQIIGTIVNMIACQYASLRSRDYNL--RIPFSCWL-GDDSFLNFETAL----CRQEFEYDYLEKFKELGLNVSIDKTHYTTRFIDDFEVRFK----GIRPYVKF-LGKQIDIL------------------------------------DLTFHND-------------------------------------------------------------------------------------------------------LDKLDA---------------------------------------QMALPE----------------KEDLSAYETGVR------------------------LIGL-------------------VWAYGAHYDIYLRILKV---------------------------------------------------YLSLKLKPVFH-----------------VQQLL----------------------------SYSEKPER----------------------------TKRYLENFFSSM------KYQLNLDLDIYDLLTF----PKFWDV-----------------SNRYFGSKY------------ERLDFR----------------------------------------------------------------------------------------------------------------------------SHKIYG----------------------------

>refseqp|YP_007889821|746_AA|White_clover_cryptic_virus_2

MPHNSTR----------------------------------------------------------------------------------------------------NYLAERMIRTKREL------------------------------MTYQSKDRDPDAILEASQDQD-----------------------------------------------YRRHYDNARYDPSNE---VKYRILNKEY-STLVEAYRLRNDRKH---------------------------------QPYELHQPIPEDAA-----------------PIPKSRVPAPGL-KLVPLMYHYGHVVHEPAHSDESDSDDNSDAPTRPVKESVPHFGYPVSKKIFDLIVRVYPEYIKVINTYCRPLGTVEATFAD--------------FNKEQIPSAPINSKRRET----------------------------VLKHIF--------------------------------------------------------KFLDTQP--------------------------------------YLPLHFVDTQY----------------------------CKTPLV-----------------------------------------------------TGTGYHNRYSFKQK-----------------------------------------AHAKYSHPEEYAKMH------------------------------------------------------------------------------------------------------------------------------------TSKGYFYNATYENARTLVHFIKQFGLPFNLQYAP------EDADLTD-DQVQSYIDAAN--------------------------------------------------NFFNDYPTLLFTRNHISKRD------------------------------------------------------------------------------------------------------------------GTL--KVRPVYAVDDIFIIIELMLTFPLTVQARKQ---------SCCIMYGLETIRG----SNHYI--ERL----------ARSYSTFFSLDWSSYDQRLPR-VITDI-YYTD-FLRSLIVINHGYQPTYEYPT-----------------YPDLDE---HKMYSR----------IDNLLYFLHTWYNNMTFVLS--DGYAYRRTHCGVPSGLYNTQYLDSFGNLFLIIDAMLEFGFSDAE-IDDFILLVLGDDNTGMTV--IPIDRIYDFINFLEKYALVRYNMVLSPTKSVLTTL------------RSK-IETLGY----------------------------------------------------------------------------------------------------------------------------------------ECNHGSPKRD---------ISKLIA---------------------------------------QLCYPE-----------------NGLKPHTMAAR------------------------AIGI-------------------AYAAAGQDFMFHSFCHD-------VYNIYRLDYKPDARTNLN--------------------------FQRQIYHNL----------------EDGIPDLATS--------------------------VVPPFP------------------------------SLYEVRHMYS------------------------------------------------QYQGPLTYAPKW------------NYA---------------HFINDPDDVPPNPKT----------MREYEIENDLISRTAPT------F--------------E--------------------------------TVVPATR---NFP-------------------------------------

>refseqp|YP_392480|709_AA|Rosellinia_necatrix_partitivirus_1-W8

MVLTIIR----------------------------------------------------------------------------------------------------DYLHEAQLRLKKEW------------------------------QTFQKSDQESGYSDKLPTDYD-----------------------------------------------LRRYYDSARDYDAEK---HKTEEYQHNF-ALTHERYTQMNADRN---------------------------------EPFEFYRPLEDNEL-------------------PDIRFPAPGI-TVLPFRYHTGQIV--------------------ETTDELPDSGFSLHPLIDYLTKTKWLHYRPYIDKYCRPLGTTNATFSD--------------FNREQIPSAPIDETRKNM----------------------------VLPLVI--------------------------------------------------------YFLNALP--------------------------------------FLPIHFVDTRF----------------------------CGTPKH-----------------------------------------------------TATGYFQRFSTFFR-----------------------------------------THAYYARNKLYALRP------------------------------------------------------------------------------------------------------------------------------------TSKGYFFNTVYEFSRTWMHHIKEHGYPFVPSHDA-----------------LDNARQYR--------------------------------------------------IFMQKHVTMLFTRNHISDRD------------------------------------------------------------------------------------------------------------------GFL--KQRPVYAVDDFFILCELMISFPLHVMARYP-----INGIKSCIMYSFETIRG----SNRYL--DSI----------ARDFISFFTIDWSSFDQRVPR-VITDI-FWTD-FLRQLIVINHGYQPTYEYPA-----------------YPDLSE---HDLYKR----------MNNLLHFLHTWYNNMVFVTA--DGFAYLRSAAGVPSGLLNTQYLDSFCNLFLIIDGLFEFGFTQAE-ILSIVFFIMGDDNSGFTM--MDIERLTQFIEFFESYALKRYNMVLSKTKSVITTL------------RSR-IETLSY----------------------------------------------------------------------------------------------------------------------------------------QCNGGNPKRP---------LGKLIA---------------------------------------QLCYPE-----------------HGPKDKYMSAR------------------------AIGI-------------------AYAAAAMDEEFHEFCRD-------IYHTFLPYAAPIDEHTLS-----------------------MATKHLPGYFKM-------------------LDNIASE-------------------------IKFDSFP------------------------------TLEMVQDKYS------------------------------------------------RWQGYLSHKPKW------------NDA---------------HFKFLPETVPNNIKT----------MTDYQLEHKLDTPVPHSLF------------------------------------------------------------------------------------------------------

>refseqp|NP_624349|673_AA|Fusarium_poae_virus_1

M-LANIR----------------------------------------------------------------------------------------------------DYFHEKLTRLLYDH------------------------------KIFQSNSKDPDLTLEAHHSSD-----------------------------------------------IERIYKSIHYDFNRSPAPIDYEAQYQSI-KHILEDKQSQQGFPH---------------------------------EYYRLHE-----------------------SPIPDDRIPPSGI-KLLPFEYKSMNVV--------------------TATPEVPESGFKIHPRIERLLRSKYPQYLQYVRKYTRPLGTTNATVSD--------------FFKPQTPSQPVEPTRINH----------------------------VMSHVM--------------------------------------------------------KKMAITP--------------------------------------YLPLHFVDTQY----------------------------DKRPLA-----------------------------------------------------NGTGYHNRRSHEMN-----------------------------------------IHALFSHPKEYESKR------------------------------------------------------------------------------------------------------------------------------------TSKGYYVNAFLESARSLIHWIKLYGNPFRHCPSD-------------------LAQSLR--------------------------------------------------EFFLQRPTMLFTRNHISDRD------------------------------------------------------------------------------------------------------------------GIL--KQRPVYAVDDLFLTIESMLTFPAHVIARKP---------ECCIMYGLETIRG----SNQIL--DKI----------ASDYKSFFTIDWSGFDQRLPW-VIVKL-FFTE-YIPRLLVVNHGYAPTYEYPS-----------------YPDLTT---NDMVSR----------LTNLITFLATWYFNMVFVTA--DGFSYVREHAGVPSGMLNTQFLDSFGNLFLLIDGLIEFGSTDAE-IDDILLFIMGDDNSAFTT--WSITHLEQFVSFFETYALSRYGMVLSKTKSIITTL------------RHK-IETLSY----------------------------------------------------------------------------------------------------------------------------------------QCNFGHPRRP---------IGKLVA---------------------------------------QLCFPE-----------------RGPRPKYMSAR------------------------AVGM-------------------AWASCGQDKTFHDFCRD-------VYHEFNDDRADLDESAYL-----------------------HIQSHLPGYLKI----------------DESVRQIV----------------------------DFQVFP------------------------------SQQTVYHTVS------------------------------------------------RWKGPLSYQPEM------------GSC---------------------------------------------------SLCQPT--------------------------------------------------------------------------------------------------------

>refseqp|YP_009408640|724_AA|Heterobasidion_partitivirus_7

MSTN-------------------------------------------------------------------------------------------------------PPIVEQLANPPQ----------------------------------PEDKHVQYAKLLD----------------------------------------------------------------------------------IYRANPSYYNQQ---------------------------------------QLKEYTELY--------GFNYFVPVPETDFPPERKPSNGLFALENFRFHVLPSF--------------------TRFRDIPRTGYNPLSFITYILYRMFPQLLNILDDYCRPAGNIDAIFEN--------------FNQEVTPVEDCDPARLIQ----------------------------IMTLIH--------------------------------------------------------FFMQIIP--------------------------------------FSPIAFPDLRF----------------------------YKWSLT-----------------------------------------------------TSADYHCHHSQDMKRES---KSYWQ---------------------------YLKDHLMLEERFDYSERP------------------------------------------------------------------------------------------------------------------------------------RSKGFFFNSVLLASRTIIHNIKYYGTPFKPTKGQ------DEP---------SRLSKLA--------------------------------------------------YWFMKYPTVLYVRSQISKI-------------------------------------------------------------------------------------------------------------------SKL--KIRPVYNAPFLFILLEAMVTLPLMAMCRLP---------GNCILWGFETIRGGMQELNRL----------------SFDFTTFIMIDWSRFDQLAPF-AIIYH-FWCT-FLPQLIRVDRGYMPQGEYTTSRHKSAFTAKHDNHFEHNPKYHSFATRLLTKYPPHVIMFTFVIFNILSFIWLWYAKMVFVTP--DGYGFIRLLAGVPSGIFMTQILDSFVNLFLFIDGLLEFGFSLDE-IRLIRLFIQGDDNIAFFI--GDFERIFAFYEWFPAYALKRWHMIISVDKSSITRL------------RKK-IEVLGY----------------------------------------------------------------------------------------------------------------------------------------TNNNGMPSRD---------LKKLVA---------------------------------------TLAYPERY-------------VTGPQWSVIQMSR------------------------AIGI-------------------AYANAAHDSSVHDLCRR-------AYNDARKHSGLTHDEL----------------------KNIKIEYQKLGFYEIFSV------------NLEELQSILVQ--------------------------DLSQFP------------------------------DYYDIRSNLR------------------------------------------------YWHGPHSVYPMW----------PS------------------HFDDPLSSIQE-----------------PDDLITLHTVLSHA------------------------------------------------------GVTFDR----NY--------------------------------------

>refseqp|YP_009362092|596_AA|Bipolaris_maydis_partitivirus_1

MLNNLV-----------------------------------------------------------------------------------------------------LGIKRGLAKAIRGN-------------------------------------------------------------------------------------------------------------------------AQDNH-VIEAYHHVVLHA----------------------------------------LNRFLPQHEV-------------------------------------------------------------------------------------------YEIVHGYRRSVFDENALNRD--------------IQKLNSEDHPVPKD---------------EHYWNA-----------IRKVQQ--------------------------------------------------------IFTPDVL--------------------------------------LQPVHFADLRH----------------------------YPWKLS-----------------------------------------------------TSIGAPFATSKEWNAYVVDKFHGYENG---------------FD-----------ESTFLKHYHRDLFAE--------AH-------------------------------------------------------------------------SGISL--------------------------------DPPMIDA----RMSKRNLYNEMFFINRKHIHIIKDGRKTNDS-------------------------------------------------------------------------------GHDLRYWHTAFARQHLVKQD------------------------------------------------------------------------------------------------------------------DPD--KVRLVFGAPSTSLMAELMFIWPIQAWLLS------L-KERSPMLWPFVTLTGGWHRLVNCF--QKF----------CPNFGLVASVDWSGFDRYARH-SVIRD-IHSN-IMRPMFDFSKGYHPTRDYPETQ-------------DTDPDR---------------------LENLWNWMCDSVLTTPLMLP--DGTLIRFQHSGIFSGYFQTQLLDSIYNLVMLFTIFSKMGFD----LDNIYAKVQGDDSIICIVCSFLMV-SHWFITMLKHYANYYFGAIVNDKKTEVSDT------------LEH-VEVLRY----------------------------------------------------------------------------------------------------------------------------------------RNRGGIPYRE---------RIELLA---------------------------------------QLYHPE-----------------RAITYQALMAR------------------------SVGI-------------------AYANCGSDPRVYQICEN-------IHQYLSKL-GVKPDP--------------------------------AGLPSGVRF----------------VQDYLPG----------------------QTAIDVQRFP------------------------------SYFETVSRLL----------------------------------------------DGY--EEQPSESYW------------PRS---------------HFIGIP----GRT-------------------------------------------------------------------------------------------------------------------------------------

>refseqp|YP_009508048|580_AA|Flammulina_velutipes_browning_virus

M---------------------------------------------------------------------------------------------------------SDTLIDSFSR-------------------------------------LTLSVKNFVFLGFTETQ---------------------------------------------------------------------NYPQKSDS-AILSHRKVVLNA----------------------------------------FEKYLNPIEY-------------------------------------------------------------------------------------------NHVANEYKRSETDLDSTKAA--------------FFKGDIPDHEVPRD---------------EHYNRA-----------FSVIVS--------------------------------------------------------KFRPPEP--------------------------------------IRPVHYADLRL----------------------------YPWPLK-----------------------------------------------------PSAEAPFSNDKSLLA-------------------------------------------LLALRNRQGFLP-----------------------------------------------------------------------------------------------------------------------------NA----KPNFHNLFNWVFGFNRQCVHLIKKGKDNLGPNEYW------------------------------------------P--------------------------------AHGFLYPINIHTKSAIIGIH------------------------------------------------------------------------------------------------------------------DPN--KVRTIFGVPKLTVMVEAMFFWPLFRYYRF--------EQQSPLLWGYETMLGGWYKLNHELH------------LNPFYQGSILSLDWSFFDGRALF-SVIND-LYSDKGVKSYFEFNNGYIPTVDYPDSS--------------THPQK---------------------LHNLWDWMLTALKFAPCALA--DGTIWQRTVRGIASGQFTTQFMDSIYNGLMILTILSRMGFVI---DETLPIKLLGDDSVTRLAVSIPASMHESFLIEFQRLADYYFSHTINVKKSKISNT------------PHN-VSVLSY----------------------------------------------------------------------------------------------------------------------------------------ANNNGLPVRS---------RTSLLC---------------------------------------ALLYPK----------------SRRPTWEHLKAR------------------------AIGV-------------------YYASCGIDRTVRLICKD-------IFDYLDSQ-GIQASS--------------------------------AGLQDLF------------------DPNFKSG------------------------TIPLDVFP------------------------------SVEQVTTNLR----------------------------------------------SFHHLDNSDKERYF------------PTS---------------HFLDTK--------------------------------------------------------------------------------------------------------------------------------------------

>refseqp|YP_009508049|621_AA|Heterobasidion_partitivirus_1

M----------------------------------------------------------------------------------------------------------DYLTGLFSRVLH----------------------------------ISRKVTNFEFAGTYHYQ----------------------P----------------------------------------------SIPQVNEV-ATENHKRTLRHS----------------------------------------FRTYLTSDEY-------------------------------------------------------------------------------------------DKIVNGYKRTNLDPSTITED--------------FFSGDIEDHPEPTDFKSQ-----------LSIEYG-----------LQCMID--------------------------------------------------------AFKPPAP--------------------------------------ARVCHLYDVQWH---------------------------YPFKWQ-----------------------------------------------------VNAEAPFSTEKYFLDL----RKKFGDF---------------FDPVTKLWTKYVNPLDALRRYGHTPPAD-TLNQVTP----------------------------------------------------------------------------------------------------------------------------PKFGFMKNLIFSFVHSWQHVIKSRFTSNAGITHS-NFL-----------------------------------------------------------------------RQRFLFPMLLHIKTAIVSFD------------------------------------------------------------------------------------------------------------------APN--KLRSIWGVSKLWIISEAMIYWEYIAWIKL-------NPGSTPMLWGYETFTGGWFRLWRDLH-------------TPGEDVTYITIDWSRFDKRAYF-WLIRK-IFIR--TRCFLDFTNGYVSTKDYPTSP--------------TDPDK---------------------LQALWEWTIEAFFDSPIVLP--DGSMFKRLFAGIPSGLFITQLMDSWYNYTMLAAILHYMGYD----PRRCIIKVQGDDSIIRLYIQIPLHEHDLFLLRMQEVSDHLFGAKISFEKSELRNS------------LIG-SEVLSY----------------------------------------------------------------------------------------------------------------------------------------RNIQGLPYRD---------LIKMLA---------------------------------------QFYHTK----------------AKDPTPEITMAQ------------------------AIGF-------------------AYAACGNDFRIHELLRS-------VYDYYKAQ-GFTPNP--------------------------------AGLTVVFGD----------------SPDRPDY------------------------PISLDEFP------------------------------SQMDVQRFFL----------------------------------------------STDYRNADQENRTW------------PSS---------------HFLYAPCSRI----------------------------------------------------------------------------------------------------------------------------------------

>refseqp|YP_009011230|623_AA|Rhizoctonia_solani_dsRNA_virus_2

M-------------------------------------------------------------------------------------------------------NLYNRVSALFAN-------------------------------------WFSSPSNLEFVGSYHHQ----------------------P----------------------------------------------GTVPPNPS-TQEAHKRFLHNV----------------------------------------FKQHLFTYEL-------------------------------------------------------------------------------------------DYIENEHRRSEATPEAIEND--------------FFANDVEPHDIPFD---------------VHVEIG-----------LQCMTD--------------------------------------------------------AFRPPVP--------------------------------------CLPAHLNDVEHH---------------------------YPFKWQ-----------------------------------------------------VNAEPPFSTDSYFLDN----RKLFSDY---------------YDTESQTWRGYVDPFEANRRYQHTKDKEGFLNQTVP----------------------------------------------------------------------------------------------------------------------------AKFGFMKDTIFSWTRRWHHIIKEGFQTATNLSST-AYL-----------------------------------------------------------------------RDRFIFPMLLHTKTAIVKKD------------------------------------------------------------------------------------------------------------------DPD--KMRTIWGCSKPWIIADTMFYWEYIAWIKK-------HPGVTPMLWGYETMTGGWMRLNSQLF-------------SSYLKKSYVTLDWSRFDKRAYF-KLILA-IMCR--VRTFLDFDNGYLPNVNYPDTRT---------DWSPNKAQR---------------------LERLWLWTLECLIKSPIVLP--DGRMYIRHYAGIPSGLFITQLLDSWYNYTMICTILSSIGLN----PKHCIIKVQGDDSIVRLGVLIPPEAHEAFLLALQSKADFYFKAIISVDKSELGNS------------LNN-REVLSY----------------------------------------------------------------------------------------------------------------------------------------RNYNGLPRRD---------EIKMLA---------------------------------------QFYHTK----------------ARNPTPEIAMAQ------------------------AVGF-------------------AYASCGTHQRVLDALEH-------VYTDYKDA-GYTPNR--------------------------------AGLSLVFGN----------------SPDIQLP------------------------HYDIDHFP------------------------------SIEEIKRFLT----------------------------------------------CNSYDNSVQMAKSW------------PTS---------------YFISEPCERM----------------------------------------------------------------------------------------------------------------------------------------

>refseqp|YP_086754|616_AA|White_clover_cryptic_virus_1

M----------------------------------------------------------------------------------------------------------DYLITAFNRITH----------------------------------WFLTPTNLEYIGSYSLP----------------------P----------------------------------------------GLLRVNDV-AVANHKATLDRS----------------------------------------FDKYLYEHEI-------------------------------------------------------------------------------------------NLITKEYRRSPIDEDSILED--------------FFSGDLPYFEIPFD---------------EHVERG-----------LECMAA--------------------------------------------------------AFRPPRP--------------------------------------CRPAHILDVKHG---------------------------YPYKWN-----------------------------------------------------VNAEPPFSTDEYFLSQ----RKTFGEF---------------IRMHEYE---HIDKEDFFRRHPNIESHD-FLRTVVP----------------------------------------------------------------------------------------------------------------------------PKFGFLKSMIFSWTRRWHHIIKSGFQDSTDLEQT-GYF-----------------------------------------------------------------------FNRFIFPMLLHTKTAIVKKN------------------------------------------------------------------------------------------------------------------DPN--KMRTIWGASKPWIIAETMFYWEYLAWIKH-------NPGATPMLWGYETFTGGWFRLNHELF-------------CGLIQRSFLTLDWSRFDKRAYF-PLLRR-ILYT--VKTFLTFEEGYVPTHAAPTHP----------QWSQENIDR---------------------LERLWLWTLENLFEAPIILP--DGRMYRRHFAGIPSGLFITQLLDSWYNYTMLATILSALHFD----PLHCIIKVQGDDSILRLTTLIPVDQHTNFMDHIVRLADTYFNSIVNVKKSEVRNS------------LNG-CEVLSY----------------------------------------------------------------------------------------------------------------------------------------RNHNGLPHRD---------EITMLA---------------------------------------QFYHTK----------------ARDPTPEITMAQ------------------------AIGF-------------------AYASCANHNRVLWVLED-------VYNYYRDL-GYRPNR--------------------------------AGLTLTFGD----------------SPDLTMP------------------------EMPLDHFP------------------------------TKSEIRRYHT----------------------------------------------ETHYQNEAQNARTW------------PRT---------------LFINAPGE------------------------------------------------------------------------------------------------------------------------------------------

>refseqp|YP_009329886|582_AA|Rhizoctonia_solani_dsRNA_virus_3

M-----------------------------------------------------------------------------------------------------FYTTISTIKVFLKNLLFSD--------------------------------PHKFVNNFQFIGYASDRI------------------------------------------------------------------RVSIPYRDEF-QYERYQRTVRHA----------------------------------------LRRNLIGYDA-------------------------------------------------------------------------------------------EYIIKEFHHPVANLDFMVDA--------------LRKGDLPDHVIPKD---------------EHYSKA-----------FAQAAE--------------------------------------------------------MFRPPQL--------------------------------------VRPVHFADLRM----------------------------YKWNWH-----------------------------------------------------PNVEEPFYSDADLIR-------------------------------------------AVSMAAEAGLLP-----------------------------------------------------------------------------------------------------------------------------DA----RMSFGNLRNVVFIKARLFLHQIKRKQITN---------------------------------------------------------------------------------PATLWPMMKIHVKPALTKV-------------------------------------------------------------------------------------------------------------------DET--KVRIIYGVSKLHVMAQAMFLWPLFNYYIN--------SDDDPLLWGFETILGGMQKLHNIMS------------IPRLYFQTFVTVDWSGFDLRSVF-SLQRE-VFDV--WRTYFDFNNGYIPTKFYRTSV--------------ADPDH---------------------LEALWEWQREACFKMPFVMP--DRTMYNRLFRCIPSGLFSTQFLDSHVNLVMILTILDAMHFD----ISKIKIYVQGDDSIVMLIFHIPADQHIKFKSDFEVLAKYYFDHVARPEKTDVYET------------PQG-VEVLGY----------------------------------------------------------------------------------------------------------------------------------------RNYNGYPERD---------WRKLLA---------------------------------------QLLHPR-----------------GALSLETLAAR------------------------CCGI-------------------AYASMYRNPEVINVCKD-------IYNYLTTKRNVVPGELRA------------------------------------------------------QRDIILF-------------------GEHEFSIPTDHFP------------------------------ERDEVTRHLR----------------------------------------------IPYVRTDSDKNDYW------------PSG---------------HFLSLY--------------------------------------------------------------------------------------------------------------------------------------------

>refseqp|YP_009508053|581_AA|Heterobasidion_partitivirus_13

M-----------------------------------------------------------------------------------------------------LPIISQVTDYVYRKFIKPA--------------------------------PTQFKNNYLFQNWLSPL---------------------------------------------------------------------TTPHRDAT-KYAEYQAYLEKH----------------------------------------IRSNLLGSDA-------------------------------------------------------------------------------------------EYVIKRFHHPIATIDSVNET--------------LQRGDLPDHPVPKD---------------EHYYRA-----------LTETTK--------------------------------------------------------RFAPPQL--------------------------------------IRPIHFADLRY----------------------------YEWNWH-----------------------------------------------------PNVEEPYVSNSQLKT-------------------------------------------AVQDAYHAGLLE-----------------------------------------------------------------------------------------------------------------------------DG----RMSFGNLKNHVFMDVRHFLHRIKRGQISD---------------------------------------------------------------------------------PHTLWPLINMHVKPALTET-------------------------------------------------------------------------------------------------------------------DTT--KIRLVFGVSKRHVLPRAMFFWPLFRYYLD-------NRDKSPMLWGFETILGGMMLLNSEML------------LSRLYYQTFVTVDWSGFDLRSLF-SIIREDIFPA--WRTYFDFNNGYMPTKFYKSST--------------ADPDQ---------------------LERLWNWTNEAVFKMPFRTM--DGATFLRLFRGIPSGLFETQFLDSFYNMLMILTILDAMGFD----ISTIYIRVQGDDSLLLLTFFLPADQHAEFKAQFEALAAYYFDHVARQDKTDISNT------------SQN-VAVLGY----------------------------------------------------------------------------------------------------------------------------------------SNDNGYPSRD---------WRKLLA---------------------------------------QLFHPR----------------SQRPTLSLLKAR------------------------CCGI-------------------QYASMYKYPQVTNVAKA-------TFNQLDSE-GVQPVKLAA------------------------------------------------------QRDVILH-------------------SHKDFYVPTDHFP------------------------------TLNEVTRYLR----------------------------------------------IPYTRTEADSETYY------------PMS---------------HFLSQF--------------------------------------------------------------------------------------------------------------------------------------------

>refseqp|YP_003082248|580_AA|Sclerotinia_sclerotiorum_partitivirus_S

MPALNLR------------------------------------------------------------------------------------------------------------------------------------------------YLFSISRSEMKKIRSSHTK--------------------VSPR------------------------------------------------------EEATRFSILKHA----------------------------------------ILKHGSVGLL-------------------------------------------------------------------------------------------NQVLLGKRRSDASDERLIQD--------------FHEFEQPVHPVPRD---------------KHYLRA-----------LRVTEK--------------------------------------------------------LMKPAKT--------------------------------------LHPISFPDLRY----------------------------YPWTKN-----------------------------------------------------VSAEAPYNFEKKYEE-------------------------------------------LLRDKQRLGEIE-----------------------------------------------------------------------------------------------------------------------------TA----TATFHNLEDEIFEDNRYLIHKIKEGDSQF------------------------------------------WDKDG----------------------------------KPRPYYHTTLHARAHVVGHE------------------------------------------------------------------------------------------------------------------DAD--KIRAVFGVPKLLLMAENMFIWPLQAYYLN------QDTTKHHLLWGNEIMKGGWKKLWGQLQ-------------NGRISRTILSLDWSEFDKRALH-EVIDD-VHSM--WKSWFDFTH-YEPTIFYEKGEI------------PEPHRR---------------------IENLWIWMTDMVKHYPILQP--DGKVYQWTRNGIASGFQQTQLLDSFVNMIMLLTVLSANGINIE--HPDFWIKVQGDDSLISIVERRFQMFGISYLDTLADLASYYFNAKLSVKKSFISDT------------PQG-QYVLGY----------------------------------------------------------------------------------------------------------------------------------------FNHYGIPYRL---------DDDLLS---------------------------------------HLVFPE-----------------RPQRLEETAAS------------------------CVGI-------------------AMASMGCSKVVYSICDD-------AYTFITKT-LRRPAKAGSL----------------FW------LERAFGYEMP----------------------------------------------------NIAKMP------------------------------TFEECLYASY----------------------------------------------DIPVRTENMKQRLW------------PTN----------------------------------------------------------------------------------------------------AKAKNGFYFLRHLC-----------------------------------------------

>refseqp|NP_620070|1870_AA|Giardia_lamblia_virus_Giardiavirus

----------------------------P--------------------------------------FIRSCDGDRQLR----------------------------GNMGSYTGKTRTGF------------VYTYFSPNFLSSFN-----------------------------------------------------------------------------------VSEP---IFNTSINLTP-PYDDTSQAVIQN-------------------LSM-------PQML-----S-FDPYYESTFY---VVSADN--------EWIPTSGPAW-----------------------------------------------------------KVPYLENVVKRSGRRLLAELRIASN--------------NGSGDRTFLTTCKTR---------KGRHFTYFSAA--------------------------LGGKILEFVCAPLSSISLQG--GQT----------------------IYAPIQL-------------------------QD-----------VIPVRKEDPVPGSIYAV-F--------------------KFFSEP--------------------------------------------------------------------------KAW--------------------------------EARALKSYKVRFQD-LPSHIVISE----------LKERAARSYIGSRGYVDTGF-------------------------------------------KALDIYIDILSQ----MELPKYI-HEFLILLRGKVCEVSRLYKKEQVFV---------------ILLTVFSELTAIVRHRGNK------STGSMGRMWTLLSDFETLL---GKVSYK------N-PSIIEEQVVPWLTS-----DPI---PRTPDFY-STYFKTAVQFMHRTFVP-------VTLRSAPPLTFHEYCGRPELWGTTGSGYIGY----------------------------------GKRSFNKWSIYGAYPTEEIYRLALYGDNP-------PLKPLEKP-ELT--KVRAVISASLQSYILMSYLEYIMADTIVD--KAFTTTLMNDRQLEN----------LERHMM--------------TMTGGVRVPVDQSNFDRQPDL-VQIGI-WQQL--LFHLASASAPYR------------------------ARDS-----------------VSLVISRL--ASTTTFPNLKVRMS--DGDK--RVLHGLPSGWKWTALLGALINVTQLLTMAELSNTL----ASLRSTVVQGDDIALSM--------TDREQATQLVDTYARQGFEVNPKKFWISPDRDEFLRRVAT----P-GIVAGY-PARMMIKLLYQL-TEPEEPSHYISMLPKLAKVPNVVQEHVKPRSDVPWSETVEELDWREALAILRHRPARTSELVTQWLQLIGRFTAAH-----PDKRALTL---LYRWFVRDLTHATKIK---KRNLLVLLQQPGFWGGYSQSLVGLLRTTHLADMIVSELDIG-LPGPRATSSRFGTSPTSLAPHYL----------TLIVPSSVHV-QSQKELDLWGLCRSSKYAKHYSN--IFRYYK-L------TLPTLVLWAQRLGDKHVTDFIRSVTLGSEIPKYDPHARLFTSNGVSVG---MLIRIRVRHFTHRVLRRET----------------------PKCIPV---IVGLYRE--------------------QTLS---VPES---------------------VRLSEP----DKIL-------------LSLQRASGYSLNLVKKILQ-----------------------------------ITRKPVDHP-------VDARFSQAF------------PNN-------------------------------------------WSDLARTAGTFLLTVPA-----------------------------------------------------------------------------------------------------

>UrV2_RDRP

---------------------------------------------------------------------------------------------------------------------------------------------------------------------------------------------------------------------------------------------------------------QKHPH----------------------------------------YDAAVRAAIT-----------------------------------------------------------------------------------------------------------------------------LLLTSFPVQAPMSSI-------------------------------------------------DIIGLARLAFPVGTY----SRSPHSPP-----------------TNLPLRA-------------------------------------------ILKTPFLM--------------------------QYFPFK------------------------------------------------------------------------------------------------------------YHPAATMKTNVRMSD-LLRSLASQG-------ILARFETMIYALA--GRVSDDQA-------------------------------------------CSAILYASGLAP-----HLGP---YAYEF--------ASCCV----------------------LSPKNAKGLSTALKALGAN------SHPTGAL----LIEADTLQ---GRGVGSVDLLQE-----AKYRCDPSLVA-----------ASVIDLD-PELLRLAIDDILDEELK---------VDKVEIPTPQEFWQRRWLWCVNGSHSRVLDRRGGL---------------------DTRSIFPGVDRVYRRMYAEAQSEEPLTSWDAQV----------SVSASEKL-EHG--KTRAIFACDTLSYFAFEHLLSPVEKAWRG--VRVVLDPGTMGHLG-----------VANRI--ARI-----------QIGGVHVMLDYDDFNSQHST-SSMKV-LFQA--LTERIGY-----------------------------PPDLAG-------------------------NILSSFDNMWLYCK---GTLVGRAEGTLMSGHRATTFINSVLNAAYIRLSLGKEVY-----ERYKAVHV-GDDIYMNA--------PTHSDAALVLTRAQALGCRMNPAKQSVGTVGAEFLRMGIR----P-GGAVGY-FARSVASAVSGN-WVTEARQSPID---------------------------------------------ALKTAIANVRTLCNRSRS---------EAFPTL---LAGPLSR-LTRVPR------RLVHALLDGSKAIEGAPIFH---------SDGMIRTIT---EVPPPPEQ----------MAERL----------PPDWPR----------------HATNDYVVNCAT--AVEQFALK--------EAGRSVASAM-------------------LIASYNKALTSVAAPP--------------AYPRFKANRP----------------------------RAPVGSQPA--------------------LALLYTRKTSG---------------------VLQGYP------------------------------LLTLLKAHIS--------------RTLLRTLVILAGGDPN---------------------VSDLDAEAW------------GPD---------------NRSTIVQGSLS-----------------YADAAALSNKATTG------------------------------------------------------VLYT------------TYNVYM----------------------------

>UrV2_BLASTHit_AZT88643.1_RNA-dependent_RNA_polymerase_Thelebolus_microsporus_totivirus_1

M---------------------------------------------------------------------SFDARATERAATF------------------------GALGRYLVSELASS-----------------------------------------------------------------------------------------------------------SIPLDL---FFDKSISAQV-VLLAQYISRAPA----------------------------------------SGSIPVCAAS-----------------------------------------------------------------------------------------------------------------------------LLLSDFPVQAYLSTN-------------------------------------------------KIRQLALNAFDPLP------------------------------CPLDPSAALL-----------------------------------TGFSVMSNSALRE--------------------------AYFPRK------------------------------------------------------------------------------------------------------------SHPGASTKVNIFLSE-VLHELQFQG-------SLAIYDKTLRAIA--GRVTDDQA-------------------------------------------TACLLYIAGLRS-----QLGP---AAFEV--------GAAMV----------------------LQPANAKGLSTALKALGAN------SDRLGAA----LTEGNVLQ---GRAVGSIDLRAA-----AAERCDPEWVA-----------AHVVDPD-LPGLRTAVRSIISEELE---------GRFIEFDPLEHFWHRRWQWCVNGSHSRAINGRMGI---------------------DTVTSLPGIDRVYRRMVAETCTEEPISGWDGTT----------LVSASEKL-EHG--KTRAIFACDTRSYFAFEHLLGPVSAAWQH--KKVILDPGRFGHLG-----------MAQRV--LKA----------RRRGGLNLMIDYDDFNSHHAT-RTMQM-VFEE--LCSIVGY-----------------------------PTELSR-------------------------PIISSFDKCHLRAY---GKDVGHVVGTLMSGHRGTSFINSVLNAAYVRAAVGEPFY-----RTLEALHV-GDDVYMSP--------ATHSDAGLILASCAAAGCRMNPSKQSVGNVGAEFLRLAIR----P-THAVGY-LARSIASAVSGN-WVNESRLSTVE---------------------------------------------ATQSAVVTCRSLMNRSGF---------TGYPAL---LARSLNR-ASRIPL------RLCRRLLEGSVSQVGAPIYS---------VEGVMRNVM---ITPDPLER----------QADRL----------NPAWPS----------------SATGDYLSYAAA--APEILVLA--------QTDCPVKPAM-------------------LLSSYRKAVAGDLMVS-------------PSAPRVARLPD----------------------------KVPVGSASV--------------------FDLLQKPAKEG---------------------VLVRYP------------------------------LLPLVRRYIP--------------RPLLMELITLAGGDIN---------------------ARNLDIEAW------------GAT---------------ATSKVIQGVMS-----------------YSDAASLSARTLSG------------------------------------------------------TIYS------------TYPVYM----------------------------

>UrV2_BLASTHit_AZT88645.1_RNA-dependent_RNA_polymerase_Tolypocladium_ophioglossoides_totivirus_1

M-----C---------------------P-----------------------------------------NQDTELAERAKPF------------------------GVLGATLLPYIHRK-----------------------------------------------------------------------------------------------------------QFPQDA---F-NMPISDQM-IALLAPTFAGHT----------------------------------------WTSTTRVAAS-----------------------------------------------------------------------------------------------------------------------------LLLGKFPIQVPLTSD-------------------------------------------------EIIGLRAMAFKPRPL-----------------------------DLAAGEGRILAL---------------------------------VGGRVRGVTRQLM--------------------------EAFPLK------------------------------------------------------------------------------------------------------------RHAAASVKVNVYLSE-VLETILKLE-------QESYYDPLVDALA--GKVLADQA-------------------------------------------TMAIMYAMALRK-----HLGG---DARQA--------AYDAV----------------------MDPKSAKGLTTALKALGAN------SDMVGAM----FVESECLQ---GRGVGTTDLLAE-----ASYRCDPVEVS------------KVVIPE-KEGLENVVRTIIREELS---------GREIKFDSLENFWDQRWRWCVNGSHSRVLDSHMGV---------------------DSHGQLPGIDRMYRRMYAEMSRDYPGYGWDGTT----------YVSTSPKL-EAG--KTRAIFACDTRSYFAFEHLLSPVSAAWQD--KRVLLNPGRLGTLG-----------LALRI--NRA----------KSQGGIHAMLDYDDFNSHHST-ANMKM-LFRV--TMEEVGY-----------------------------PVELRQ-------------------------PLLDSFDKMHVSLR---GKEIGVMAGTLPSGHRGTTYINSVFNAAYIRHALGEDLY-----RDISSLHV-GDDVYSSC--------PTHSHASILLDRVGEYGCRMNAAKQSVGSVSAEFLRLAVR----P-TYATGY-FCRALASTVSGN-WTSSAKLGPKE---------------------------------------------FLASICASSRSLQNRGRT---------TALIPI---LARAASR-VTKLRY------RGLVPIIAGEAAIAGGPVFT---------ESSVIRTRE---YVEGPEERE---------LPSKV----------RRDWPT----------------HATDSYLATSLK--PVEVAALV--------LTKRSVKSVM-------------------LLSSYKKALVDDFSLRG---------------CQLRNVRD----------------------------RRPVGSASA--------------------QDLLFRPDRPG---------------------CLEKYP------------------------------ILPYLKGYLT--------------SMQLRELVRLANGDST---------------------ASDIRRVAW------------GEE---------------SRSKIIQGVLS-----------------YSDAASLSKRTSNS------------------------------------------------------LIYT------------TFDTYM----------------------------

>UrV2_BLASTHit_YP_009182167.1_RNA-dependent_RNA_polymerase_Ustilaginoidea_virens_RNA_virus_5

M---------------------------------------------------------------------AAHLSISERRAAL------------------------GHLGDVFYGMLEKA-----------------------------------------------------------------------------------------------------------EFPAEK---FRELRATEQL-LLLSVGGGVLNSSLALAVEFNRRVRSRVG--LKG--QPGLKTGVV-------VDPLLRVAAS-----------------------------------------------------------------------------------------------------------------------------MLCARFAVQVEMTDS-------------------------------------------------NVRRLVRLAFPE--------------------------------ALPPLWSGVPAE----------------------------VRGKVSLSALAARADLRE--------------------------VCFPYK------------------------------------------------------------------------------------------------------------TVPQATRKANVHLAS-LLTPKAREL--------VGGLDRLIGWLA--GRCSDDQV-------------------------------------------CSAIIYAHALGS-----RWGP---GAAEI--------AARYI----------------------LDPEGAVSVGLVLKAMGAN------SGPLGAA----LVEGKSLQ---GRGVGSLDLAKE-----AEQRCDPDWVA-----------GKVLHCD-PEELRLVIRQILSEELK---------GREIVFDTPEQFWERRWQWCVNGSHNRTWDARAGV---------------------DLPASMPGCDRFYRRAFSEVCKVETLTGWSGEV----------LAGVSPKL-ENG--KTRAIFACDTLSYYAFEHLLGPVSAAWLD--RRVVLDPGRVGHLG-----------MAERI--NRT----------RDGGGIDVMLDYDDFNSHHSN-TSMRI-LLEE--TCAAVGY-----------------------------DEELGR-------------------------KLCQSFDNTWVKTP---AG-LSRVRGTLMSGHRGTTYINSVLNAAYIRLAVGRAAY-----EGMVSMHV-GDDVYVNC--------PTPEGVEELVDRCAAIGCRMNPTKQSVGKVGAEFLRMGIR----R-EGAHGY-LARSVASLVSGN-WVNEKVMDPEE---------------------------------------------LLSSMVGTVRSLINRSGC---------ETVPQL---LAPAVSA-VTRIKV------ARCVSLLSGQSALEGRPVFNP--------RDGLIRTWA---LRVERPA------------SKVK----------AKDLPS----------------NATDDYLAKAAS--VLELRGAA--------MSTVDPRAAM-------------------LDSSYRKTLAGDTDPS-------------KIKLTLRSCTP----------------------------VLARGATNA--------------------RDLLRRPCPPG---------------------ALERYP------------------------------LLQLVKSGLR--------------DSDVRELVREAGGDWT---------------------ARDIGAEAW------------GIP---------------SRSRAISGVAS-----------------YTDAASLARRAETD------------------------------------------------------VVFF------------PYPVHM----------------------------

>refseqp|YP_009032633|843_AA|Beauveria_bassiana_victorivirus_NZL/1980_Victorivirus

M-----------------------------------------------------------------------IEALAKRALEY------------------------GPLGESLLGILSRF-----------------------------------------------------------------------------------------------------------PQVYSQ---FGVSTFTDDL-IRLRNVSGSIAS----------------------------------------LNQLLPAAIS-----------------------------------------------------------------------------------------------------------------------------LLCLPFPLQIQLRRS-------------------------------------------------DIIRLASLSLHLPFNPL--GRIDL--------------------VLADAHSYLPIK-----------------------NRSLSRSGPAILATFLNSEVVRD--------------------------QCFPVK------------------------------------------------------------------------------------------------------------KLAAAGSKPNVTACA-LARSFSSIF-------GSQVTGAVIALCA--GRLTDDQF-------------------------------------------CSVLIYAAGLHH-----HLGH---RGLAI--------AIWAF----------------------RSPKGAKGLSTALKALGAN------GTSLGAL----LCEMPSLQ---GRAVGDLDLYSE-----AAYRCDTTRVA-----------EKLLQPD-PHALRGHVRAIIEAELS---------GREVVLPDLNEWWSARWAWCVNGSQTARSARGLGL---------------------PTELLSETHDRMYRRMASEALTHEPVSSWDGVT----------DVSASAKL-ECG--KTRAIFACDTRSYFAFEWILGPVQRSWRN--SRVLLDPGFGGHVG-----------IVKKV--QAL----------QRGGGVNLMLDYDDFNSAHST-ATMQM-VFDV--LCERVGA-----------------------------PKWYSR-------------------------VLRDSFDKEYITIG---SD-RMHVKGTLMSGHRGTTFINSVLNAAYLRHALGGHYF-----DSLPSIHT-GDDVYVRP--------PTLADCDHILRSARDYGCRMNPTKQSVGFETAEFLRIAIG----K-TGAYGY-FARSVGSFVCGN-WANLDPLAGEE---------------------------------------------ALLTAINGVRSLCNRSQF---------EDLPRL---IGPALRH-TFGLGI------RSLVNLLAGRASIDGSPVFD---------TTGPLRVYR---LIRPPPE------------ELKH----------DPRWPT----------------HATNAYLTDHTS--EVEALALS--------KCGGDISRVM-------------------AASSYSKGRVIPGLRS-------------KPHFELRAKTI----------------------------PQPLGYVNS--------------------SELVVSSERGG---------------------FLSQYP------------------------------LLSLVKNRLS--------------DDDIRELVVAAGGTV----------------------TGPVREFAF------------GPA---------------SITKNFIGRLS-----------------YADAGMLSKRTTSG------------------------------------------------------NIFT------------IFQVCM----------------------------

>refseqp|YP_009508249|839_AA|Aspergillus_foetidus_slow_virus_1_Victorivirus

M-------------------------------------------------------------------------DIQTRISEF------------------------GRIGTYLFGLINGT-----------------------------------------------------------------------------------------------------------DFMDQI---NSHGTYVAKL-IALRNSMSSLRE----------------------------------------RHALLPAAAS-----------------------------------------------------------------------------------------------------------------------------LLLLPFPLQVELSIG-------------------------------------------------DVLLLLRSAFNLSY-----GTTIQ--------------------AAISSNAIPEIL-----------------------SDNLAGRARRIVTLVTQRPDFRE--------------------------KYFPVK------------------------------------------------------------------------------------------------------------KHPGAQTKANVHLAG-LVRSAVVLH-------GRGIIGRVLLATA--GRLTDDQA-------------------------------------------SSLLIYGSGLLP-----TFGA---LGWAI--------ATHMV----------------------LYPDDAKALNGALKALGTN------STPYGCL----LVEANTLQ---GRGVGAIDMREK-----ARERCDPDFIQ-----------KSVVSAN-FDELREHVRAVITTELS---------GRDVKMPTLSDFWTSRWAWCVNGSETAKSDEVLGL---------------------DPKAYKRTHTRSYRRMAAESVRDEPISTWDGTT----------YASASPKL-EAG--KTRAIFACDTRSYFAFSWCLNAVQKAWRN--SRVLLDPGIGGHLG-----------MVNRI--RHA----------QRGGGVNLMLDFDDFNSQHHT-VAMQI-VFDE--LCKYIGA-----------------------------PDWYRH-------------------------VLVNSFTRTHVSID---GR-WELVQGTLMSGHRGTTFINSVLNAAYIRMAVGGPYF-----DSLLSLHT-GDDVYIRA--------NTLGDCDYILNRCRDYGCRLNPAKQSVGYYGAEFLRVAIR----G-ERAYGY-FARGVSGFVNGN-WTSSDPLSLTE---------------------------------------------GLSSAIASCRTLINRSGD---------ASLADL---LGPAIRY-RRGLTT------RQTIQLLRGTLSLEGSPVFN---------TNYIIRNLR---VEGVDKE------------TVPI----------DNRWKR----------------YATTDYLTDHLS--PVEVEAIH--------LAKTDPGPLM-------------------IASGYRKGLNLDDKGT-------------PSQVRFRPLPP----------------------------RLATGFASA--------------------SQLTARRAEPG---------------------CLAKYP------------------------------VLSLIAGRLT--------------DDELRHLVAMEGGNNT---------------------TSDIRKEAF------------GES---------------PTSKNIIGFLS-----------------YADAAALSKVTSSG------------------------------------------------------NIFT------------SISVRV----------------------------

>refseqp|YP_009508251|834_AA|Beauveria_bassiana_victorivirus_1_Victorivirus

M-------------------------------------------------------------------------ATVTSPDTF------------------------GKIGIYLSDLLQRYGT------------------------------------------------------------------NNLPR---------------------------------------------GGTFVSRL-ITLQNSFSALRH----------------------------------------AHPLLPAAAN-----------------------------------------------------------------------------------------------------------------------------LLLLDFPLQTDIGLH-------------------------------------------------DFIALVREAHSLPPMF---DSLCI--------------------SLFPPQPGELVD----------------------------VTHGRLVRRLVRSSELRD--------------------------RLFPPK------------------------------------------------------------------------------------------------------------RLIAGETKTNVTLGG-CLRSAQRLL-------GSHKTALIARACI---GLPADHL-------------------------------------------CGVLIFLFCAWR-----KLGE---GALGV--------ALFLS----------------------SHPTEGKYASMVLKALGLN------STDWGAL----FCETQCLA---GRATGTVDVKAE-----ARRRCDPSKLT-----------GELIDVD-PDTLRDHVRSILRVELP----------NGCTVPTLDDFWTSRWLWCVNGSHTGASSDLLGI---------------------PRDFLSATHERVYRRAASETVKLEPLTSWDGYT----------SVSASQKL-EHG--KTRAIFACDTRSYFAFSWVLGSVQKAWRN--SRVILDPGTGGHLG-----------MAQRI--INA----------QRGGGVNLMLDYDDFNSHHSN-GVMAM-VFDE--LCKHVGM-----------------------------PDWYRD-------------------------VLVKSFDRIYYTDN---NG-RHKIAGTLMSGHRATTFINSVLNAAYIRAAIGSGRF-----DSLLSLHT-GDDVYIRC--------NTLADCAQILEATTAYGCRMNPAKQSIGFRSAEFLRMGIR----G-DKAYGY-LSRSISSLVSGN-WSSNDPLAPLE---------------------------------------------SLQTLITGCRAVINRSGV---------IDVAAF---LAPALRYPPQQISN------RTLIELLRGEVALEGSPVFN---------TQGRIQNYA---AYVPRAD------------ELPI----------PSSWKR----------------HATTDYLSYHVS--PIEAAALE--------WSGADAPSLL-------------------IASSYSKGLNKVGAAP-------------LPPVSFKRLPV----------------------------KHARGYVCA--------------------TDLSKRDVNPG---------------------VLTKYP------------------------------VINLVKSRLT--------------TEAILDLLVVELGYRP---------------------SGDPREIAF------------GGE---------------AESKCIFGTLS-----------------YPDAAAFSKLTTAG------------------------------------------------------NIYT------------LFSIAM----------------------------

>refseqp|YP_001109580|838_AA|Botryotinia_fuckeliana_totivirus_1_Totivirus

M-------------------------------------------------------------------------SAADRASAY------------------------GRLGSYLKELLDDH-----------------------------------------------------------------------------------------------------------PFVQGL---FSTTNFTQSL-IRLQGSSFTMRS----------------------------------------AHPLLPHAAS-----------------------------------------------------------------------------------------------------------------------------LLLLDFPMQTDTSVS-------------------------------------------------DVISLARNAYDLSDLG---EDVLINH------------------TLLPPGSPILSW-------------------------RRRGYHRKLGNAIITNKELRD--------------------------SLFPKK------------------------------------------------------------------------------------------------------------KHNAAGVKVNLTIGR-LAVAWARVF-------GPGSLGRHLAAIA--GRVTNDQA-------------------------------------------CSALLYCLTLRE-----HIGS---FGISI--------AQAAV----------------------TQPANAKGLSNALKALGAN------SSLPGSL----LVEAATLQ---GRFTNDVDMTNE-----IRSRTIQSLVD-----------EQVIDR--PEELRPHIKALLEMSLP----------GDCALPDMDEWWSSRWLWCVNGSETKKSDEALGL--------------------------RGGSGRRYRRMAAEEVHSNPVPDWDGTT----------SVSASIKL-EAG--KDRAIFACDTRSYFAFSWILNDVQKKWKG--ERIPLDPGKGGLYG-----------ISRRI--RNS----------QRGGGVNLMLDYDNFNSHHSN-SVQSM-IFEV--LCDKYNA-----------------------------PQWYKE-------------------------VLMESFNRMYIFKG---GV-RHRMLGTLASGHRGTSFINSLLNAAYIRCALGAAKF-----DTMLSLHA-GDDVYIRA--------NTLSEAADILKKCKMFGCRMNPTKQSIGFRNAEFLRLGIN----S-RYAVGY-VARTISTLVAGN-WSNLDPMEPLE---------------------------------------------SLTSVISSVRSVINRGAP---------TIIADV---IAYAHST-LHPYPL------KILKPLLRGEAAIAGAPIFN---------LHGKMRTYE---AVVSKPDN----------SGNPP----------PPEWGS----------------HATLSYLEYHVA--PIEASAIS--------RARVDLTSIM-------------------QESSYAKQVRSDLDDAS-----------RRTTVKLIPRTP----------------------------EEARGYIDA--------------------STLSTRDAKRG---------------------ILESYP------------------------------LIRLLENRLS--------------DEDLRELALLLGVSCS---------------------TKEARVCCF------------GSE---------------SLTHNIIGYLP-----------------YSDAASMSKTTNSR------------------------------------------------------NIYT------------TYHVRA----------------------------

>refseqp|NP_619670|835_AA|Helminthosporium_victoriae_virus_190S_Victorivirus

M------------------------------------------------------------------------SDPQERSKAY------------------------GLLGERLYAVASAN-----------------------------------------------------------------------------------------------------------SHMLAG---YDSLDFTARL-VRLTGEATALKA----------------------------------------VDPLLPCAVS-----------------------------------------------------------------------------------------------------------------------------LLFMDFPLQLPCTPE-------------------------------------------------ETLRLVRRAYDPNTL----EEVDY--------------------STLSGYATQFAR-----------------------VKGQRGRWRHLGHLVCNDKAFRE--------------------------RYFPKK------------------------------------------------------------------------------------------------------------KHAAAAIKTNIRLGP-LARAWAARY-------GLAALGSHLAYMV---GMPNDRA-------------------------------------------CATLLLAQTYKA-----RFGS---EGVAW--------AIASV----------------------RQPENAKGLSNALKALGSN------TSEPGAL----FVEANTLQ---GRYDRTLDMDHE-----VESRCSPAAIA-----------DQVIPY--TDELGACIDFILDTELG---------GDTIELPDEDEWWTSRWLWCVNGSQNALSDKALGI--------------------------KNKSGQRYRRMAAEEVNNNPVPAWNGHT----------SVSPSVKL-ENG--KDRAIFACDTRSYFAFTYWLTPIEKKWRG--ARVILNPGEGGLYG-----------TARRI--RGS----------QTSGGVNLMLDYDNFNSQHSN-ETMAA-LYEK--ALSRTNA-----------------------------PAYLKK-------------------------AVAASVESTYIHYK---GR-DRHVLGTLMSGHRATTFTNSVLNAAYICYAVGIPAF-----KRMISLHA-GDDVYLRL--------PTLADCATTLNNTKRVGCRMNPTKQSIGYTGAEFLRLGIN----K-SYAIGY-LCRAIASLVSGS-WTSLDELQPLN---------------------------------------------ALNGAIVQTRSCLNRGAA---------TGLPEL---ISASFVG-LRGFKR------RDLLELLTGVATIKPGPVYT---------SSCVIREYV---VEQPPPP------------QFDV----------PPGAGM----------------HATMSYLARHTT--LVEAQALE--------IARPAIKSLM-------------------LSSSYGKAGPGAQTRPH------------VPMPKLRRQPP----------------------------RVAVGFSMA--------------------HELTGRGVKEG---------------------CLSGHP------------------------------LLRLFEQRLT--------------DDDLRALVALVGGNTS---------------------AKDIRAEAF------------GAE---------------SSSSTIMGILP-----------------HSDASNYCKRTRNG------------------------------------------------------NIIV------------PYHIRS----------------------------

>refseqp|YP_009072433|840_AA|Botryosphaeria_dothidea_victorivirus_1_Victorivirus

M-----------------------------------------------------------------------QDAVAVRAAEV------------------------GPLGVELLRVIDTF-----------------------------------------------------------------------------------------------------------AAWTRT---HFPRELVAGM-SRLQVQRQQLDS----------------------------------------IHPLLAPAIA-----------------------------------------------------------------------------------------------------------------------------TLLLDYPLQLELDHG-------------------------------------------------VVMRLVDLALPRID------------------------------AEPAEPAKNQALR-------QH----------VKRNSPRHNQYMELASAIRHDKPFRL--------------------------ACFPEK------------------------------------------------------------------------------------------------------------KLQAATAKKNFSTRR-LLDSVSREL-------GASFLGWLVAHCA--CKVTDDQF-------------------------------------------QMIIIFGLTLST-----RLGR---HAYLY--------ALSMV----------------------TNPSHAKSLNVVLKGLGAN------ASTPGCW----FVEGQGLL---GRGVGDVDWNSE-----IPYRCDPDLVR-----------EKTINVD-PETIRPHIRAILERELV----------NRNDLPDMEDFWTSRWLWCVNGSQTSDSDKALGL---------------------DLK-TLQTHRRRYRRMAAEALVDNPIPDWDGTT----------YVSASSKL-EVG--KTRAIFACDTRSYFAFSYLLNQVQKDWRN--ERVLLDPGNGGTVA-----------MGARL--RNA----------QKGGGFNLMLDYDDFNSQHST-ETMKI-VFEE--TCKIYNA-----------------------------PAWYTD-------------------------KLCSSFDKMYIRQK---DG-LRHVAGTLMSGHRGTTFINSVLNAAYLRCGVGSGWF-----DHALSLHT-GDDVYIRS--------NSRSEVSNILTRAAEFGCRMNPTKQSIGVKNCEFLRCAYN----P-YYAVGY-LCRTVGTLVNGN-WSGDTPLTPHE---------------------------------------------ALTSLLTSLRSLYNRSLG---------GGLGRY---LASAIRFRTDGISR------KNLIGLLDGRIAIEGGPCFN---------ADAKIRTCA---LKNTIKP------------SPLV----------DKNWDA----------------YATRDYLVCHTT--DLEYYALQ--------NCGVSPTPLM-------------------VTTSYEKGLNRESEEV--------------KPLKFCNMRV----------------------------STARGFATV--------------------SELLKVDTEGG---------------------VLAHYP------------------------------LLMLIKDSLS--------------PSLIRELCEADGYTPP---------------------SENIRAYAF------------GED---------------FRTHNIIGRLP-----------------YSDAAALQGKTSCD------------------------------------------------------NIVA------------DYKIYL----------------------------

>refseqp|NP_898833|845_AA|Helicobasidium_mompa_totivirus_1-17_Victorivirus

M---------------------------------------------------------------------KALRAAQDKAGEL------------------------GPLGSALLERVLAK-----------------------------------------------------------------------------------------------------------PEVFER---VSRGNIEEQN-MEVANLQENGMG--------------------------------------------VDAAAF-----------------------------------------------------------------------------------------------------------------------------LSSLSFPIQVKLNQA-------------------------------------------------DIITLARLATDTRAYCPLINRTLD--------------------TAVARKAGVRSAW-------T------------------EREGHRLLNNVIGDVNVRK--------------------------STVPSE------------------------------------------------------------------------------------------------------------ATPAATIKANLSLGR-TLLCAPKVL-------GHSIIARIVWNMA--GKCSSDVI-------------------------------------------TAAILYTAALLP-----SHRN---RSWRI--------AVAAC----------------------LDPKAAKGLSTAMKSLGAN------SVQEGAV----LVEAQSLQ---GRMTGACDLAEE-----ASYRCDARKVA-----------EQVISAD-PEALRDSIRWVLNKELA---------NGVDEYQSLDDFWSRRWLWCVNGSHTNASDAKVGI---------------------RRKVDLPGIDRVYRRMVAEALEEEPISCWDGTT----------FVSATQKL-EHG--KTRAIFACDTQNYFRVSHLLGPVQNRWRN--ERILLDPGEGGSLK-----------MAHRM--MDM----------RAAGGLNVMLDYDDFNSHHAT-ETQKI-LFEE--LIKHVNY-----------------------------DPVLGK-------------------------TLVDSFDKMYCYIK---GVRVGQVLGTMMSGHRGTMFINSVLNAVYIRLATGAAFF-----DPLSSLHA-GDDVYAVL--------PSLSDAKFLLDSCHNFGCRMNSTKQSVGVVCGEFLRMAVT----P-VGAIGY-VCRSIASFISGN-WSTDAPLTPRE---------------------------------------------YLTNCISQCHTLFNRSNS---------MHLIQL---ISDCIHK-RLNLKR------KLCLEMMTGLAAPDGVPLFNT--------MYGRVYPIV------------------------------------PENWRRRGARF----DCACEVHHATSQYSSQHLP--PVKVEALT--------ETGTSAASAM-------------------QLASYKRAIDSMLSYE-------------PRKVKFGKPRT----------------------------MNALGLNLA--------------------KELFTHLLNTG---------------------CLEAMP------------------------------VLRLIQDQIK--------------RPLLRELVLLAGGDGG---------------------AADLEVEAW------------GPQ---------------TRKCSFFGWIP-----------------YSDAAALQKKADHI------------------------------------------------------KIMN------------HTPVRM----------------------------

>refseqp|YP_044807|825_AA|Gremmeniella_abietina_RNA_virus_L2_Totivirus

M----------------------------------------------------------------------IESPVSIRANEA------------------------GIVGQYLKGLLN-------------------------------------------------------------------------------------------------------------MEWASG---VMVLSFSQQI-SEVYKPTFNGLR----------------------------------------PTDLQRAAAA-----------------------------------------------------------------------------------------------------------------------------YLVPDFPVQVRIERG-------------------------------------------------SVLSLLSQVIDPPA------------------------------RVTDRHS----------------------------------------FRWLCDPKASY--------------------------AAFPPK------------------------------------------------------------------------------------------------------------QHPGAVNKVNVYLNE-VASSLLSLDP-----AAYSSASQALWPYR--GKIANDQA-------------------------------------------SAIILYGYGLRA-----QAVP---DAMHV--------AATLA----------------------TTPDLAKALTNFLKATGAN------GSRLGAL----LCESNVLL---GRAAGPADLSEE-----ARYRTSSDVES------------RLAIFS-DADLSAAIDSVLDEEIKRVE-----GSQHIEFDSYQEHWNDRWAWAVNGAHSGHVSKLY-----------------------PRVPKPPGMLREHRRAWLESVTEDPRIDWDGKT----------FVSASPKL-EAG--KTRAIFACDTVNYLAFEHLLAPVEKRWRN--SKVILDPGRGGHLG-----------MIFRT--TAA----------RARAGVSMMLDYDDFNSHHST-RAMQI-LFQR--LGDRVGY-----------------------------PADKLA-------------------------KLVASFEKMYIYNG---MEQVGRVRGTLMSGHRGTTFINSVLNKAYLLIVLGEDLF-----ERSXALHV-GDDVYFGV--------RTYAEAGEVVTRIKNSPLRMNRMKQSVGHVSTEFLRNATS----G-RSTYGY-FARAVASTVSGN-WVNEMALSPSE---------------------------------------------ALSSIIGAARTLVNRSGA---------ENLPLL---LHPSLVR-MTGLPRED---HKKLRELLLGTTALDNGPQYS---------LGGYYTSVS---SLITIAA-----------SDRHG----------YTPLPR----------------EATTAYLSCAAD--PLEVNVLT--------QAGVSVVSAM-------------------EEASFRKSLPARYTGY--------------ETLRLGPKSL----------------------------IRSIGTASV--------------------ADLISVSPPRG---------------------VLERYP------------------------------LLTLAKRRLP--------------EYLVRWAVSVAGGNPS---------------------APDIGMEAW------------GEF---------------KHGCMIATPMS-----------------YSDAATFGKRTVST------------------------------------------------------VLTC------------PLDAHV----------------------------

>refseqp|YP_392467|829_AA|Coniothyrium_minitans_RNA_virus_Victorivirus

M----------------------------------------------------------------------IGARVDERALAA------------------------GALGQYLKKLTR-------------------------------------------------------------------------------------------------------------SQDVSR---FATLSFPDQI-SYVFKPSWDSFR----------------------------------------PTPLHRAALS-----------------------------------------------------------------------------------------------------------------------------FLCGEVPVQVALPYA-------------------------------------------------SLWRMVDYTCPVPD------------------------------IVLDIK---------------------------------------YKTRWIRDAGITV--------------------------KRFPLK------------------------------------------------------------------------------------------------------------KNPAASNKVNLYLHE-VGRDLSSHAP-----TQLNVGLQYLDWVRRQRVVYNDQA-------------------------------------------TAFLLYEYCLAC------YDS---RGWRH--------AAASL----------------------FDSDYAKGFSVFGKAVGIN------GSQVGAM----MVETNVLL---GRDVAPIDLVEE-----ARKRTSMREVR-----------AMTTAYS-DDTIRRAVRTVLLRELKRSG-----DSYILEFPTLEDHWATRWQWAVNGAHSGLIYKTN----------------------PSYRPHMPGFDRLHRRAWLETISEDPRPAWDGRT----------YVSASPKL-EHG--KTRAIFAWDTINSLAMEHLMSTVEANWRG--ERVILNPGKGGHLG-----------MAQRV--QAA----------RNRSGVSLMLDYDDFNSHHTT-RTMQI-VVEE--TCRLTGY-----------------------------PPDLAE-------------------------KLVSSMEKHYICVG---GAYIGRSKGTLMSGHRLTTYINSVCNEAYLRIELGDDFL-----DKNVSLHV-GDDVYLGV--------RSYQEAGYVLRMIRGSKLRMNPAKQSVGHVTTEFLRVASE----S-RYSYGY-LARAVASITSGS-WVNELALAPLE---------------------------------------------ALTNIVASARSLANRSGI---------ADVALL---LVSSTRR-MAPLDSRD---DTLLRELLTGKVALQNGPNYQ---------SSGYYRHVA---VTPKMVR-----------RDDFG----------YGVLPL----------------EATHTYLSSAAT--VLEIETLT--------KAGISVEEDM-------------------ARASYKKSAPRDFFSA--------------ECLVTGPVLQ----------------------------RPCVGVEWA--------------------EMVLRRPRVTG---------------------ILSRYP------------------------------LLLLARYRLP--------------ERVVREALAAAGGDYN---------------------TPYLDYDAW------------GEY---------------AHGCVIDTVMS-----------------YTDASALGAKTAAG------------------------------------------------------VLTS------------TTRMYV----------------------------

>refseqp|YP_009508253|827_AA|Epichloe_festucae_virus_1_Victorivirus

M----------------------------------------------------------------------IESTVADRANAA------------------------GSVGRYLEALLP-------------------------------------------------------------------------------------------------------------SEYTRV---VSTLPFDHQV-SFVYRPSWRHHK----------------------------------------PTELQRVAAA-----------------------------------------------------------------------------------------------------------------------------FLLPRVPVQVSYSAG-------------------------------------------------SILTLLNATLP---------------------------------NLPPRHT--------------------------------------YHAGWSCDSDATR--------------------------KAFPLK------------------------------------------------------------------------------------------------------------ANPAASNKVNVYLSE-VCYDLRRTNP-----GEAMAAEAALQRVRSSGSVYNDQA-------------------------------------------TAVVLYGFCLAR-----NGLP---GAYDI--------ALALL----------------------LNPDYAKSLTGVLKATGGN------ATAVGSL----LVEANTLL---GRDVAPVDLGKE-----FNMRTQLRLAR-----------ENMAEYP-PDVLRATVRRVLLDELTRRA-----GSYELDYPSLEEHWDSRWAWAVNGAQGGAITDK-GL---------------------ESAPRPPGATREYRRSWLERVEEDPRPQWDGTT----------EVSASSKL-EHG--KTRAIFACDTVNYLAFEHLMSTVEANWRG--VRVVLNPGKGGNMG-----------MAMRV--QAA----------RRRCGVSLMLDYDDFNSHHTI-SAMQI-VVEE--VCSITGY-----------------------------DPDLAA-------------------------RLVASLDKQYITLA---GR-RKLSVGTLMSGHRCTTFFNSVLNAVYVRLELGEPFY-----SETVSLHV-GDDVYMGV--------RDYVSAAYVIQRLADSPLRMNALKQSVGHVSTEFLRIATG----A-RDCYGY-LSRAVASIVSGN-WVNDTALAPFE---------------------------------------------GLAAMVASARSLANRGKT---------NLAPLL---LVSSVLR-IAKLGKRH---YGKVSSLLDGTVALGNGPQYS---------CGAHMRWCN---PVVVRDT-----------PDPWG----------YTELPL----------------ESVTAYLSRAAS--PLEWEVLG--------EAGISVTEQM-------------------AAASYRKTYASLFWRG--------------ERLTLGPTLS----------------------------KAPIGSASA--------------------EDLARATKPHG---------------------LLAQYP------------------------------LLLLARDRIP--------------ETLVRVALGKAGGNPH---------------------TSHLAYDAW------------GEY---------------DHGCQIQTVLG-----------------YADAAAFGKRTSAD------------------------------------------------------VLTS------------RTLMYV----------------------------

>refseqp|YP_009272905|827_AA|Fusarium_poae_victorivirus_1_Victorivirus

M-------------------------------------------------------------------------SPIDRGDGE------------------------AFLDGVLDSILKRF-----------------------------------------------------------------------------------------------------------PGQLPD---VAGLPFDEQI-SAAYSPSWGGVR----------------------------------------PSGLLRAAFS-----------------------------------------------------------------------------------------------------------------------------YRHTTVPVQSIYDHS-------------------------------------------------DLRRLLNSVVEFFP------------------------------LSTDFSS----------------------------------------FKILKHPKNSL--------------------------KLFKPK------------------------------------------------------------------------------------------------------------RLPQALTKANLYLDE-VLRDLCKNRP-----TLGEEASALLWSLR-DRGITHDAA-------------------------------------------TAIVLYGSALS------QFYT---DAFHW--------AATAV----------------------LYPKLAKAVSNFLKATGGN------ATSFGSL----LVECEVLQ---GRGVGTIDLLAE-----AKLRCSPDYVRD----------HYAAAFD-EEKLRRAVRRVYETEISHED-----DSQRVEFPTLEEHWDSRWVWAVNGSQSSLLDGGR----------------------VKKLLEPLGLHKLHRRAWLECTPDDPRVGWDGTT----------YVSPSPKL-ENG--KTRAIFACDTRHYLAFEHLLTPVEKRWRG--SRVVLNPGKGGNIA-----------MAQRV--RHS----------RDRSGISLMLDYDDFNSQHTT-SAMKI-VIEE--LCSVVGY-----------------------------PADLTA-------------------------TLVSSFDKMRIHVA---GKYVGVAAGTLMSGHRATTFINSVLNKAYLDVVLGEGWL-----DTRRSVHV-GDDIYCGV--------KSYRDAAYVVHQITSSPLRMNPTKQSVGHVSTEFLRLATA----G-RDTYGY-VARSIASLISGN-WVSDRIMNSYE---------------------------------------------ALTTMVASARTLANRARD---------VTLPLL---LESAVKR-VLSKDCID---DRVIRRLLLGEVAINNGPTFS---------SSGSYTRVT---VRAEYTA-----------RDTVG----------RPKLPH----------------QSTSRYLTTNTT--DFECETLS--------QAGISPEQTM-------------------IESSYSKSLQFGDLYF--------------DRLVAGDVES----------------------------TPARGSVGV--------------------EWLVHTTPPKG---------------------VLSQYP------------------------------LLVLVKSRLP--------------EYVVRDAVRRAGGNPQ---------------------CTDLELEAW------------GEY---------------SHGCIVNDVLA-----------------YSDAAAFGKRTDCS------------------------------------------------------VLTS------------THRYYV----------------------------

>refseqp|YP_009115500|1071_AA|Eimeria_tenella_RNA_virus_1_Victorivirus

M-------------------------MVPKE--------------------------------ATASHPPGALWGAVSKSGVL------------------------SVLGRSFHAAGNMASK----------------------------FDADVIRSELVSAAQGQPSEDSGYATLVAYNLAYNPYPIHLPLPLSWRSEVIRWLTYYSLITMSSWWVATDVVGFWSSGGFCE---IAPADFVERA-APRVNPLEAGVR----------------------------------------LESLVKAYRD--------------------------------------------------------------------------------------RASYASRVGPELHRRYIAPDDGGNPFR------------ALQAGSPSRARLQSPRVRPARRKRAQWRCTAFADVIAEITGLVCGQLKTLYSGVREYARSEVYEAVMRCVNRALCHAHLSG--GEGSAGGAGPGAMRHQSESGRSVPWRPQAHEAGSSAPW-------THRVPESYLAEVAERISPRDV----LSELIARDSSIALRQEL---------------------GRLFPRK------------------------------------------------------------------------------------------------------------RDPQSGRKANIYLDC-LLADIRDIDP-----ASHRQLCMMLSAAP-PGAIHEDQV-------------------------------------------CNALIYAYSLWRGR---EALL---GAFCI--------LVNLA----------------------EGGERLKHLSDILKASGSN------TVFEWAQ----LCELNCLL---GRGVGDTDLWSD-----AERRTRPEKDM-------------EVELD-RRRLSDAINAVLAEELG---------DEPLHADPIQDYWDRRFEWCVAGSHNHTTNAEGAFA--------------------DVPATGPGGVRMTRRMAMEHVDENALVTWDGHV----------QVSVVPKL-EQG--KTRAIYSCNTVSYAAFGRILRPAENRWAG--RRVILDPGAGGNYG-----------MFRRI--RKAW---------PRALGTALMLDYSDFNSQHTL-AAQEL-VVKL--LCDRFTN----------------------------LEPDYSE-------------------------LLVSSLHKMDIYVH---GKLVGRARRSLMSDHRGTSFINSVLNAAYIRYVLGEERY-----RKMQSFHV-GDDVLMFC--------KDSVEAYGVIGAMEDAGFHLQRSKQSVGSKGFEFLRMAGT----R-SLAHGY-VARSIASLVSGN-WTTDWTYDPVA---------------------------------------------TMHSFVQQARSLMNRSGN---------VSAYLL---LGRSMQV-YTSLPL------GVIHEVLSGRVALGAGPVYR---------ADGRYETRA---ILEVGDYNVR--------EDLPT----------WRRLPR----------------AATADYFTRGCH--AVERLAME--------LVGFQPWAAA-------------------LRASYGALAVRRLQEGL---ACRDPAADIPTSVTLGCKQL----------------------------YCKAGAVDL--------------------DSELDRPTRRG---------------------ALVQYP------------------------------VIALLQNTLT--------------DGQIGELLRYLGVPFQ---------------------AGQERITAF------------GNP---------------SVGVVIQGDLP-----------------YSDASALCSKGILQ------------------------------------------------------TVRV------------IYPLRM----------------------------

>refseqp|NP_108651|884_AA|Eimeria_brunetti_RNA_virus_1_Unclassified_Totivirus

M-----------------------------------------------------------------------MTELLPRLTPVVA--------------------ADEMGELYLRTTFPRPPLAG-------------TFNDTGLFP-------------------------VMYAETASLQHAVRRILRHGPL------------------------------SLTQSECLAR---STFTAVGDEA-VLCRRAEEAFWD-------------------L-----------------------------------------------------------------------------------------------------------------------------------------------------------LLASEWPMHVAPVPW----------------------------------------------LLKHVVTLIARGLKQSLLE-------------------------------------------------HRIPGGWI--------------KHIRNGRSPSKEWLL--------------------------GYWPRK------------------------------------------------------------------------------------------------------------SHPLAGKKANLFLDC-VIADFLQNYK-----CLGPVLEGWMKRTH-LLGIYEDQA-------------------------------------------CNIIVYAQAVML-----EFGF--EESLYL--------ATWAV----------------------LRAKQAKALSDVLKAMGQN------MCAVGRH----LCELAVLQ---GRGVNRLDLLED-----AERRVSATFNC------------PIVHVP-ADALRQCVRDVLRRELR----------TRVVLPPSDDFFRRRYAWCVGGAHNLNGNDHWLPR--------------------SLLPQFPAALLWNRRAALNCVERNPLKRWSGHV----------RVSVAEKV-EQG--KGRAIYSCDTLSYTAFSWLLEAVEKEWAS--RSVILNPGKGGTLG-----------MMNRI--RGA--------ATGGRRQCYLMLDYSDFNSQHSN-EAMRI-VIEE--TLACCDHN----------------------------EPELAA-------------------------KLLKSIDDMHIYLR---GVHIGRVAGSLMSGHRATTYWNSVLNAAYVRYAIGRVAY-----DTYKPFHV-GDDVLILL--------DSPADAWNVVKKLEHIGCTLQRSKQSVGIGGYEFLRVAGS----PFTGVGGY-VARSVAGLVSGS-WVSSVKMAPRE---------------------------------------------ALQSLIQQARSIMNRSGN---------PAAYKL---LISSARR-TIGIDQ------QYLEEFLSGNVALAPGPCYR---------SDRRYVARH---VEWESSA-----------DDPYGGGRLLIGGIEIEKLPN----------------HAARDYVNWAAS--EVERVGLA--------MVGRVPWRAM-------------------ALTAYAAMEPSLYGNPSGG----------VRAAYISPRTV----------------------------RLIDHYIDA--------------------ADITTTDSQHG---------------------ILAQYP------------------------------LLSLLRNYFS--------------MEDLTYLLNFVGRDA----------------------GIDARVTAW------------GGT---------------NEGICVCGLLP-----------------YADAAGAASRVLGN------------------------------------------------------AIHV------------SVPVAV----------------------------

>refseqp|NP_041191|874_AA|Leishmania_RNA_virus_1_-_1_Leishmaniavirus

M-------------------------QCP-----------------------------------------NQNHMLVNRAMVV------------------------AALDSFEDARRIISG----------------------------------------------------------------------------------------------------------VLDLSR---LTNTSVKGQH-TNDTNYFSRYSNFFSQ--------------------MPAIILNQLKCCIKAQVDDAVKMVLQ-----------------------------------------------------------------------------------------------------------------KA---------KKVEVSKPVEKMLTFTTLNT---------YLGYPES----TGCV---MEYTEEQSGPI----AAKLLITLLSSTLNAMVRP-------------------------------------------------------------------------KSDPNISQNKIPR--------------------------HYYQLK------------------------------------------------------------------------------------------------------------VHVGAQKKVNLTAIE-VIRGCQHEC--------SVVYCEFVRYSAYFAGLYDDQV-------------------------------------------AAILLYAVAAHN-----VQGF--GARFCVLW------ALMCV----------------------RIAGFADDINIYIKHRGMS--------GLLPQ----LVEMKCLL---GRGVNEIDVETE-----ARRRLDVGSLS-------------MQRLD-ENELRAAVRLIYSEELR----------RPVTYPLICDFWSSRWLWAANGSHSRALEHA------------------------HPELATRKEGQAYRKAVMEQWQHNPMDRWDGTV----------YVTPSAKL-EHG--KTRLLLACDTLSYMWFEYALRPVERIWEN--SNVILDPGSMGNCG-----------IATRI--NGW--------RNGMPGQSFFAVDYDDFNSQHTL-MSQKI-VFEE--LFHHIGY-----------------------------NASWVK-------------------------TLVDSFDSMELWIK---GKCAGIMAGTLMSGHRATSFINSVLNRAYIICAGG---------HVPTSMHV-GDDILMSC---------TLGHADNLIANLNRKGVRLNASKQVFSKTSGEFLRVAHR----E-HTSHGY-LARVISSAVSGN-WVSDHTLNQQE---------------------------------------------ALMNAIVCCRGILNRSLPG------EKNPVVRV---ISRSVSK-RTKIEE------KTIRLLLSGRACLKGGVVYG---------EQTNYIQVYRINCRVERS---------------------------EEKLPP--------------YRHATEDYLNNHLA--DIEVMAVR--------QYGSDIADIM-------------------AQASWKKSMSTEGAEDV-------------SRLSLQRDKT----------------------------LPCLHCITE--------------------KETSLLPVRYG---------------------LFSSYP------------------------------ILMMLKDRIP--------------IKEALKLAVTIGYRPQ-------------P-------NSDLELDLW------------GES---------------NNSCAIEGVLP-----------------YNEATSLAQKLPCGG----------------------------------------------------VVIQV------------IHNVYV----------------------------

>refseqp|NP_043465|777_AA|Leishmania_RNA_virus_2_-_1_Leishmaniavirus

M-----------------------------------------------------------------------------RAKLT------------------------KVVEGDLS--------------------------------------------------------------------------VFMPN------------------------------------------------------ARIKRESQNASD----------------------------------------LHHFLESVVE-----------------------------------------------------------------------------------------------------------------------------GIAIPQPDSLILSLE-------------------------------------------------GVL----------------------------------------------------------------------------------------------------------------------------GHKYKLK------------------------------------------------------------------------------------------------------------QHIGASTKVNIHASE-VIAGSRKYA--------RTVYRQYVQMSSLFSGMYDDHV-------------------------------------------AGILLYSISTLK-----HCDV--HVAMAY--------AIAAVRD--------------------LTQSVIADLSDVLKLRALA--------AWCPN----YVELKCLR---GRGIAELDVIAE-----AKQRLKKPTHP------------YAYADN-EELLRNAIRRVFDEELP---------RDKVVLRPKDDFWSSRWLWAANGGHSRALEHA------------------------HPELRTRKEVRAYRKCVLEQWRNNPMDAWDGQV----------YVTPSQKL-EHG--KSRLLLACDTLSYLWFEYYLKPVETAWLN--KRTLLNPGVVNHYS-----------MAKKV--KVLLADAEHDETNIERGTKLYSLDFEDFNSQHSL-AAQKM-VFEE--LFKHVGI-----------------------------ENEETR-------------------------KVVSSFERMIVYNG---KEPLGRVHGTLMSGHRATTFINTILNTAYLYVAGL---------HTDFSYHV-GDDVIFST---------GCAEAARLYDCLKHYGVRCNPHKQCASEYSGEFLRVAHT----K-HYSTGY-VARAIASCVSGN-WVSDHVLDRRE---------------------------------------------ALTNAISCVRTIMNRTQSD------EDNPIGRV---VALSVAK-RCCVEE------KHIRKLLYGKACLGEGVIYG---------KLCSSATKVDINVNMDYDKTR------------------------TFKYEM----------------FATADYLRHHTS--YAESVMLR--------QYGADIADIM-------------------AQSSWMKSMEASSRVSP-----------SSMRVTLSRSDL--------------------------------GVFTM------------------PVCDVERFPVRKG---------------------VFEKYP------------------------------LLMMIKERIP--------------LREALDLAQGIGYIA----------------------PHGCAEDLW------------GGT---------------MRLCAIDGVLP-----------------YAMLVNWHAPVPCNG----------------------------------------------------VRLVV------------DHNVYA----------------------------

>refseqp|YP_009162330|1430_AA|Trichomonas_vaginalis_virus_1_Trichomonasvirus

L-------------------------------------------------------------------------SKAVRCGPII-----------------------PSVKHHFNIRRI--------------------------------ITVKRNGNEYVFI----------------------------PG---------------------------------------------------------------------------------YGWVL--------------------QDDYLVNSVK----MTGED------------------------------------------------------------------------------------------------------------------------QLPPNQLPYGD-------------------------------------------------DLLLIYSEILLYNYI-----------------------------SLFPKFR--------------------------------------YKNPDLLNQETEL--------------------------QLFPLK------------------------------------------------------------------------------------------------------------TDSAARNKANFYARS-LWNEAKTDK------------------TAFKPGTYNDTV-------------------------------------------AGLLMWQQCALMWSLPRSVIN--------------RTISGVCD----------------ALTERTSLALLKRISDWLKQLGLA------CSPIHRL----FIELPTLL---GRGAIPGDSVKD-----MKHRLKFDPSI-------------TVDVP-RDQLHDLIYRLLSRNLH-----------ITNVESFDHHLEERLLWSKSGSHYYPDEEVNRL----------------------------LPNQPTRKEFLDVVTVDYIKECKPQV----------FIRQSRKL-EHG--KERFIYNCDTVSYVYFDFILKLFEAGWQD--SEAILSPGDYTGER-----------LHARI----------------SSYKYKAMLDYTDFNSQHTI-RSMRL-IFET--MKELL-------------------------------PPET---------------------TFALD-WCIASFDNMYTSDG-------HKWVSTLPSGHRATTFINTVLNWCYTQMVG----------LKFNSFMCAGDDVILLS--------QEPISLVPILTSH----FKFNPSKQSTGTR-GEFLRKHYT----S-EGVFAY-PARAIASLVSGN-WLSQSLRENTP---------------------------------------------ILVPIQNGIDRLRSRAGL---------LGVPWI---LGLSELTEREAVPR------DVSMALLNSHAAGPGLITRN---------YSSFTV---------------------------------------TPKPPT---------------LTSTLEYTATRYGVQDLSKHVPWE---QLTLEERNKLGKQI-------------------KKMSHRHCSQAKITYTC-----------------------------------------------VHEVYKPSGLPKVL---------------------------------------------------SGASQP------------------------------SLSMVWWQAM---------------------LKEAMQDNS---------------------TKKIDAQMF------------ASS---------------ACTDRVSGDAF-----------------LQASAKAAGVLITS------------------------------------------------------LIQSSS--------------------------------------------

>refseqp|NP_624323|1436_AA|Trichomonas_vaginalis_virus_2_Trichomonasvirus

R-----------------------------------------------------------------------FQRTVTRKDP-CG--------------------------------------------------RQLTPQDMRCLP-GPQLRAKAGQRHVERINQCGGS-------------------VIYPR----------------------------------LSTARA---CSLSAIDKMTLALAHQLCYLYKS--------------------------------------SDLHRQLDTMIP---------Q-----------------------------------------------------------------------------------------------------------------------------------------------------------------------------------SYLTFLEWLL----------------------------------RIDPHNEKSSIRHFPSQD--NHEV--------------------ITHSLRNLTKEQEI--------------------------TLFPIK------------------------------------------------------------------------------------------------------------DIVQANRRVNAYARN-LLDASPLPD-------------FALQQML-LPNTANDVV-------------------------------------------CAILLLGEVLWMLRCPISIIV------NI--------SRAIC----------------------RNDSFLKDLSDFNKMLGLT------KIPIANC----LTELNTLQ---GRGVTSSDAKRD-----LTHRIA-DVNP------------HEAKIS-RENLREAINQIYKEEIT----------RKEVPDTFKQHVFTSPLWVKKGAHHHPL----------------------------------FGSYDNRLEFVENVDLDRVLKSHPAV----------YITQAPKL-EHG--KTRFIYNCDTVSYIYFDYILNYIESVWSN--KHVLLNPDY----------------MNPVIF-------------STLNYDEYCMLDYTDFNSQHSI-ESMKQ-VFLC--LFPFL-------------------------------PRSM---------------------HSILQ-WCVTSFDNMYINKT--------HWNSTLPSGHRATTFINSVLNRAYLLPFL----------QVANAFHT-GDDVLLCG-------------KADYATLINTVPYELNKTKQSFGPS-AEFLRLHKH----N-DQVSGY-PARAISSLVSGN-WLSFANPLWQP---------------------------------------------SLLSIMQQLYTISARSGLL--------PYIPVT---MKLEVQRRYDLR-------SRITNGLFSGDIVPSGCPCYK---------SNAALL---------------------------------------SAVVPDTVVK-----APPTFYDLRTLDILKQTSP--WINSASR---------------YMDL-------------------LERRHMESDNKNVIY------------------------------------------------------------------------------------------------------------------SIQYLPSK----------------MLPMIDVDPAD-ATPLRKRYHP---------------------------------------------------RSHIAHPLP------------RDA---------------HLKELRFATCRVG---------------PATAIRLGSLWPAN------------------------------------------------------RINL------------IKPVYV----------------------------

>refseqp|NP_659390|681_AA|Trichomonas_vaginalis_virus_3_Trichomonasvirus

M----------------------------------------------------------------------------------------------------------------------------------------------------------------------------------------------------------------------------------------------------------------------------------------------------------------DHVSD---------I-----------------------------------------------------------------------------------------------------------------------------------------------------------------------------------AYLNFLRWVLLPYNGQ----------------------------TLRPHPSVWRQTPYPE-----------------------------HVNLKFLNKEMEL--------------------------ELFPLK------------------------------------------------------------------------------------------------------------KAPQADLKVNCYARN-VLASTELTD-------------DLLKQSL-PIGLNNDSV-------------------------------------------CGIVIVLELLRIAGVPSKLLP------II--------GQAIA----------------------NKDPFIKELSDFNKMIGAT------TSRIANI----LTECNTLI---GRGVKSSDPSAD-----LYHRVAPEGNR------------HEAKIP-RHILIEAINKIYKNEMT----------DMPPPGDFKLHLITSPLWCKAGSHHHPH----------------------------------FAKYSSRLDFVMDVPADKIAAVPPSV----------FITQAEKL-EHG--KTRYIYNCDTXSYLFFDYILHYVECVWSN--ESVLLNPAA----------------MSVERF-------------SVLDYPEYCMIDYTDFNSQHSL-ESQKL-VFEC--LRPYL-------------------------------PSEM---------------------HPILD-WCIASMDHMEIGGQ--------HWLSTLPSGHRATTFINSVLNKAYLIPYI----------GDTTSFHC-GDDVLLCG-------------KYDYQTLIDTLPYELNKSKQSFGPN-AEFLRLHRR----A-GDVIGY-PSRAVSSLVSGN-WLSKTSWEWQP---------------------------------------------SLISVTNQCNVIISRSQLN--------IRFIPA---MQQELRNRYIDK-------MSEPFDVGSDYYVMPGCPCYS---------DAATTI---------------------------------------VPNVPQLECS-----DVPFSQAQKVFDTMRDICP--EFTTVND---------------VIDR-------------------VLARRTSNAVKNITYNV----------------------------------------------------------------------------------------------------------------CAPVAP-------------------QVCIAVNPAHYQFLLRKKYYP---------------------------------------------------REHIAPPGF------------DDS---------------TNSKLVFSTYDLA---------------PSIAMKSCAVLTPA------------------------------------------------------KIIC------------GHGLRSG---------------------------

>refseqp|YP_009507836|1481_AA|Trichomonas_vaginalis_virus_4_Trichomonasvirus

S-------------------------------------------------------------------------SAGSRRRPLCP-----------------LKGSNCAVALHVDGQLTRASR---------VPYRKLTPSHLNCSK-----------------------------------------------------------------------------------------------------RCARQLAVIYRY--------------------------------------QTLSPQLTEVSD---------S-----------------------------------------------------------------------------------------------------------------------------------------------------------------------------------DYLAFLRWVLLPYTGA----------------------------TNRPHPKRWPKPFYPA-----------------------------EVSLKFLDKKTEL--------------------------QLFPLK------------------------------------------------------------------------------------------------------------KAPQADLKVNCFARN-LLYSSPLSD-------------RILKQCI-PVGTNNDTV-------------------------------------------CGLVILLELLFEAGVPLDLLP------TI--------SVAIA----------------------KNDPFVKALSDFNKMTGAT------TSHIANL----LTECTTLL---GRGVTASAPNAD-----LYHRVAPEGNR------------HEAKIS-DDVLRSAIRTIYKQEIK----------DCPKPGDFGLHLLTSPFWCKSGSHHHPQ----------------------------------FPRYRNRLEFVMNTDPSAIMAVKPSV----------YITQAQKL-EHG--KTRYIYNCDTVSYLYFDYILNYVESIWAN--SHVLLNPDA----------------LNAEKF-------------ATLEYSEYCMIDYTDFNSQHTL-TSMKA-VFEV--LKEFL-------------------------------PSEM---------------------FPVLD-WCISSFDNMTIKDM--------KWRSTLPSGHRATTFINSVLNRAYLLPYI----------GTIVSYHC-GDDVLLCG-------------EHDYQHLITRLPYELNPSKQSFGPH-AEFLRLHRH----G-EKVIGY-PTRAVSSLVSGN-WLSTTSWNWQP---------------------------------------------SLLSITNQINAIICRSQLS--------ISRIRS---LAQELRFRYCPL-------LDNYIDPATTSFVAAGCPSYQ---------PTATMI---------------------------------------TPDVPHLDAE-----EVEFTQLHQLAEYAINTYP--WLNSVES---------------VNQL-------------------VRSRMRKPAARDIHYSV----------------------------------------------------------------------------------------------------------------LGPAIP-------------------LVSYHHHCDPMVVPLTRRYYP---------------------------------------------------RDHLAPPIT------------PQV---------------LPPQPVFCDRDLS---------------PIMALKIAPAGVAV------------------------------------------------------KVTA------------DRPIASA---------------------------

>refseqp|YP_001686789|540_AA|Botryotinia_fuckeliana_partitivirus_1

MEEFTQE---------------------P--------------------------------------------------------------------------------------------------------------------------TQHYVLAKGSHLIDALHLR----------------------PD-------------------------TGKGSTTSEDVLSSD---YRSPNLAEIA-RYGGYSTYSSNS---------------------------------------NTDPYVRETLK-------------------------------------------L----------------FSR--------------------------DTYEDIRGFTRRPEGTPGMYKA--------------LEKFSGEKNSFNDLSATQ----------KSSMRRA-----------IGKAKK--------------------------------------------------------AFKLPYK--------------------------------------REPLDWHEV-----------------------------GQFLRRD-----------------------------------------------------TSAGSTFMGQK-------------------------------------------------------------------------------------------------------------------------------------------------------------------------------------------------KGDVMEEIYHEARWLGHRMKQ-D-------------------------------------GKG----------------------------------------RFNPTKMRFPPCLAGQRGGMS---------------------------------------------------------------------------------------------------------------------ERDDPKTRLVWIYPAEMLTVEGFYAPLMYRDFMN--------DPNSPMLNG---------KSAQRLY-TEWC-------CKLREGETLYGIDFSSFDTKVPA-WLIRI-AFDI--LRQNIEFST--------FQGK----------PVSKKDAQK---------------------WRNVWDGMVWYFINTPILMP--DGRMF-RKFRGVPSGSWWTQMIDSVVNHILIDYLADCQD------VEIRNLKVLGDDSAFRS--------SDEFQLETAKLDCKPTGMVIKPEKCEKTAD------------PAD-FKLLGT----------------------------------------------------------------------------------------------------------------------------------------KYRSGHVHRD---------TDEWFK---------------------------------------LALYPE----------------SSVFTLDVAFTR------------------------LIGL-------------------WLGGAMWDKRFCEYMDF----------------------------------------------------------------------------------------------------------------FQSSYP------------------------CPEEGWFSKDQKRWLEVI-------------------------------------------------YSGKAPRGW----------TTKKS-----------------------------------------------------------------------------------------------------------------------------LFWRSIFYAYG-------------------------

>refseqp|YP_052856|539_AA|Penicillium_stoloniferum_virus_S

MEDSPFD---------------------P--------------------------------------------------------------------------------------------------------------------------TLLDVAVEESHLVDDSPLT----------------------PS--------------------------NRTRGASYGIISEK---FSSPGLREIA-RYGGYSVYSGQS---------------------------------------NTDAWIRTTLK-------------------------------------------E----------------FDR--------------------------SVYDDIYGYTRRPTGTLGMYGS--------------LLKFSEGKNSFASLNRVQ----------RKSMINA-----------ISKAKK--------------------------------------------------------AFKLPYQ--------------------------------------REPLDWHEV-----------------------------GRHFRRD-----------------------------------------------------TSAGVSFMGQK-------------------------------------------------------------------------------------------------------------------------------------------------------------------------------------------------KGDVMEEIYHEARWLGHRMKQ-N-------------------------------------GRA----------------------------------------KFDPSKMRFPPCLAGQRGGMS---------------------------------------------------------------------------------------------------------------------EASDPKTRLVWIYPAEMLAIEGFYAPEMYHAYMD--------DPLSPMLNG---------KSSQRLY-TEWT-------CGLRDGEMLYGLDFSGFDTKVPA-WLIRV-AFDI--LRQNIRWDS--------FRGE----------KVSKRDAQK---------------------WRNVWDGMVWYFINTPILMP--DGRMF-RKYRGVPSGSWFTQMIDSVVNYILVDYLAACQQ------CEIRALRVLGDDSAFRS--------CDPFSLDLASHDAECVNMILHPEKCEKTKD------------PTA-FKLLGT----------------------------------------------------------------------------------------------------------------------------------------TYRNGRPHRE---------TNEWFK---------------------------------------LALYPE----------------SVVPSLQVSFTR------------------------LIGL-------------------WIGGAMFDSRFCQFMEY----------------------------------------------------------------------------------------------------------------YQTCFP------------------------CPQEGWFSKDQRRWLQVV-------------------------------------------------YGGKAPRGW----------TTKRS-----------------------------------------------------------------------------------------------------------------------------LFWRSIFYVYG-------------------------

>refseqp|YP_271922|538_AA|Penicillium_stoloniferum_virus_F

METTT------------------------------------------------------------------------------------------------------PDLPFDLHTREA------------------------------------------------------------------------------------------------------YDYATFHRTLLHK---PGLSRIKEDRWVYKYNVEQTRMN----------------------------------------TDPFVRKSMK-------------------------------------------L----------------WDE--------------------------HAYHDMYGFTKKARLSNGL-DA--------------FQGFAKPQKQRSSMSPEM----------ASCYEKA-----------LEEARH--------------------------------------------------------VFTPHER--------------------------------------LTRLSVPNVCDST----------------------------------------------------------------------------------NLDSAAGFSFPGKK-------------------------------------------------------------------------------------------------------------------------------------------------------------------------------------------------KSEVVEEAFDVASYIAHFVASD-------------------------------------------------------------------------------------RKVFIPPAKLALRGHLSEI-------------------------------------------------------------------------------------------------------------------DEL--KTRAVWVFPFEISILEGKWALPYYKFLEQ--NVPEVHFGEGAMQR-----------LAKTLM-TDV---------ASHSECTEVTLDWSGFDTSVSN-WLIDD-AFDI--MFDSFDETQVEHDGNFV------------------LGGDHMA-------KK----------NEKVKKFLKTYFKKTKIMLP--DGSLY-KKFHGIPSGSFFTQIIGSIVNYLAVKTLDNYFS------WNARRFRVLGDDSSFLI----PFGRSKVDGVEISEKAWETFGFTLKLKKLRIANK------------QQD-RKFLGY----------------------------------------------------------------------------------------------------------------------------------------QCNAFRYERS---------TTEWLS---------------------------------------MVLYPE----------------RDVEFLEQSASR------------------------VFAF-------------------YLLGGCNDVTYCEFFHD----------------------------------------------------------------------------------------------------------------YLGRYP----------------------YIYGKELPLTRGLKRLFKFVF-----------RLTIDKLA---------FPDLSRFDPL-----KVPFSLSLGDKPFW--------------------------------------------------------------------------------------------------------------------------------------------------------------------------------

>refseqp|YP_009417301|811_AA|Australian_Anopheles_totivirus_Unclassified_Totivirus

M-------------------------------------------------------------------------------------------------------ADVSLLHLKFSSIVAKW------------------------------DGPKNFSNVRLVVKTSSPSDDRV---------------EVSPLGG--------------------------------------------------------------------------------------------------------NTKYVHSNVE-------------------------------------------------------------------------------------------YSAIRDRLKSKHCVEADGFS--------------FFKNYNP----------------------------F------------------------------------------------------------------------------------------------------------------------------------------------------------ALFKLQ---------------------------------------------------------------------------RW-------------------------------SKNDAGLGIHSTFMKPLLFYYKTLGCVK------------------NTEMTRDLL-------------------------------------------GGIISYVNDLTS----RAVGNG--------------------------------MFDYIDDVIRQFLAEVDKLTFVLCQQNAG------NSLVYSS----LHGLSTLGG--PRGWTEDSVLAS-----LKDWVTGERKFPYEN---------------DRFLNAKLDSWIDEWSR---------NIKNDDLSFSEFVSDPMRWATGGGAKKKQMNIRGRQV-----DGRNKWFW---------------------ALSGLSKGEDLYQVGLDE--------GNNAQVALK--EEA--KTRCVITTPQASYLRQCYMLYRFGNPSFL--RSTLSSP-----------------HLVSEL--------------SRSRKDHFICIDSSSFDHSVSK-KWVLS-VLQR--MADRCRGEL----------------------------------------------------RDVII-SEMDSLRDMSISYN----NHVLKYENGLLSGWRMTSLLGSMLSALVCEFINHSMMW-------KLPYIVQGDDIIMMC--------PRKVSAERVLECCSRFGIITNKKKTTIGRF-GEFLKYRYG----Y-GRVQGY-AARSVRSIFYANPWLDSTAVGAAS------------------------------------------------EVSGKWWTLLSRLMNSHNGGFKDEESLEWFMNNIASDVGGWLGGRVS-----RSALLDAIKTPVSLGGLGVFETADINLAN-NNGFITKITSI-VQEDSFGDSKFV---------------------SLFAPDVLSKVG---KVSTRF-VDVKKIAMNFGR--DLTEFKSKY-------HSIVNTAGRV-------------------VFDSGSNIFRTVLSEIA----------------------------------------------------------------------------------------------------------------SCRNYP-PIVDR------------------------LLSSVKGKCSSIV---------------------------------------RP-------RFLRNSNRW----------------------------------------------------------YDVAKWLTEITLKA-----D------------------CPPSLFVDTRYDNELV------QSLAGVAVTMFMNLSN-VTARSEYLISVFA-------------LFRFGHTKCILHAL-

>refseqp|YP_009230208|905_AA|Camponotus_nipponicus_virus_Unclassified_Totivirus

M------------------------GKNP------------------------------------------RIADTMARSAPIGY-----------------IMAGGKKLYSQTSGVCARW-------------FSDRGSCWCGRCR---------------------------------------GDAHCAPAGNRLI----------------------NGKDGYQSPGSRN---PGGTRGEERA-GTKPNTAGPQDP----------------------------------------TDSETECQEGGGNGRGGENRG-----DEDPYSGGPAPGT--------------------------------DEGTRDSGREPG-----------KRLAGLYHKTQGDKDRVRACPESLVASRQQVYSSVYHEFEHVIRIHEPEKLRPTPG-------------------------------------------------RVFKLT--------------------------------------RYTGRVP-------PDW----------------------------ILPTVTCSYPL----------------------------WLYSRA-----------------------------------------------------VEATGQFASSK--------------------------------------------DIQLIGLKIQSELNK----------------------------------------------IRDV--------------------------------------------------RLHS------------------------------------------DAARQMLKNISNYCFPLVFS------RGPVYML----FVHLNWLTA--KCPYTDGQILDD-----IGNWVSDRKEN-----------KEEKKLD-ETVCNRVLDRVFHQWYT---------GESTGHLDFKSYCNDFTRWGTSGGSPKVKLDDIDYR---------SKWAW-------------SLYHATDEKTGDLLPEFDLYERAKEE--------RTTSTIALKE-EPA--KTREIITTSMASYLRQSYLMYRWGKPKIPS-----------------------PISVGTWL--GRF---------ERASPKWFGSIDGERFDHCIPK-EFIMG-IVAR--LGEL--------------------------------DEQT---------------------MFAAK-EELEHMRGLRLQ----WGEHMWDWRGGLLSGWRLTSVIGSLVSCCVAEYILEKSGK-----LGAVQYGVMGDDLILYSY-------HEEIPSDEMVALYTGFGLKANLAKTSSGRI-GEFLKRIIS----K-GGTWAY-PALGLRSICYANPWLDHYTYNEET------------------------------------------------EVSTCWLTLFSRLLPHC--CVRKEKQLSSF---IRAGCVSNLTMLFG-----KNKWDDWLCTPISAGGGGCMEWSKLESWCNLDKLVDQVAGG------------------------------T---RVFFKSLFGTIPY---------RRVMKYTHTMRKL-DLHTILYW--------------KQQLGG-----------------AGKSPPDTYFRHEVNITQT-----MYDY---------------------------------------IFGRISLSNL--------------------KGSLNFSLPRG---------------------IRISSPQRIVTFLLQGVREYSGIT------------TIQHTRDAMQ-----------------------------------------------------------------------------------P----------------------------------YADIGAHVVRAVSA--------------------------SKRFTNIRY--------------IAAAVTLYM---------SEILKDVYIPFGTW-----------------------

>refseqp|YP_009143313|659_AA|Camponotus_yamaokai_virus_Unclassified_Totivirus

M--------------------------------------------------------------------------------------------------------------------------------------------------------------------------------------------------------------------------------------------------------------------------------------------------------------------------------------------------------------------------------------------------------------------------------------------------------------------------------------------------------------------------------------------------------------------------------------------------------------------------------------------------------------------------------------------------------------------------------------------------------------------DIVKMVLKIQTIIAG----------------------------------------------YNID--------------------------------------------------ELYA-----------------------------------------SDAWRQILKAISGRFFPDVFS------KGPCYML----FVHLHWLTA--KCPYTTEQIVSD-----ITEWV-DSKME-----------GRTKEVD-QTICDKVLDKVVLEWYK---------GEAGGFLSFKDYCNDYVRWGTSGGAPKAEVLGQEYR---------SKWAW-------------AISHATNAD-GSLKEDYDLYEEALKE--------RTNSVIALKE-EPA--KTREIITTTMASYLRQSYLMYRWGKPNIPS-----------------------PISTATWM--GKF---------ERASPSWYGSIDGERFDHSVPK-QFIIG-LMNR--LGKL--------------------------------DSET---------------------RQVAQ-DEIEHMNELKVI----WGEKEWKWEGGLLSGWRITSIIGSLLSCCVAEYIISKTHM-----EGALHYGVMGDDLIMYSY-------REKLESAKMVELYREFGLKANLAKTTSGSV-GEFLRRVLS----K-GGTWAY-PALGFRSIVYANPWLDHYTYNEEA------------------------------------------------EVSNCWLTFLSRLLPH---CYKKTGDVVSL---IMRRLKGNLSMLFG-----KLDWDSWLRTPISAGGGGALEFSDVSRWSYLDKLRDTDVLS------------------------------N---KAFMSSVFGLTPY---------KRILKPITTMKKL-DLTTVIYW--------------KNQLRG-----------------VDTSPHSSWFKHETNITRL-----VYDY---------------------------------------VFRKIRLSEL--------------------RGGLLHALPRG---------------------LRVTSPQRVVDFLMRGTKEYTGIT------------TIQHTKDSMQ-----------------------------------------------------------------------------------A----------------------------------YSDIGAHVVRAICS--------------------------SKRFTNVRH--------------ISAAVTLYM---------MEILKDVYIPYGTW-----------------------

>refseqp|YP_003934934|761_AA|Armigeres_subalbatus_virus_SaX06-AK20_Unclassified_Totivirus

MKDIL------------------------------------------------------------------------------------------------------CVVSQELKEEMKYYQH---------------------------------------------------------------------------------------------------------------------------------------------------------------------------------LDNILFTNIMIYGHIHGPS------------------------------------------------------------------------------------------------------------------------------------------------------------------------------------------------------------------------------------------------------------------------------------------------------------------------------------------------------------------------------------------------------------------------------------------------------TIRELRELG-----------------------------------------------------------------------------------------------------------------------------------MFNDFETFVTMAGKISGHFKRYPIC------KDETKQR----LCEIQCLTGYLQNDPPGWDFEKE-----FVSLAEGGYEHGLV----------------GEDWPARFKELNDKVMT--------RQPMPDFISFEDFI-RDGLWITAGSSSIGKVEWTKTD--------------DK-----------GKFKARKNMLTELYTDEDLIEIVNNWDGIL------RSRVFTKD-ELS--KRRLAVASNIEAYLSEAWILHLFGHGFKNY-EYITLDESPKRQHE-----------RTSKL--INL----------LRNGSFCLPFDFKGFDHQPQIKAEVQV-ILQK--IVDHVRTKV---------------------------PKDKLA------------------TFNSIAVRMVESYAKGEIINP--MTMEVLIQIGGIPSGVRPTSLIGNVWNGDMTTYARELTKLMMRR-DEIEEIGIKGDDTYIAS--------KNPVALIIFRLAYAAVNAIGLDSKFGISQNICEFLRNEIS----I-NGCQGW-SNRAIPSLSQRK-PWNAQPWSPSS---------------------------------------------EVATVANNIYLLERRLKRST-------------P-QLHQANKIKWSKYTNQS-------YHWLHLPVRLGGFGLYPFEGWE----PNGKLP---------------------------------------LVAKPF----------------ITVNNLKISREYV-PWAKLTPE----QNILYAQEDFNSKI----ATDDIP----GPQKYYSRDFVNILRTKTFSWTKEPTIIRIFPPKVQRPPVAEHVWWP-------------------------------------------------------RDRFVN-------------------QKS----LDPTMP------IFA--EFIRQHQ------------TLKRASGRMNIK------------VEPLPALAKK--WYPHLWSS-----------------VEYFEANGW------------HRT---------------DAINLAIGNIPTEPT---------KILHPSLTAFVKESVKRNNFRYW-----------------------------------------KGRRNIALNLYTITT-------LAVHEIQA-----TGGKHLYAY--------------

>refseqp|YP_003289293|621_AA|Drosophila_melanogaster_totivirus_SW-2009a_Unclassified_Totivirus

M------------------------------------------------------------------------------------------------------------------------------------------------------------------------------------------------------------------------------------------------------------------------------------------------------------------------------------------------------------------------------------------------------------------------------------------------------------------------------------------------------------------------------------------------------------------------------------------------------------------------------------------------------------------------------------------------------------------------------------------------------------------------------------------------------------------------------------------------------------------------------------------------------------------------------------------------------------------------------------------------------------------------------------------------------------THQEHKKYISFEKYV-KEGYWITSGSSSIGKVNWSYDG--------------DL-----------GKFKARKNMLLDLYTPDEIYKMAVEWDGKL------ENRVFIKD-ELA--KRRLAVASNIEAYLNQGYIFYLFGHGFKNW-KYITLDETPGETHL-----------RNCET--IKA----------LKDGCFALPFDFKGFDRQPTI-WEVKQ-ITKR--VIDQIKYLV---------------------------PPQDRH------------------LFTKIAAKNISCYDNNYLYSP--LTKETKKQTNGIPSGIRPTSFIGNVWNMITTDIARDQTSLILGK-DNIRLIALKGDDTKILA--------KDFMTCLVFRYSYQAINAVGENSKFGIMQNCCEFLRTEIS----T-QGVRGW-TNRAIPSVTQRK-PWNPQPWTANQ---------------------------------------------QVETTANNIYLLERRSKKQLD--------------WLHQANKIKWSKYTGQS-------YHWLHLPKRLGGFGIYEWKGWK----TTSKLP---------------------------------------LADIPT----------------FDVPGLFSENINL-TWKQMTDE----EKTAYQQVEFSMKI----AADDIP----GPQKHISSNYIKNLRKLKPVWEK--TVVPTYRFYKTEGPTCENNIWP-------------------------------------------------------RRMIQS-------------------HEA----GPNGMP------IFS--EFIRQHQ------------IAKKAKIEIP----------------SLKECLAK--WYPEAYKT-----------------MTEYERKGW------------HRT---------------DAINIATGKVPLEPT---------GILNPILTPWISKIVVKSGFSFW-----------------------------------------RGRENIALHLTSVCK-------QAVQYIQN-----EGGNYMYAF--------------

>refseqp|YP_009072448|791_AA|Leptopilina_boulardi_Toti-like_virus_Unclassified_Totivirus

M---------------------------------------W--------------------------------------------------------------------TPKLLRDRMEEA--------------------------------------EAELMDEVGPGLDCGCGT--------PGWNMAMPK--F---------------------C-----------------------------EKLLKYRLVSHKTCH-------------GCVL----------------GKGVLEDYVHCVIR----MSGERAGVEM-------------------------------------------------------------------------------------------------------------------------------------------AEVFLAA-K-----------------------------------------------------------------------------------------------------------------------------------------------------------KRWPRA-----------------------------------------------CRHGVN---------------------KYW-------------------------------------------------------------------------------------------------------------------------------------------------------------------------------------------------------RRRTGLLKMGGTR------VTPYWRE--ECYWEL-------GQGYVPYKPMEDMGPEAIRWLCKDTVLG-----------GPVGEDNYLDWFHREVEDFMRTEFK-----------MPEHQPTIDGWVATGKWMEGKSGTGG----------------------------KVGVTIDGKRKMTRRT----KPLAGVLMWDAEVGMELTASSREVMHILQKS-EAG--KVRSVVKTGDKVNRKMNYLSGYLEDGLHGS-PLSTLFAGEAGNER-----------IDFDLI-DAV----------RDESTWKVPLDQGAFDEKQSK-MSIAV-ALHA--VGMALEERG--------------------------MNGDGCA-------------------VWAAL-WDSLFVRGALVE----WADESRPWKNGLPSGWRWTAVLDTILNVCSFRVIRKISEIRLGKPFWVGHFYAQGDDVIFAA--------RDLGGIRLIIDTYGKLGYEVHPYKTYISRGRGEFLRRSYE----A-IGVTGY-LARTMHGLRFKN-PIQDDPLSLTE---------------------------------------------RIYSHMMQWHLAMLRGGVP--------EVVVQM---LMEDLRGMRISTKKAAG--------LFLTPNCLGGGGVDP---------ASAFGSWIEKH--------------------------------------------------------SDGNWYTLKVTR--EMRCVNVRLGGWKERLRRYDELMSGT-------------------ARDALMRSFALSWGLKD---------------------------------------------------ADITGLHDVTFT---------------KIKGVVPIPPTSPF---------L--VPKVGDLWNMDDVPVQIRDAV-----------------------KREAIRRGST----------------------------------------------DRWLTVRGKEIARWAYD------R--------------------MSPRVVKGFLLNEWNAPCPITDRVGTRYGVKIKRWANTMIRSALH----------------------------------------VRNIGMRQLESHLYWIEL-------QVREKLKL----------------FGASQLLAQ---
