# Supplementary material for: Detection and Molecular Characterization of Novel dsRNA Viruses Related to the Totiviridae Family in Umbelopsis ramanniana
Source: Front Cell Infect Microbiol. 2019 Jul 11;9:249. doi: 10.3389/fcimb.2019.00249 (PMC6644447; doi:10.3389/fcimb.2019.00249)
Supplement: Supplementary file 8 [file Data_Sheet_3.docx]

**Supplementary Data S3** Clustal Omega alignment of selected RDRP amino acid sequences used to determine the conserved motifs.

CLUSTAL O(1.2.4) multiple sequence alignment

UrV3_RdRP ------------------------------------------------------------ 0

YP_009333150.1 -MPRGDGSGTAPRCPLLPPPYGAGADGGPESGLSAGLRRASEDPPKVGDLCAVGFGVTPV 59

BAU79517.1 ------------------------------------------------------------ 0

AAB94791.2 ---MSDPQE---RSKAY------GLLGERLYA---VASANSHMLAGYDSL---------- 35

UrV2_RdRp ------------------------------------------------------------ 0

YP_009182167.1 MAAHLSISE---RRAAL------GHLGDVFYG---MLEKAEFPAEKFRELRATE------ 42

NP_620495.1 ------------------------------------------------------------ 0

YP_009342428.1 ------MQE---RDSFTALDICDGMREGREHT---FAVRSGHVPTGLRITCNGTYLGVPF 48

UrV1_regi_RdRp ------------------------------------------------------------ 0

UrV4 -MPMKKPKG---RVTVTLSSIYDNKDGNFKNV---VPRNSDPMPLTFRKSNSGGHILVPF 53

UrV3_RdRP ------------------------------------------------------------ 0

YP_009333150.1 RGDAGPTLPPGVGPHRLANN--LLREAERM------VKTGDRDSS--------GAGVTRW 103

BAU79517.1 ------------------------------------------------------------ 0

AAB94791.2 -------------------------DFTARL---VRLTGE--ATALKAVD----PLLPCA 61

UrV2_RdRp -------------------------------------------QKHPHYD----AAVRAA 13

YP_009182167.1 ---QLLLLS--VGGGVLNSSLALAVEFNRRVRSRVGLKGQPGLKTGVVVD----PLLRVA 93

NP_620495.1 -------------------------------------------------MTILSPAARRQ 11

YP_009342428.1 KQAELLLID--RLPAKLHEC-NVAYDYYGTVVSARLVPGRDATYVYYYVDQDIQPVTQNL 105

UrV1_regi_RdRp -----------MLDEAYPEG-DCMYNYYGSVFNCLMLPSKKRTYVYYKVDQLLRPRSRNI 48

UrV4 RRAEYVLID--VIEGDYGCE-TISYSYYGSVVKADTICRGGMTYVYYHVDQLLSPMSRNI 110

UrV3_RdRP ------------------------M--APDPQPVREDVVEV---QNVEWTREALKGAYER 31

YP_009333150.1 GGLLRALYFGGAAGAEDYEAAGLAV--WPPPQTVAPRVAADASLPDM--SIEAL---KER 156

BAU79517.1 --------MPQCALVEDYTTLRHE-------CTITSYVAMTPPLPQHWPTLEDCKNNYVA 45

AAB94791.2 VSLLFMDFPLQLPCTPE-ETLRLVRRAYDPNTLEEVDYSTLSGYATQFARVKG-QRGRWR 119

UrV2_RdRp ITLLLTSFPVQAPMSSI-DIIGLARLAFPVGTYS--------RSPHS---PP----TN-- 55

YP_009182167.1 ASMLCARFAVQVEMTDS-NVRRLVRLAFPEALPPL-----WSGVPAE---VR----GK-- 138

NP_620495.1 AS---ATY-SQVAG-------------F---------CYNTPTVMDSLANILDVDRNIRP 45

YP_009342428.1 LGMLSRHFLGDFSG-------------Y---------YNDWCSLDNVFYSLSKCDNPVKR 143

UrV1_regi_RdRp LAILSRHYMDNFNG-------------Y---------YNDWCSTENVFAGLSKVSEVNFR 86

UrV4 LGILSRHFMDDFTG-------------Y---------YNDMCSLDNVFLGSGVA-SPQQR 147

UrV3_RdRP ----------------PPRQYSDM-MKVSMLTVYD-IGLFDEP---LVRE-------AAE 63

YP_009333150.1 ----------------HPQGYGGS-GAVIQLTLTTTFKALGRGPRRWECE-------LMN 192

BAU79517.1 ----------------FPAGLGGP----IKFTITTISELFPNPIQPTTAL-------ILH 78

AAB94791.2 HLGHLVCNDKAFRERYFPKKKHAAAAIKTNIRLGPLA--------------------RAW 159

UrV2_RdRp LPLRAI-LKTPFLMQYFPFKYHPAATMKTNVRMSDLL--------------------RSL 94

YP_009182167.1 VSLSALAARADLREVCFPYKTVPQATRKANVHLASLL--------------------TPK 178

NP_620495.1 KHFKGL--------RLYTRSKVTAQ-HHTHLRPDELVEAAAKVSPRRKYYLMCVVELLAN 96

YP_009342428.1 HTIKTI--------RDLPKPKISGA-HHIHYTASEVWSVLDDRGKDAAR---HALRLPAD 191

UrV1_regi_RdRp HSIDKL--------EQLPIAKISAA-HHIHFTAAEIWQCLDSEQRDKAK---HALRITAQ 134

UrV4 HTLHSI--------KNLSKAKISAE-HHIHYTAEEVWSTLDSAQRSKAE---HALRITNE 195

UrV3_RdRP AQRGQLEYAVAALCLWLTT----DVAKY----LCAELPVHRVPLSKW--PGSVKAF-AND 112

YP_009333150.1 ANVGHPEWSVGAVILFFSA-LEPDVVDW----LVEEARVHLIPLATW--PECLRDW-FTA 244

BAU79517.1 FNVGAVEAQVAAMLYWVHT-APRKAVDV----LT-SHGWFHKPLKQW--ADVFKDS-LDT 129

AAB94791.2 AA-RYGLAALGSHLAYMVG-MPNDRACATLLLAQTYKARFGSEGVAW--AIASVRQPENA 215

UrV2_RdRp AS-QGILARFETMIYALAGRVSDDQACSAILYASGLAPHLGPYAYEF--ASCCVLSPKNA 151

YP_009182167.1 A--RELVGGLDRLIGWLAGRCSDDQVCSAIIYAHALGSRWGPGAAEI--AARYILDPEGA 234

NP_620495.1 LQ-VDLEAAVATILAYVLT-LS-----------EKFVPIFLDSRAIWVGEPGPDALTARL 143

YP_009342428.1 AT-T---SFVGGVMLWLAS-LP-----------TELLNPIIESDLLD--AEDTIEFGKKA 233

UrV1_regi_RdRp AT-T---TMMGGVMLWLAM-LP-----------TELFEHFVKTDILD--ADSMVEFAKRA 176

UrV4 AT-T---TMMGGVMLWLAM-LP-----------DELHKRFVNTDILD--ADTMVEFARRA 237

. .

UrV3_RdRP ARRLGQVFGRGPQEVAMAFRLRRLVSLAGRSTADADWEKEVAERTQLTTAKRA-FADGEV 171

YP_009333150.1 LRRCPRLRGMPHVSPSAVLELRKMLNCVLRSNDEADWKHEYTRKCAEQAIHTGVGANGML 304

BAU79517.1 MRRQATVPGSLN-DHETFSALRKIMSLTYRGTEEPDWDAEMSRMRENMPIRSFNKLPRAL 188

AAB94791.2 KGLSNALKALGSNTSEPGALFVEANTLQGRYDRTLDMDHEVESRCSPAAIADQV------ 269

UrV2_RdRp KGLSTALKALGANSHPTGALLIEADTLQGRGVGSVDLLQEAKYRCDPSLVAASV------ 205

YP_009182167.1 VSVGLVLKAMGANSGPLGAALVEGKSLQGRGVGSLDLAKEAEQRCDPDWVAGKV------ 288

NP_620495.1 KASSGQIKSIHTADYEPLTELFELAVLMNRGVGHVSWQAEKDHRLNPDVAVV-------- 195

YP_009342428.1 KKLSVTAKSFQNIVEADLRPIFEADVLVNRDVGEVDWAGEKNNRCKPNLASV-------- 285

UrV1_regi_RdRp KKLSVQAKSFQNIVEPDLRTMFEVDVLVNRDVGQVNWDAEKANRVTPDLVNL-------- 228

UrV4 KKLSVTAKSYQNIVEVDLRTVFEVDVLVNRDVGKVDWEGEKQNRVKPDTVNI-------- 289

. . . * . *

UrV3_RdRP SSAAYR------LIRDKVLHRIAVQVVNSLKKSGGSFDEYFEQRWWNTPRGTTSKGGDVK 225

YP_009333150.1 SQAVWY-NDLAVSIKEYVDGTINATVE---AREPETASEFWRLRWERGAAGSSSER-KRL 359

BAU79517.1 -HLNYN-RELWNAIKEVTATTVHNVVR---NTRQRSMDEWWATRRAWAPSGSSTNR-RLL 242

AAB94791.2 --IPYT-DELGACIDFILDTELGGD-----TIELPDEDEWWTSRWLWCVNGSQNALSDKA 321

UrV2_RdRp --IDLDPELLRLAIDDILDEELKVD-----KVEIPTPQEFWQRRWLWCVNGSHSRVLDRR 258

YP_009182167.1 --LHCDPEELRLVIRQILSEELKGR-----EIVFDTPEQFWERRWQWCVNGSHNRTWDAR 341

NP_620495.1 -----DQARLYSCVRDMFEGSKQTY----KY-PFMTWDDYTANRWEWVPGGSVHSQYEE- 244

YP_009342428.1 -----SPGEVYARTLALFTKEDDEK----ALPRRLEWEKFWDARWQWSASGSIHSQYQE- 335

UrV1_regi_RdRp -----NNKRIYDAAIKMFSRVDATK----QKPRRMSWRDFWMSRWQWSASGSVHSQYAE- 278

UrV4 -----SKKTVYDEARKLFSRTDNTR----LKPRKLKWEDFWKTRWQWSASGSVHSQYAI- 339

.: * *:

UrV3_RdRP RQLKNADKHLDL--QMRPIKPTVMELYSKPGLLQDLRGLPYCVARGSTKPEPGLKCRALL 283

YP_009333150.1 ADLCAADERLGG--QARANKKAVLEAMTDADFETMWEGLPSYVARGSTKPEPGGKQRALY 417

BAU79517.1 DDYKKREPRIKS--ADRASKKTVVEALPDDYMETSLARPPISLARRSKKSEAGWKARALY 300

AAB94791.2 LGIKNK------SGQRYRRMAAEEVN-----NNPVPAWNGHTSVSPSVKLENG-KDRAIF 369

UrV2_RdRp GGLDTRS-IFPGVDRVYRRMYAEAQS-----EEPLTSWDAQVSVSASEKLEHG-KTRAIF 311

YP_009182167.1 AGVDLPA-SMPGCDRFYRRAFSEVCK-----VETLTGWSGEVLAGVSPKLENG-KTRAIF 394

NP_620495.1 ---DNDY-IYPG--QYTRNKFITVNKMPKHKISRMIASPPEVRAWTSTKYEWG-KQRAIY 297

YP_009342428.1 ---DLDS-LPKE--RELKNKFIALSMQGDMPFEHFLNRKPEIVAWSSVKYEWG-KMRAIY 388

UrV1_regi_RdRp ---DLKD-LPKE--RELRNKFIQLCQAGNYDANHFLQRRSEIQAWSSIKYEWG-KMRAIY 331

UrV4 ---DIQN-LPKE--RELRNKFILLTQTPYREFDFYATRKPQIQAWSSVKYEWG-KMRAIY 392

. * * * * * **:

UrV3_RdRP AVDDRTAIVAGYASSGIETTTKEGGMVLRQDP---ADVAEWVSFDL--GPGVWRVSNDYS 338

YP_009333150.1 ATTDECFILSAYGSADLEKYMNIEGIRAKQTP---ADVVEWVKQGMDMAPQARWVSLDYS 474

BAU79517.1 ASDDVPFFIASYASLDMEKNMNHDGMNPRQTP---DDVAAWVLADARTTMSDVWLSLDYS 357

AAB94791.2 ACDTRSYFAFTYWLTPIEKKWRGARVILNPGEGGLYGTARRIRGS--QTSGGVNLMLDYD 427

UrV2_RdRp ACDTLSYFAFEHLLSPVEKAWRGVRVVLDPGTMGHLGVANRIARI--QI-GGVHVMLDYD 368

YP_009182167.1 ACDTLSYYAFEHLLGPVSAAWLDRRVVLDPGRVGHLGMAERINRT--RDGGGIDVMLDYD 452

NP_620495.1 GTDLRSTLITNFAMFRCEDVLTHKFPVGDQAEA--AKVHKRVN-M--MLDGASSFCFDYD 352

YP_009342428.1 GTDLTSYVLAHYAFFNCEDTLPTDFPVGEKARP--SFVSARVA-A--ILEGTVPLCVDFE 443

UrV1_regi_RdRp GTDITSYVLAHYAFYNCEDVLPNEFPVGLKARP--SYVSAKVQ-S--VLERKVPLCVDFE 386

UrV4 GTDLTSYILAHYAFYNCEDTLPNEFPVGNKARP--SYVSAKVG-A--ILKGRIPLCIDFE 447

. . . : . *:.

UrV3_RdRP NFNGLNSLRSMQLVDLHLAQEWRRVP--ERWAEEKALASEWVAASYLNPYMKTPLG---E 393

YP_009333150.1 DYNWEHTTAALMLMELCFAAAWCRKGGDREWGADKTAAALWSMWAHAAKFTVVPKVDA-S 533

BAU79517.1 DYNKEHRNTELALLNLGFVLAWAVAPVDPSVRVDKMRCALWTAKAHLCAFV--SDGEH-T 414

AAB94791.2 NFNSQHSNETMAALYEKALSRTNA-------PA---YLKKAVAASVESTYIHYKGR---D 474

UrV2_RdRp DFNSQHSTSSMKVLFQALTERIGY-------PP---DLAGNILSSFDNMWLYCKGT--LV 416

YP_009182167.1 DFNSHHSNTSMRILLEETCAAVGY-------DE---ELGRKLCQSFDNTWVKTPAG---L 499

NP_620495.1 DFNSQHSIASMYTVLCAFRDTFSR-----NMSDEQAEAMNWVCESVRHMWVLD-PDTKEW 406

YP_009342428.1 DFNSQHSNDAMEAVVQAYIDAYSQ-----YMTPEQVQAAMWTRESISNTRVIDNMGTKTS 498

UrV1_regi_RdRp DFNSGHSNQAMQTVIQAYYDVYSA-----GMDDDQKRAILWTRDSVARTQINDNMGTKTS 441

UrV4 DFNSGHRNDSMEAVLQAYIDEFHE-----DLDPMQLSAAEWTKQSISATIVNDNMGTKTQ 502

::* : : : :

UrV3_RdRP TRVVSGLWSGHRNTARDNTFLHLVYLECIKSVMRALFGQHAKHGKVRLCGDDETLGYDEW 453

YP_009333150.1 WRFFGGLFTGCRNTARDNTLLHGAYSKTIEKYLGMVDPGKY-LMHKNYTGDDEDSVLPDW 592

BAU79517.1 HRIFSGLFSGHRDTARDNTLLHAVYSKLARKVVSRITGARCEPRFIAMCGDDEDACFNSD 474

AAB94791.2 RHVLGTLMSGHRATTFTNSVLNAAYICYAV------GIPAFKRMISLHAGDDVYLRLPTL 528

UrV2_RdRp GRAEGTLMSGHRATTFINSVLNAAYIRLSL------GKEVYERYKAVHVGDDIYMNAPTH 470

YP_009182167.1 SRVRGTLMSGHRGTTYINSVLNAAYIRLAV------GRAAYEGMVSMHVGDDVYVNCPTP 553

NP_620495.1 YRLQGTLLSGWRLTTFMNTVLNWAYMKLAG------VFDLDDVQDSVHNGDDVMISLNRV 460

YP_009342428.1 YKTKGTLMSGWRLTTFINSVLNYVYTQAL-------ISGSHAMSRSVHNGDDVLLGIRNF 551

UrV1_regi_RdRp FSTNGTLMSGWRLTTFMNSVLNYIYTQQL-------LENCGEHVNSVHNGDDVLLGVSNF 494

UrV4 YKTNGTLMSGWRLTTYMNSILNYIYTKLL-------TKDTESTYQSVHNGDDVLLGVRNF 555

. * :* * *: *:.*: * ***

UrV3_RdRP CAAVLHTVVADELGFTSQVSKGMLSRKHDEFLQLLRQPG---KVPSYPIANTILTFCSGN 510

YP_009333150.1 VAAANYLVLHSLAGFAIKPAKQMCSRETHEFLMRLAMPA---VLPTRPLGTALAIFTSGN 649

BAU79517.1 IMAMMYLHVHSWANWTLTDRKQMLGNAYHEYLQRSAFNR---QLPTKPLATILETMATGN 531

AAB94791.2 ADCATTLNNTKRVGCRMNPTKQSIGYTGAEFLRLGINK----SYAIGYLCRAIASLVSGS 584

UrV2_RdRp SDAALVLTRAQALGCRMNPAKQSVGTVGAEFLRMGIRP----GGAVGYFARSVASAVSGN 526

YP_009182167.1 EGVEELVDRCAAIGCRMNPTKQSVGKVGAEFLRMGIRR----EGAHGYLARSVASLVSGN 609

NP_620495.1 STAVRIMDAMHRINARAQPAKCNLFSI-SEFLRVEHGMSGGDGLGAQYLSRSCATLVHSR 519

YP_009342428.1 KIVRDIVARADKYNVRLQRTKCAFGGL-AEFLRVDHMR----GDYGQYLTRNIATVMHSR 606

UrV1_regi_RdRp DIARRTVYNAEKYNIRLQRSKCAFGGI-AEFLRVDRVR----GDFGQYLSRNVATLMHSR 549

UrV4 DIARRAVFNADKYNVRLQRSKCTFGGI-AEFLRVDRVR----GDFGQYLSRNVATLMHAR 610

. * *:* : .

UrV3_RdRP WYKDPVRDLNTTVADVSDHLWDLVLGGVDPDVCQRLGVYVLDYLMQVKRSDGS-LFPLEW 569

YP_009333150.1 WYKDVHVYYDSIISGVSSNVWELVARGLPLVVGRRLAAATLNAQMRLKNADGT-RTLLEW 708

BAU79517.1 WYKRSATWYDSVISAVSDNCFEMVRRGVRLENAQALARKLISAMMKIKRSDGT-IKKLEW 590

AAB94791.2 WTSLDELQPLNALNGAIVQTRSCLNRGAATGLPELISAS-FVGLRG---------FK--- 631

UrV2_RdRp WVTEARQSPIDALKTAIANVRTLCNRSRSEAFPTLLAGP-LSRLTR---------VP--- 573

YP_009182167.1 WVNEKVMDPEELLSSMVGTVRSLINRSGCETVPQLLAPA-VSAVTR---------IK--- 656

NP_620495.1 IESNEPLSVVRVMEADQARLRDLANRTRVQSAVTAIKEQLDKRVTKIFGVGDDVVRD--- 576

YP_009342428.1 IESKIAVSAVDVVQAMEDRLREYLQRGGVWDYAVQLRELYYDRMAPIYSLRPQDLYD--- 663

UrV1_regi_RdRp IESKVALNVVDIVEADEERFREFVRRGGDEAVVSRLRHLSYKRTAKIYDTELSTLYM--- 606

UrV4 IESKLALSVVDLVEASEERLREFIQRGGSPKTAARLRSIAYDRYSKIYETDTATLYR--- 667

. : :

UrV3_RdRP WDFRGSGIPGGHPLWGGFETPAPPQI------------KVKL-P-------TIKLPMAAT 609

YP_009333150.1 WRYRHGTGAAEHPLWAGTPGDWEPVP------------EVVS-P----LEVHPDAPTHAT 751

BAU79517.1 WTYRDPS--ASHPLWMSQEPTATPPI------------TSAK------PRPSYSAPTHAT 630

AAB94791.2 -------RRDLLELLTGVATIKPGPVYTS-SCVIREYVVEQPPPPQ--FDVPPGAGMHAT 681

UrV2_RdRp -------RRLVHALLDGSKAIEGAPIFHS-DGMIRTITEVPPPPEQMAERLPPDWPRHAT 625

YP_009182167.1 -------VARCVSLLSGQSALEGRPVFNPRDGLIRTWALRVERPA--SKVKAKDLPSNAT 707

NP_620495.1 -------IHTAHRVCGGISTDTWAPV----ETKIITDNEAYEIPYE--IDDPSFWPGVND 623

YP_009342428.1 -------IRTAHRVVGGIAEDVSARV----DKFIRTSSESIET------TLPDTLPGVDA 706

UrV1_regi_RdRp -------IKSSHRVVGGISDLDNAPV----DCIIEKDKTGKIL------PLPDQLPGVMD 649

UrV4 -------IKYSHRVAGGISDGLGAPI----DQVINKDQVGRIA------ELPDYLPGIAD 710

: .

UrV3_RdRP QDSVKREWPVWERLEKHRLAET------------------------MNERAWSSYRVVAK 645

YP_009333150.1 NAWVKEKQRSLGLPATKEWDVV------------------------RKTAVAASFGKMYS 787

BAU79517.1 DAYIKHAYSILTHLEPKKLVLY------------------------KNEMLCESYGSIHT 666

AAB94791.2 MSYLARHTTLVEAQALEIARPAI-----------------------KSLMLSSSYGKAGP 718

UrV2_RdRp NDYVVNCATAVEQFALKEAGRSV-----------------------ASAMLIASYNKALT 662

YP_009182167.1 DDYLAKAASVLELRGAAMSTVDP-----------------------RAAMLDSSYRKTLA 744

NP_620495.1 YAYKVWKNFGERLEFNKIKDAVARGSRSTIALKRKARITSKKNEFANKSEWERTMYKAYK 683

YP_009342428.1 YARLLKKVLELEVPVSVVSTRIRNATLNAVQLTRKTVT-VEKTLNVQRYMVYRALYRAYA 765

UrV1_regi_RdRp YAIMLKKSLELTVSTREVYKRVYNATLNAVQLVRTSVK-HTYNENIRQYEVFRALYKAHS 708

UrV4 YSNVLKKSLNLNMEVSKIAKRIYSATLNAVKLERTKVH-TEVPENIEQLKVYRALYKAHS 769

:

UrV3_RdRP HWLQEEYDKAAQEEWPARRDCVKV-HIPVVRREVPTNRWRA--------------I---- 686

YP_009333150.1 RHAAHMHEAEVRRVWPRRESHPHGLDVPGPPKPDQD--ELK--------------NLMYV 831

BAU79517.1 SWRMDLNRQAAEQLWPERHSEPLLLEAPQPPDAREKQDWVI--------------QGMLC 712

AAB94791.2 GAQTRPHV-----PMPKLRRQP-----PRVAVGFSMAHELTGRGVKEGCLSGHPLLRLFE 768

UrV2_RdRp SVAAPPAY-----P--RFKANR-----PRAPVGSQPALALLYTRKTSGVLQGYPLLTLLK 710

YP_009182167.1 GDTDPSKI-----KL-TLRSCT-----PVLARGATNARDLLRRPCPPGALERYPLLQLVK 793

NP_620495.1 GLAVSYY---A--NLSKFMSIP-----PMANIEFGQARYAM---------------QAAL 718

YP_009342428.1 DVTNNPMFGKA--MLTGFIFDV-----L---TQNAQLSAVA---------------QLVS 800

UrV1_regi_RdRp DTTDTPLFGKA--MLTGFVFDV-----L---NKSKNMTTLI---------------RMLQ 743

UrV4 DATDNAAFGKA--ILTGFVFDV-----L---SRNDKANTLM---------------GILY 804

UrV3_RdRP -GDRNRARSARAVAVKCGFPPELLGSDDMWKAMAWLSPRDRSNMYAGLAERQST------ 739

YP_009333150.1 YPIDRRPTTEPEVFGRIGLTPAIVAAFGGIKRVLLYLPVNRLAKYVEPAPPQGLRLELYW 891

BAU79517.1 RAKTNTPTSEREAIARMGLDPSLVKYLGGVSKAWRLTDPHKLARYQEPVEPCALNPILYY 772

AAB94791.2 QRLTDDD--LRALVALVGGNTSAK----DIRAEAF-GAESSSST---------IMGILPH 812

UrV2_RdRp AHISRTL--LRTLVILAGGDPNVS----DLDAEAW-GPDNRSTI---------VQGSLSY 754

YP_009182167.1 SGLRDSD--VRELVREAGGDWTAR----DIGAEAW-GIPSRSRA---------ISGVASY 837

NP_620495.1 D--SSDP--LRALQVIL------------------------------------------- 731

YP_009342428.1 S--AQNP--MEFLRVIS------------------------------------------- 813

UrV1_regi_RdRp Q--SADP--MRLLRVVA------------------------------------------- 756

UrV4 Q--SKDP--MQLLKVIA------------------------------------------- 817

UrV3_RdRP ----TKGWRWEMPP-------------------LLRTD---------------------- 754

YP_009333150.1 EDSAIQHWHRTAGLSGEKAPRLADAVYARRWPRLQRSEATRDTTTRVLILAPNGAGKTTY 951

BAU79517.1 IDSALVSWMTATPHIPRDYRA--------------------------------------- 793

AAB94791.2 SDASNYCKRTRNGNIIVPYHIRS------------------------------------- 835

UrV2_RdRp ADAAALSNKATTGVLYTTYNVYM------------------------------------- 777

YP_009182167.1 TDAASLARRAETDVVFFPYPVHM------------------------------------- 860

NP_620495.1 ------------------------------------------------------------ 731

YP_009342428.1 ------------------------------------------------------------ 813

UrV1_regi_RdRp ------------------------------------------------------------ 756

UrV4 ------------------------------------------------------------ 817

UrV3_RdRP ------------------------------------------------------------ 754

YP_009333150.1 CRSHQWVFDSDEMLGKVVEKSSFRALRYRPDVERPSHVASAVEHILLRRDRYGIATQLDV 1011

BAU79517.1 ------------------------------------------------------------ 793

AAB94791.2 ------------------------------------------------------------ 835

UrV2_RdRp ------------------------------------------------------------ 777

YP_009182167.1 ------------------------------------------------------------ 860

NP_620495.1 ------------------------------------------------------------ 731

YP_009342428.1 ------------------------------------------------------------ 813

UrV1_regi_RdRp ------------------------------------------------------------ 756

UrV4 ------------------------------------------------------------ 817

UrV3_RdRP ------------------------------------------------------------ 754

YP_009333150.1 DLVSLMPSQRGWEWQIYIVQPPRAELETRLRARGWDDTKIARRLDHWEASVRHGVRKSKH 1071

BAU79517.1 ------------------------------------------------------------ 793

AAB94791.2 ------------------------------------------------------------ 835

UrV2_RdRp ------------------------------------------------------------ 777

YP_009182167.1 ------------------------------------------------------------ 860

NP_620495.1 ------------------------------------------------------------ 731

YP_009342428.1 ------------------------------------------------------------ 813

UrV1_regi_RdRp ------------------------------------------------------------ 756

UrV4 ------------------------------------------------------------ 817

UrV3_RdRP ------------------- 754

YP_009333150.1 LTSKEREGAHWCSEWPEKI 1090

BAU79517.1 ------------------- 793

AAB94791.2 ------------------- 835

UrV2_RdRp ------------------- 777

YP_009182167.1 ------------------- 860

NP_620495.1 ------------------- 731

YP_009342428.1 ------------------- 813

UrV1_regi_RdRp ------------------- 756

UrV4 ------------------- 817
